# Chemophenetic Significance of *Anomalocalyx uleanus* Metabolites Are Revealed by Dereplication Using Molecular Networking Tools

**DOI:** 10.3390/molecules26040925

**Published:** 2021-02-09

**Authors:** José Assis Gomes de Brito, Luciano da Silva Pinto, Cintia Folly Chaves, Antônio Jorge Ribeiro da Silva, Maria Fátima das Graças Fernandes da Silva, Fernando Cotinguiba

**Affiliations:** 1 Instituto de Pesquisas de Produtos Naturais “Walter Mors”, Centro de Ciencas da Saude, Universidade Federal do Rio de Janeiro (UFRJ), Avenida Carlos Chagas Filho, 373, Bloco H, Cidade Universitaria, CEP 21941-902 Rio de Janeiro, Brazil; jose.assis@ifro.edu.br (J.A.G.d.B.); cintiafolly@outlook.com (C.F.C.); ajorge@ippn.ufrj.br (A.J.R.d.S.); 2 Instituto Federal de Rondônia, Campus de Ji-Parana, Rua Rio Amazonas, 151, Jardim dos Migrantes, CEP 78960-000 Ji-Paraná-RO, Brazil; 3 Departamento de Quimica, Universidade Federal de São Carlos, Rodovia Washington Luís km 235, CEP 13565-905 São Carlos-SP, Brazil; lucianosilva58@gmail.com (L.d.S.P.); dmfs@ufscar.br (M.F.d.G.F.d.S.)

**Keywords:** Euphorbiaceae, *Anomalocalyx*, phenolic plant metabolism, molecular networking

## Abstract

*Anomalocalyx uleanus* (Pax & K. Hoffm.) Ducke (Euphorbiaceae) is a singular species in the genus and is restricted and exclusive to the Brazilian Amazon. A phytochemical study of *A. uleanus* leaves was performed, yielding the isolation of five major compounds: catechin/epicatechin, afzelin, quercetin 3-*O*-α-L-rhamnopyranoside, and astilbin. The phytochemical compositions of the methanolic extracts of leaves, roots, bark, and stem bark were determined using a dereplication approach. Forty-six compounds were annotated from the liquid chromatography-mass spectrometry (LC-MS/MS) data, while four lipids were identified using gas chromatography-mass spectrometry (GC-MS). In total, fifty compounds were detected, and they belonged to the primary metabolism and several classes of natural products such as flavonoids, flavonoids *O*-glycosides, flavonoids *C*-glycosides, biflavonoids, procyanidin, triterpene, triterpenes esterified with phenylpropanoids, phenylpropanoid derivatives, flavonolignans, coumarins, quinic acid derivatives, and benzoic acid derivatives. This is the first report on the phytochemical data of the genus *Anomalocalyx*, and the results of this study will contribute to the chemosystematic knowledge of the Euphorbiaceae family and justify the need for investigation of the pharmacological potential of the species *A. uleanus*.

## 1. Introduction

The Euphorbiaceae family comprises 219 genera and 6300 species, which are distributed in tropical and subtropical regions, and many of them are endemic to Brazil [1]. This plant family has a very diverse secondary metabolism, and their metabolites are characterized by their medicinal properties [2,3]. The species of the Euphorbiaceae family have suffered throughout history, with several reorganizations within the subfamilies, genera, and even segregations of other families. Chemosystematic and phylogenetic studies have contributed immensely to the understanding of the classification of this family [4,5].

Recently, the term chemophenetics has been used to describe a variety of secondary metabolites specialized in a given taxon and are important for the description of classified organisms with the help of modern molecular methods. Chemophenetics do not aim to elucidate phylogenetic relationships but to describe the matrix of natural products and use them for the phenetic characterization of clades [6]. Several studies using chemophenetic and chemotaxonomic approaches have been described in the literature, including several genera of the Euphorbiaceae such as *Senefelderopsis* [7], *Euphorbia* [8,9,10], *Mallotus* [5], *Chrozophora* [11], *Croton* [12], *Alchornea* [13], *Sapium* [14], and *Sebastiania* [15], among other examples. 

*Anomalocalyx uleanus* (Pax & K. Hoffm.) Ducke is a unique species in the genus *Anomalocalyx*, and it is popularly known as “arataciú-preto”. It is a tree that can reach 40 m, has no latex, and is restricted and exclusive to the Brazilian Amazon. This species is found mainly in flooded areas and has never been studied chemically [3]. Metabolite profiling using high-resolution mass spectrometry (HRMS) grants access to large volumes of high-quality spectral data from a minimal amount of samples, and appropriate data analysis workflows allow the efficient mining of such data [16]. An approach to dereplicating the extracts of leaves, roots, bark, and stem bark of *A. uleanus* was adopted in order to discover as much as possible the classes of natural products present in that species using tools such as MZmine 2, MS-DIAL, MSFINDER, and GNPS. The aim of this work was to describe, as extensively as possible, the array of natural products present in *A. uleanus* and to establish chemophenetic correlations with other species in Euphorbiaceae [3,17]. 

## 2. Results and Discussion

### 2.1. Isolation of Majoritary Compound from A. uleanus Leaves

A phytochemical study of *A. uleanus* leaves was performed using traditional approaches for extract preparation; liquid-liquid partition, thin layer chromatography, and high-performance liquid chromatography were performed. The ethyl acetate partition was used for separation of the major compounds using high-performance liquid chromatography (HPLC), and five compounds were isolated—namely, catechin (**5a**), epicatechin (**5b**), afzelin (**12**), quercetin 3-*O*-α-L-rhamnopyranoside (**15**), and astilbin (**16**). The purified compound samples were analyzed using ^1^H-NMR and MS, and the experimental data were compared with data from the literature to confirm their structures. These five compounds were classified as level L1 identification, because their structures were characterized using NMR spectroscopy (see Table 1) [18,19].

### 2.2. Dereplicated Compounds from A. uleanus of Methanolic Roots, Bark, Stem Bark, and Leaf Extracts of A. uleanus and the Fragmentation Proposed to the Main Compounds

**Figure 1:**
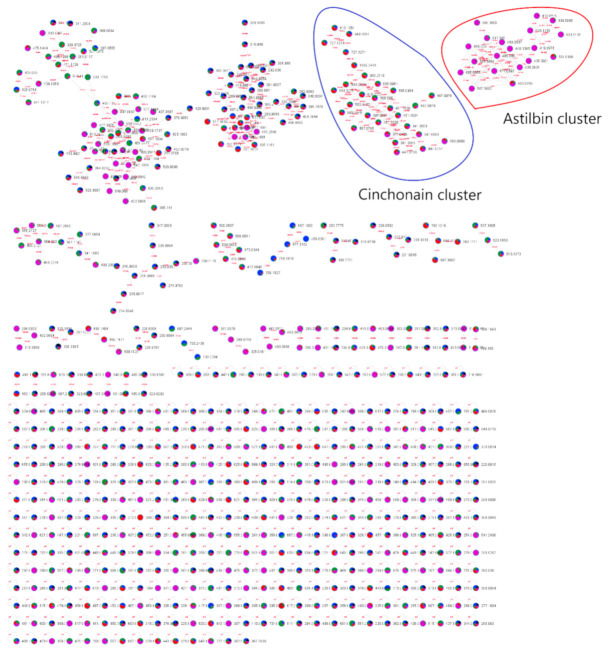
Molecular networking of different methanolic extracts from *Anomalocalyx uleanus* with MS^2^ data obtained in the negative mode and clustered by the Cytoscape 3.80 software and emphasizing the main compound annotations obtained from the GNPS and METLIN libraries. Color nodes: roots (blue), bark (green), stem bark (red), leaves (purple), and blank (black).

The methanolic extracts of roots, bark, stem bark, and leaves from *A. uleanus* were analyzed using Ultra-High Performance Liquid Chromatography with Quadrupole Time-of-Flight Mass Spectrometry (UPLC-QTOF-MS/MS) to obtain MS^2^ spectra data of compounds. Thus, MS^2^ data were converted using MSConverter and processed using MassHunter and MS-DIAL software to submit them to the GNPS and METLIN libraries to create Feature-Based Molecular Networking (FBMN) (Figure 1), which was visualized using Cytoscape v.3.8.0. The MN annotation was verified using a mass accuracy error below 5 ppm, and the fragmentation mechanism was proposed to the main compound. 

**Figure 2:**
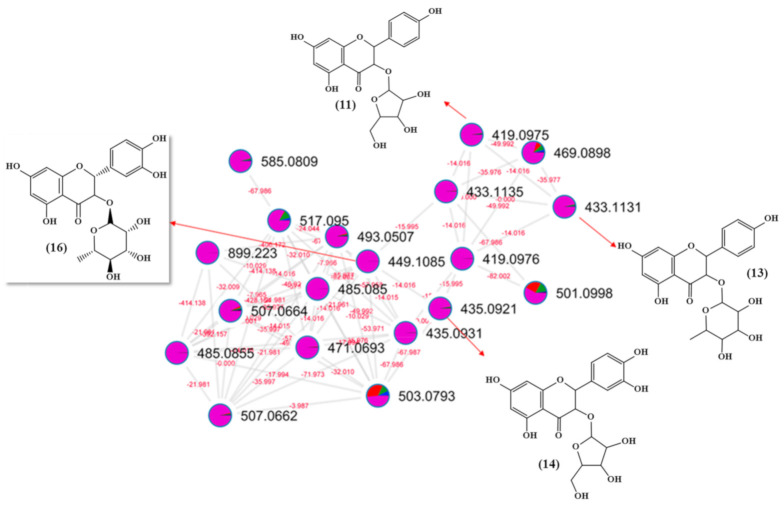
Astilbin (**16**) molecular network: molecular family of flavonol glycosides and phenolic compounds from a methanolic extract of *A. uleanus*. Color nodes: roots (blue), bark (green), stem bark (red), leaves (purple), and blank (black).

**Figure 3:**
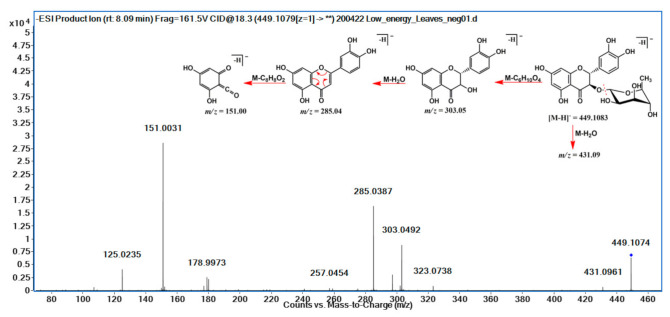
Proposed fragmentation mechanism of flavonoid *O*-glycoside astilbin (**16**).

A purified astilbin sample (**16**), after being structurally characterized by 1D and 2D NMR spectroscopy data, was used as a reference in the dereplication of other similar metabolites in the cluster network (Figure 2). Initially, the fragmentation mechanism for astilbin was proposed, and then, the values of the fragment ions were compared with the data of the other nodes of the same cluster. Derivatives of flavonols have fragmentation characteristics, such as loss of the sugar molecule, elimination of water, and a Retro-Diels-Alder reaction. It was noticed that the nodes in this cluster were all purple in color (referring to the leaves), showing that all substances were main present in the plant organs (Figure 3). 

**Figure 4:**
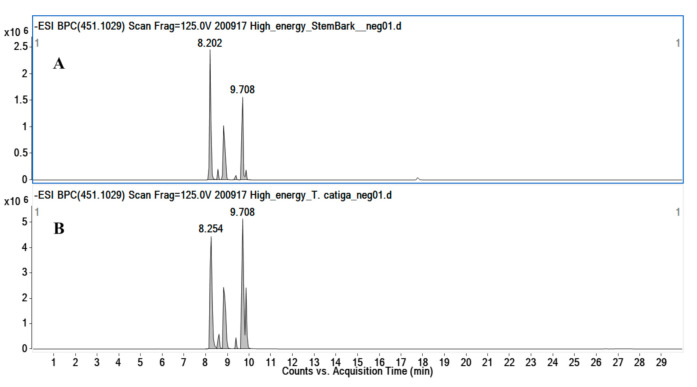
Extracted ion chromatogram (EIC) of [M − H]^−^ = 451.1029 for cinchonain Ib (**33**) from stem bark (**A**) and the crude extract of *Trichilia catigua* (**B**).

**Figure 5:**
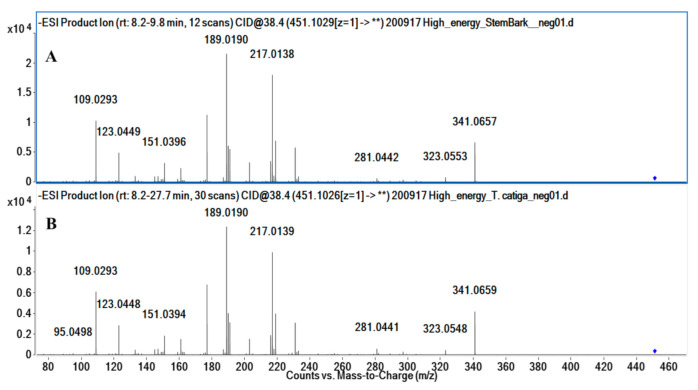
MS^2^ spectra of [M − H]^−^ = 451.1029 for cinchonain Ib (**33**) from the stem bark of *A. uleanus* (**A**) and crude extract of *T. catigua* (**B**) at the same collision energy.

**Figure 6:**
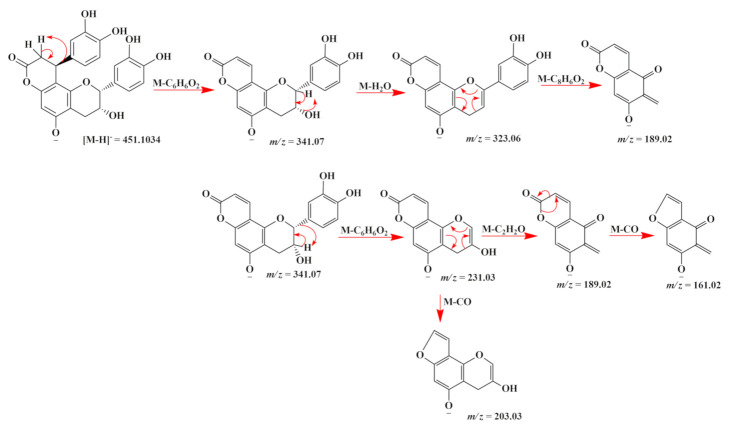
Proposed fragmentation mechanism of cinchonain Ib (**33**) from the *A. uleanus* and *T. catigua* crude extracts.

Flavonolignans was another class of natural products detected in the extracts of *A. uleanus*. The most evident was cinchonain Ib (**33**), which is one of the bioactive compounds found in catuaba (*Trichilia catigua*) [20]. A comparison was made between the extracts from *A. uleanus* and *T. catigua*, and both showed exactly the same MS signals from the base peak chromatogram (BPC) (Figure 4), and the molecular formula was calculated under a mass error below 3 ppm to give C_24_H_20_O_9_, [M − H]^−^ = 451.1030. Thus, the MS^2^ spectra data comparison showed equal signals from the fragment mechanism (Figure 5). The fragmentation mechanism proposed is shown below with the first loss of the catechol unit (C_6_H_6_O_2_), then to the dehydration reaction (M-H_2_O) to give *m/z* = 323.06, and, lastly, to the Retro-Diels–Alder reaction (RDA) at the C ring to give fragment ions at *m/z* = 189.02. In addition, two catechol loss sequentially to give fragment ions at *m/z* = 231.03, then carbon monoxide loss to give 203.03 or an RDA fragmentation reaction at the C ring to give *m/z* = 189.02, and, lastly, carbon monoxide loss to give *m/z* = 162.02 (Figure 6).

The comparison of the base peak chromatogram (BPC) from the extract and stem bark of *A. uleanus* (A) and *T. catigua* (B) showed retention times that support the presence of the same substance as observed in the fragmentation profile for the same ion in their respective MS^2^ spectra. 

**Figure 7:**
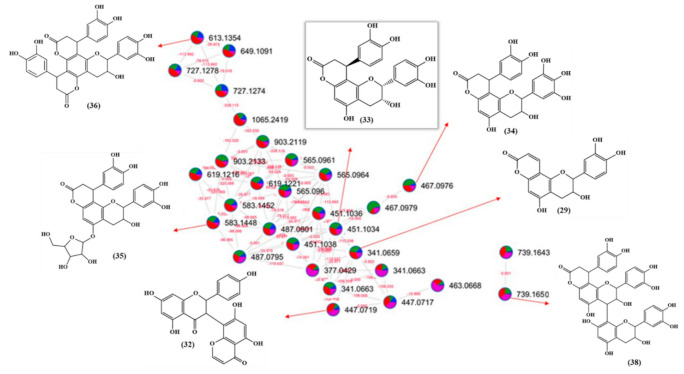
Molecular network of the molecular family of cinchonain Ib (**33**) and derivatives from the methanolic extract of *A. uleanus*. Color nodes: roots (blue), bark (green), stem bark (red), leaves (purple), and blank (black).

The fragmentation profile observed for cinchonain Ib (Figure 6) and the fragmentation proposal supported the annotation of the other substances, as shown in Figure 7. The monitoring of the fragment ions *m*/*z* 341.07, 217.01, and 189.02 were key points to confirm the similar fragmentation profile information in the nodes of the molecular network presented in Figure 7, and the direct differences between each node helped in the proposal of the other structures.

In addition to flavonoid aglycones, *O*-glycosylated flavonoids, *C*-glycosylated flavonoids, and flavonolignans, other classes of natural products such as biflavonoids, procyanidin, triterpene, triterpenes esterified with phenylpropanoids, phenylpropanoid derivatives, coumarins, quinic acid derivatives, and benzoic acid derivatives were also identified. 

Gas chromatography-mass spectrometry (GC-MS) was also used to generate data on the chemical composition of *A. uleanus*. The hexane partition of the leaf extract was fractionated with silica gel (adsorption chromatography), and the 9D subfraction was derivatized for analysis using GC-MS. The GC-MS data were compared with the NIST library, making it possible to identify four fatty acid esters: palmitic acid (methyl ester) (**47**), linoleic acid (methyl ester) (**48**), stearic acid (methyl ester) (**49**), and oleic acid (methyl ester) (**50**). 

**Figure 8:**
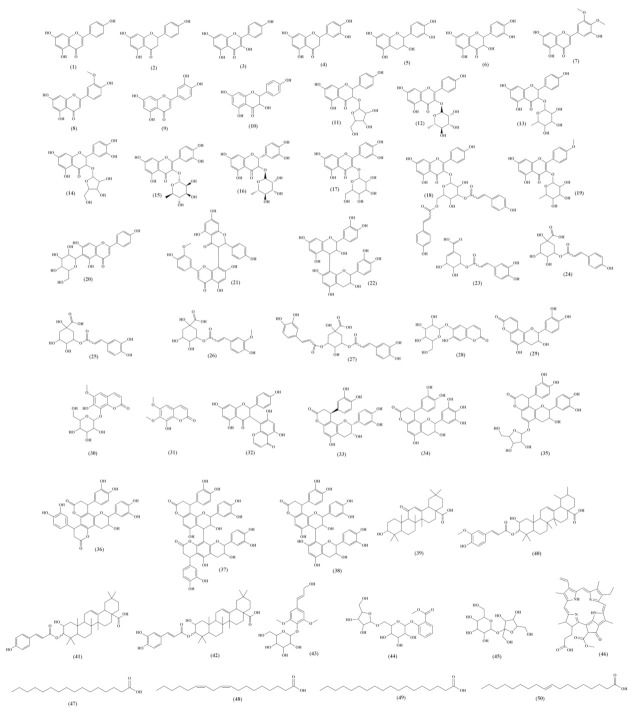
Dereplicated compounds from *A. uleanus* leaves, roots, bark, and stem bark extracts.

Then, forty-six compounds were determined using LC-MS/MS data, while four lipid derivatives were determined using GC-MS, totaling fifty compounds identified from extracts of different parts of *A. uleanus*. These compounds were classified with the following levels of identification: level 1 (L1): structure confirmed by the reference standard or elucidated using NMR spectroscopy, level 2a (L2a): probable structure using library spectrum match, and level 3 (L3): tentative candidates based on MS and MS2 experimental data [18,19] (Figure 8).

### 2.3. Chemophenetic Significance

The species of the Euphorbiaceae family have high chemical complexity and accumulate a series of metabolites from different classes of natural products.

Flavonoids and O-glycosylated flavonoids are compounds present in several genera of Euphorbiaceae, such as *Alchornea* [21], *Chrozophora* [11], *Cnidoscolus* [22], *Croton* [23], *Euphorbia* [24], *Macaranga* [25], *Phyllanthus* [26], *Jatropha* [27], *Ricinus* [28], *Pedilanthus* [29], and others. Several flavonoids, such as apigenin (**1**), naringenin (**2**), kaempferol (**3**), catechin/epicatechin (**5**), and quercitrin (**15**), identified in *A. uleanus* are present in most of the genera mentioned above. However, flavonoids eriodictyol (**4**) and astilbin (**16**) have more restricted distributions within the Euphorbiaceae family. Eriodictyol (**4**) is a flavonoid widely distributed in the plant kingdom; however, in the Euphorbiaceae family, it was identified only in three species of the genus *Phyllanthus*: *P. emblica* [30], *P. niruri* [31], and *P. amarus* [32]. *Phyllanthus* is placed in the subfamily Phyllanthoideae and has been classified in the tribe Phyllantheae, which was divided into six subtribes and 18 genera [33]. A prenylated derivative of eriodictyol has also been identified in one species of the genus *Macaranga*: *M. triloba* [34]. Astilbin (**16**) is present in considerable amounts in the leaves of *A. uleanus.* Despite having a relatively common structure, it is the second time that this glycosylated flavonoid has been identified in the Euphorbiaceae family. Until that time, this substance has only been identified in species of the genus *Mallotus*: *M. apelta* and *M. metcalfianu* [35]. 

Coincidentally, *Mallotus, Macaranga, and Phyllanthus* genera share chemical similarities. *Macaranga* and *Mallotus* are closely related, are large paleo(sub)tropical genera, both share very similar ecological strategies, and have similar geographical distributions and a recent common ancestry [36]. *Mallotus* and *Phyllanthus* genera have been extensively used in folk medicine in India, China, Vietnam, and other countries for thousands of years for the treatment of a broad spectrum of diseases, such as chronic hepatitis, enteritis, urinary bladder, intestinal infections, and kidney disease. Thiangthum et al., using tools such as HPLC and multivariate analysis, analyzed the similarities and differences in the compositions of compounds having antioxidant properties from 36 samples from six species of *Mallotus* and *Phyllanthus*. The results were used to compare and differentiate species from both genera [37].

One *C*-glycosylated flavonoid, isovitexin (**20**), was also detected in the extracts of *A. uleanus*. This class of compound is found in some genera of Euphorbiaceae, such as *Aleurites* [38], *Croton* [39], *Jatropha* [40], and *Phyllanthus* [41]. One biflavonoid was also annotated as 3′′′-*O*-methylfukugetin (**21**). Biflavonoids also occur in the Euphorbiaceae family, in the genera *Euphorbia* [42] and *Senefelderopsis* [43].

Procyanidins (**22**) are dimers and higher polymers formed from catechin and epicatechin molecules that occur in several plant families, including Euphorbiaceae. This class of compounds can be found in several species of the genera, such as *Croton* [2], *Antidesma*, and *Phyllanthus* [44].

Flavonolignans are a very rare class of natural products in Euphorbiaceae and have been identified just once in a species. Rivière et al. identified a mixture of two pairs of new diastereoisomeric flavonolignans (±)-hydnocarpin-7-*O*-(4″-*O*-(*E*)-coumaroyl)-*β*-glucopyranoside)/(±)-hydnocarpin-D-*7*-*O*-(4″-*O*-(*E*)-coumaroyl)-*β*-glucopyranoside) in a ratio of 2:1 in *Mallotus metcalfianus* [45]. The identification of this class of natural products reiterates the need for chemotaxonomy, since they are the only two records indicating the presence of flavonolignans in species of the Euphorbiaceae family.

Despite the presence of flavonolignans in Euphorbiaceae, this is the first report of cinchonain Ib (**33**) in the family. This bioactive compound is found in the bark of *Trichilia catigua* (Meliaceae). An infusion of the bark, which is called catuaba, is used in traditional Brazilian medicine as an aphrodisiac and central nervous system stimulant [20]. The families Euphorbiaceae and Meliaceae belong to the Rosids group; Meliaceae is included in the order Sapindales, while Euphorbiaceae is included in the order Malpighiales [46]. In addition to Meliaceae, cinchonain Ib (**33**) has already been identified in several families, such as Elaeagnaceae [47], Hypericaceae [48], Lauraceae [49], Rhizophoraceae [50], Rosaceae [51], Rubiaceae [52], Smilacaceae [53], and Theaceae [54].

Chlorogenic acid (**25**) and its derivatives are present in Euphorbiaceae and could be isolated from some species of Euphorbiaceae, such as *Jatropha aethiopica* [55], *Euphorbia peplus* [56], *Euphorbia hirta*, *Phyllanthus emblica*, *Ricinus communis* [57], *Sapium insigne* [58], and *Croton antisyphiliticus* [39]. Coumarins are relatively common in many Euphorbiaceae genera, such as *Pedilanthus* [59], *Cnidoscolus* [22], *Macaranga* [60], *Mallotus* [61], *Phyllanthus* [62], *Jatropha* [63], *Euphorbia* [64], and others. Four coumarins were detected in *A. uleanus*: aesculin (**28**), phyllocoumarin (**29**), fraxin (**30**), and fraxidin (**31**). The compounds **40**, **41**, and **42** were annotated as triterpenes esterified with phenylpropanoids. Triterpenes esterified with phenylpropanoids can be found in several species of the following genera: *Cnidoscolus* [65], *Drypetes* (Putranjivaceae family) [66], *Glochidion* (Phyllanthaceae family) [67], and *Jatropha* [68]. 

## 3. Materials and Methods

### 3.1. Plant Material

On 4 May 2017, samples of the *A. uleanus* species was collected at the Jarú Biological Reserve (REBIO-Jarú) in Ji-Paraná (RO), Brazil. The samples were left in a bleach solution (5%) for 5 min and then washed with running water. Fresh leaves (1029 g), bark (242 g), stem bark (147 g), and roots (123 g) were separated and dried in a circulating air oven for 15 days at 37 °C. Following this, all the materials were crushed separately in a mill. The collected plants were previously identified by a team of botanists from the Jarú Reserve. An exsiccate (number RB01331744) of this species was deposited in the herbarium of the Rio de Janeiro Botanical Garden (JBRJ), and its identification was confirmed again by Prof. Cássia Mônica Sakuragui (Institute of Biology/UFRJ/Rio de Janeiro, Brazil).

### 3.2. Preparation of Crude Extracts and Fractionation 

Crude extracts were prepared using methanol (HPLC grade) by maceration for three times during three days. The extracts were concentrated using a reduced pressure in a rotary evaporator R-100 (Büchi, Flawil, Switzerland). The extracts from the bark (BMe), stem bark (TBMe), roots (RMe), and leaves (LMe) were subject to chromatographic separations via solid-phase extraction (SPE) using C-18 as the stationary phase and acetonitrile and ultrapure water as the mobile phases. To isolate the compounds, the crude leaves extract was resuspended in a mixture of water/methanol (1:1), followed by extraction with hexane and ethyl acetate. The compounds in the ethyl acetate fraction were separated by HPLC (see Section 3.3, Purification of compounds). For the study on dereplication, see Section 3.5 (UPLC-MS/MS analysis for molecular networking).

### 3.3. Purification of Compounds 

The ethyl acetate fraction (204 mg) was dissolved in 1.5 mL of acetonitrile/ultrapure water solution (20:80), acidified with 1% formic acid, and subsequently, injected for semipreparative HPLC. HPLC analysis was performed on a Dionex UltiMate 3000 system (Thermo Scientific, Waltham, MA, USA). A C18 Inertsil ODS-4 column (5 µm × 6.0 × 250 mm^2^) was used. Ultrapure water (with 1% formic acid) and methanol (with 1% formic acid) were used as the mobile phase. Samples were separated by gradient elution under the following conditions: t = 0 min, 10% B; t = 40 min, 33% B; t = 41 min, 100% B; t = 56 min, 100% B; and t = 57 min, 10% B. The flow rate was 2.8 mL/min, and the wavelength (λ) was 280 nm. Samples corresponding to five peaks were collected and subjected to ^1^H-NMR analysis. 

### 3.4. Structure Elucidation by ^1^H-NMR Spectroscopy

^1^H-NMR spectra were obtained using the 400- and 500-MHz VNMR-500 (Varian, Palo Alto, CA, USA) NMR spectrometer. The spectra were calibrated using tetramethylsilane (TMS). MestReNova software (version 6.0.2) was used to process the spectra. Coupling constants and chemical shifts were expressed in Hz and parts per million (ppm), respectively.

#### 3.4.1. Catechin (**5a**)

Brownish yellow amorphous powder (1.4 mg). QTOF-MS/MS spectrum (negative ionization mode) displayed a molecular ion peak at *m*/*z* 289.0720 [M−H] (calculated for C_15_H_14_O_6_, 290.0790). ^1^H-NMR (500 MHz, CD_3_OD): δ 6.84 (*d*, *J* = 1.8 Hz, H-2′), δ 6.76 (*d*; *J* = 8.1 Hz, H-5′), δ 6.72 (*dd*; *J* = 8.1 and 1.8 Hz, H-6′), δ 5.85 (*d*; *J* = 2.2 Hz, H-6), 5.92 (*d*, *J* = 2.2 Hz, H-8), δ 4.56 (*d*; *J* = 7.7 Hz, 1H, H-2), δ 3.97 (*dt*, *J* = 7.7 and 5.5 Hz, H-3), δ 2.84 (*dd*, *J* = 16.0 and 5.4 Hz, H-4b), δ 2.50 (*dd*, *J* = 16.0 and 8.4 Hz, H-4a). These data are consistent with the catechin structure reported in the literature [69].

#### 3.4.2. Epicatechin (**5b**)

Brownish yellow amorphous powder (1.1 mg). QTOF-MS/MS spectrum (negative ionization mode) displayed a precursor ion peak at *m*/*z* 289.0720 [M − H]^−^ (calculated for C_15_H_14_O_6_, 290.0790). ^1^H-NMR (500 MHz, CD_3_OD): δ 5.94 (*d*, *J* = 2.0, Hz, H-6) and 5.91 (*d*, *J* = 2.0 Hz, H-8), δ 6.97 (*d*, *J* = 1.3 Hz, H-2′), δ 6.76 (*d*; *J* = 8.1 Hz, H-5′), δ 6.80 (*dd*; *J* = 8.1 and 1.3 Hz, H-6′), δ 4.82 (*br s*, 1H, H-2), δ 4.18 (*br s*, H-3), δ 2.86 (*dd*, *J* = 16.9 and 4.6 Hz, H-4a), 2.73 (*dd*, *J* = 16.9 and 2.7 Hz, H-4b). These data are consistent with the epicatechin structure reported in the literature [70]. 

#### 3.4.3. Afzelin (**12**)

Yellow powder (2.8 mg). QTOF-MS/MS spectrum (negative ionization mode) displayed a precursor ion peak at *m*/*z* 431.0977 [M − H]^−^ (calculated for C_21_H_20_O_10_, 432.1056). ^1^H-NMR (400 MHz, CD_3_OD): δ 7.77 (*d*, *J* = 8.7 Hz, H-2′ and H-6′), δ 6.38 (*d, J* = 1.9 Hz, H-6), δ 6.21 (*d*, *J* = 1.9 Hz, H-8), δ 6.94 (*d*, *J* = 8.7 Hz, H-3′ and H-5′), δ 5.38 (*d*, *J* = 1.5 Hz, H-1″), δ 4.22 (*m*, H-5”), δ 3.71 (*m*, H-3″), δ 3.48 (*m*, H-2″), δ 3.33 (*m*, H-4″), δ 0.92 (*d*; *J* = 5.6 Hz, H-6″). These data are consistent with the afzelin structure reported in the literature [71].

#### 3.4.4. Quercetin (quercetin 3-*O*-α-L-rhamnopyranoside) (**15**)

Yellow powder (1.1 mg). QTOF-MS/MS spectrum (negative ionization mode) displayed a precursor ion peak at *m*/*z* 447.0926 [M − H]^−^ (calculated for C_21_H_20_O_11_, 448.10056). ^1^H-NMR (400 MHz, CD_3_OD): δ 7.34 (*d*, *J* = 1.9 Hz, H-2′), δ 7.31 (*dd*, *J* = 8.2 and 1.9 Hz, H-6′), δ 6.91 (*d*, *J* = 8.2, H-5′), 6.37 (*d*, *J* = 1.5 Hz, H-8), δ 6.20 (*d*, *J* = 1.5 Hz, H-6), δ 5.35 (*d*, *J* = 1.5 Hz, H-1″), δ 4.22 (*m*, H-2″), δ 3.42 (*m*, H-5″), δ 3.75 (*dd*, *J* = 9.3 and 3.2, H-3″), 3.33 (*br s*, H-4″), δ 0.94 (*d*, *J* = 6.1, H-6″). These data are consistent with the quercetin structure reported in the literature [72].

#### 3.4.5. Astilbin (**16**)

Yellowish-green amorphous powder (7.4 mg). QTOF-MS/MS spectrum (negative ionization mode) displayed a molecular ion peak at *m*/*z* 449.1083 [M − H]^−^ (calculated for C_21_H_22_O_11_, 450.1162). ^1^H-NMR (400 MHz, CD_3_OD): δ 6.96 (*d*, *J* = 1.7 Hz, H-2′), δ 6.82 (*m*, H-5′and H-6′), δ 5.92 (*d*, *J* = 2.0 Hz, H-8), δ 5.90 (*d*, *J* = 2.0 Hz, H-6), δ 5.07 (*d*, *J* = 10.7, H-2), δ 4.58 (*d*, *J* = 10.7, H-3), δ 4.25 (*m*, H- 2″), δ 4.04 (*d*, *J* = 1.6 Hz, H-1″), δ 3.66 (*dd*, *J* = 9.5 and 3.3, H-3″), 3.54 (*dd*, *J* = 3.3 and 1.7, H-4″), δ 3.33 (*br s*, H-5″), δ 1.19 (*d*, *J* = 6.2, H-6″). These data are consistent with the astilbin structure reported in the literature [73].

### 3.5. UPLC-MS/MS Analysis for Molecular Networking 

MS^1^ and MS^2^ analyses were performed on an Agilent 6545 Q-TOF mass spectrometer (Agilent Technologies Inc., Santa Clara, CA, USA) equipped with an electrospray ionization (ESI) source. MassHunter^®^ workstation software (version B.08.00) was used for data acquisition and processing. The samples were analyzed in triplicate of authentic biological replicates. The chromatographic separation was carried out on an Agilent Zorbax SB-C18 column (3.0 × 50 mm) and water–acetonitrile with 0.1% formic acid (LC-MS grade). The mobile phase flowed at a rate of 0.3 mL/min after injecting 5 µL of the analytical solutions. The mass data were acquired with a positive (+) and negative (−) ion ESI source in the TOF-MS mode for the molecular ions and in the auto-MS/MS mode for the fragment ions using collision energy (Table S2, Supplementary Materials). The operating source parameters for the TOF-MS mode were as follows: capillary voltage, 2.400 KV; skimmer voltage, 65 V; fragmentor voltage, 110 V; nebulizer gas pressure, 28 psi; dry gas flow, 10 L/min; gas temperature, 300 °C; sheath gas flow, 10 L/min; sheath gas temperature, 350 °C, acquisition rate, 3 spectrum per second; and a resolution of 32,000. The mode of acquisition of auto-MS or target MS/MS and the acquisition of second-order spectra followed pre-established processes of collision energies in the form of mini-ramps of small intervals of *m/z*—100−300 Da, for example (Table 1). The acquired data were processed by the MassHunter^®^ molecule features extraction software to find the compounds, and the ionized molecules ([M + H]^+^ and [M − H]^−^) obtained in the TOF-MS mode were identified. 

### 3.6. Molecular Networking Full Imaging

The molecular networking visualization (MN) was constructed using each MS2 spectrum as a node, and the connection between nodes were made by the mass difference from the precursor ion (edges); then, each individual molecular networking has a similar fragmentation profile with a minimum fragment-ion characteristic. Further, Figure 1 suggests different colors of the crude extracts of the roots (blue), bark (green), stem bark (red), leaves (purple), and blank (black). The annotation of the secondary metabolites was performed using the attempt to rationalize the fragment ions by means of fragmentation proposals applying concepts of organic chemistry, with an emphasis on acid/base mechanisms together with several databases for secondary metabolites (FooDB, PlantCyc, ChEBI, LipidMAPS, DrugBank, KNApSAcK, NANPDB, PubChem, UNPD, and METLIN). For this purpose, deconvolution of the MS^2^ spectra and peak alignment was performed using the MS-Dial software (http://prime.psc.riken.jp/compms/msdial/main.html). Then, the processed, deconvolved, and aligned MS^2^ spectra were extracted and submitted for processing in the MS-FINDER software in order to achieve a greater number of databases aimed at the annotation of the secondary metabolites Aiming to process at MS-DIAL, MS^1^ at ±0.02 Da and MS^2^ at ±0.06 Da mass tolerance, minimum peak height of 1 × 10^4^, MS/MS amplitude abundance cut-off at 30, retention time tolerance of ±0.5 min, and MS1 tolerance of ±0.02 Da were used to align the chromatogram. Thus, the processed data were exported to the .mgf format postprocessing and subjected to a feature-based molecular networking (FBMN) analysis at the global natural product social molecular networking (GNPS) platform (https://gnps.ucsd.edu/). The confidence annotated metabolites are presented in this site: https://gnps.ucsd.edu/ProteoSAFe/status.jsp?task=deca49b0f49d4af087be4e852e9a4b91). The MN was processed using Cytoscape version 3.8.0 software (https://cytoscape.org/) to construct the molecular network. Parent ions for all the extracts were different. Besides, 5 fragment ions were used to construct the molecular network, and 4 fragment ions were used for library annotation.

### 3.7. Comparison between A. uleanus Extracts and Cinchonain Ib Pattern from Trichilia catigua (catuaba) 

A commercial sample of catuaba tea (*Trichilia catigua*, brand “Chá do Brasil”^®^) was used to prepare the extracts following the protocol described by Beltrame et al. (2006) [20]. *A. uleanus* extract and catuaba standard extract containing cinchonain Ib were subjected to the same chromatographic conditions, ionization energy, and collision energy. The chromatographic separation was performed on a reverse column using the Agilent 1200 system. The column temperature and injection volume were 33 °C and 3 µL, respectively. The proportion of solvent B (acetonitrile) was linearly varied as follows: 0–4 min, 5–15%; 4–15 min, 15–60%; 15–24 min, 60–100%; 24–27 min, 100% (isocratic elution); 27–27.50 min, 100–5%; and 27.50–30 min, 5% (isocratic elution). The ESI source conditions were supported by the mass ranges 200–1500 Da to MS^1^ and 70–1500 Da to MS^2^ data. The other parameters were as follows: capillary voltage, 2.5 kV; nozzle voltage, 0 (zero); fragmentor voltage, 125 V; skimmer voltage, 65 V; octupole RF peak, 750 V; gas temperature, 300 °C; gas flow, 12 L/min; nebulizer pressure, 35 psi; sheath gas temp, 350 °C; and sheath gas flow, 10 L/min. MS^2^ spectra acquisition was performed under the following collision energies: 100–300 Da (30–35 eV), 300–500 Da (35–40 eV), 500–700 Da (40–45 eV), 70–1000 Da (45–50 eV), and 100–1500 Da (50–60 eV).

### 3.8. GC-MS Analysis

Sample 9D (58 mg), obtained from the hexane fraction of *A. uleanus* leaves, was dissolved in 4 mL of 0.5-N NaOH solution in methanol and subjected to methylation according to the procedure described by Ichihara and Fukubayashi (2010). This derivatized form of sample 9D was analyzed on a GC-MS chromatograph (Shimadzu, GCMS-QP2010 model, Kyoto, Japan), using a DB-5 MS column (30 m × 0.25 mm × 0.25 µm). The chromatography was conducted in the split injection mode, with a column oven temperature of 100 °C and injection temperature of 225 °C. Column flow rate was 1 mL/min, equilibration time was 3.0 min, and helium was used as the carrier gas. The results were compared with the NIST library database, and it was possible to identify four esters (**47**–**50**) [74].

## 4. Conclusions

The chemical composition of *A. uleanus* was investigated using both phytochemical and dereplication approaches. Five major compounds were isolated from the leaf extracts and identified using NMR and MS: catechin/epicatechin (**5a**/**5b**), afzelin (**12**), quercetin 3-*O*-*α*-L-rhamnopyranoside (**15**), and astilbin (**16**). All the compounds were used as reference standards (level 1 of confirmation, L1) and were important in confirming the accuracy of the MS/MS data in the dereplication study. 

UPLC-Q-TOF-MS/MS and GC-MS analyses provided abundant information for the genus *Anomalocalyx*. It was the first time that the chemistry of the genus was described in the literature. Dereplication was the main investigative process adopted to avoid the re-isolation of the compounds already described, revealing the metabolic pathways present in *A. uleanus*. The use of the molecular networking approach is extremely important and powerful as a strategy for analyzing clusters of the main compounds. It was possible to establish a structural relationship between these known substances and other structures present in the cluster by analyzing the fragmentation pattern. Forty-six compounds were annotated from the LC-MS/MS data and four from GC-MS, totaling fifty compounds. These compounds belong to the primary metabolism and several classes of natural products, such as flavonoids, flavonoids *O*-glycosides, flavonoids C-glycosides, biflavonoids, procyanidin, triterpene, triterpenes esterified with phenylpropanoids, phenylpropanoid derivatives, flavonolignans, coumarins, quinic acid derivatives, and benzoic acid derivatives. 

One of the compounds annotated in the dereplication study was flavonolignan cinchonain Ib (**33**). This compound was obtained from *Trichilia catigua* bark extract, and it was used as a standard to confirm its presence and derivatives in *A. uleanus* barks by comparing MS spectral data. This is the first time that cinchonain Ib (**33**) and derivatives were detected in a species of the Euphorbiaceae family.

All the information obtained about the metabolism of the genus *Anomalocalyx* will contribute greatly to the chemosystemic and chemophenetic knowledge of the genus and suggest the need for its pharmacological potential, which has never been investigated, to be investigated.

## Figures and Tables

**Table 1:** Compounds detected in the *Anomalocalyx uleanus* methanolic extracts by UPLC-Q-TOF-MS/MS (compounds **1**–**46**) and gas chromatography-mass spectrometry (GC-MS) (**47**–**50**). Level of confirmation: Level 1 (L1): structure confirmed by reference standard or structure elucidation by NMR spectroscopy, level 2a (L2a): probable structure by library spectrum match, and level 3 (L3): tentative candidates based on MS and MS^2^ experimental data [18,19].

No.	Annotation	RT	Formula	Identification Confidence	MS	Error (ppm)	MS/MS
*Flavonoids*							
**1**	apigenin	11.67	C_15_H_10_O_5_	L2a	269.0451 [M − H]^−^	0.4	225.05; 201.06; 181.07; 151.00; 117.04
**2**	naringenin	11.63	C_15_H_12_O_5_	L2a	271.0610 [M − H]^−^	1.5	185.06; 151.00; 119.05; 107.01
**3**	kaempferol	10.53	C_15_H_10_O_6_	L2a	285.0399 [M − H]^−^	0.0	223.04; 183.04; 151.00; 133.03; 107.02
**4**	eriodictyol	8.04	C_15_H_12_O_6_	L2a	287.0551 [M − H]^−^	−1.7	177.02; 133.03; 109.03
**5a**/**5b**	catechin/epicatechin	6.74	C_15_H_14_O_6_	L1	289.0720 [M − H]^-^	2.4	173.06; 151.04; 137.02; 123.05; 109.03
**6**	taxifolin	8.35	C_15_H_12_O_7_	L2a	303.0506 [M − H]^−^	0.3	285.04; 217.05; 181.01; 177.02; 137.02; 125.02
**7**	apometzgerin	8.66	C_17_H_14_O_7_	L2a	331.0808 [M + H]^+^	−3.0	316.06; 301.03; 288.06; 273.04; 245.04; 167.03; 153.02
**8**	chrysoeriol	8.66	C_16_H_12_O_6_	L2a	301.0704 [M + H]^+^	−2.7	286.05; 269.04; 285.05; 153.02
**9**	luteolin	10.55	C_15_H_10_O_6_	L2a	285.0397 [M − H]^−^	−0.7	175.04; 151.00; 133.03
**10**	aromadendrin	8.01	C_15_H_12_O_6_	L2a	287.0553 [M − H]^−^	−1.0	259.06; 125.02
*Flavonoids O-glycosides*							
**11**	3-(arabinofuranosyloxy)-2,3-dihydro-5,7-dihydroxy-2-(4-hydroxyphenyl)-4H-1-benzopyran-4-one	8.14	C_20_H_20_O_10_	L2a	419.0980 [M − H]^−^	0.5	287.06; 269.05; 259.06; 180.00; 152.01; 151.00; 125.02; 107.01
**12**	afzelin	9.86	C_21_H_20_O_10_	L1	431.0977 [M − H]^−^	−0.2	285.04; 151.00
**13**	naringenin 3-*O*-glucoside	8.92	C_21_H_22_O_10_	L2a	433.1136 [M − H]^−^	0.2	287.06; 269.05; 259.06; 180.01; 152.01; 151.00; 125.02; 107.01
**14**	taxifolin 3-xyloside	7.47	C_20_H_20_O_10_	L2a	435.0930 [M − H]^−^	0.7	417.08; 309.06; 303.05; 285.04; 259.06; 151.00; 125.02; 107.01
**15**	quercitrin	8.47	C_21_H_20_O_11_	L1	447.0926 [M − H]^−^	−0.2	301.04; 300.03; 255.03; 151.00
**16**	astilbin	8.25	C_21_H_22_O_11_	L1	449.1083 [M − H]^−^	−0.2	431.10; 303.05; 297.10; 285.04; 151.00; 125.02; 107.02
**17**	quercetin 3-galactoside (isoquercetin)	7.99	C_21_H_20_O_12_	L2a	463.0873 [M − H]^−^	−0.9	343.05; 323.08; 301.04; 300.03; 271.02; 161.02; 151.00; 125.02; 107.01
**18**	3″,6″-di-*O*-*p*-coumaroyltrifolin	7.78	C_39_H_32_O_15_	L2a	739.1657 [M − H]^−^	−0.8	587.11; 569.11; 459.07; 133.09; 417.06; 339.04; 289.07; 245.08; 177.02; 161.02; 137.02; 125.02
**19**	kaempferide 3-rhamnoside	8.40	C_22_H_23_O_10_	L2a	447.1282 [M + H]^+^	−2.0	301.07; 286.05
*Flavonoids C-glycosides*							
**20**	isovitexin	7.88	C_21_H_20_O_10_	L2a	431.0980 [M − H]^−^	0.5	341.07; 323.06; 311.06; 283.06
*Biflavonoid*							
**21**	3‴-*O*-methylfukugetin	9.08	C_31_H_22_O_11_	L2a	571.1230 [M − H]^−^	1.8	553.26; 529.11; 377.06; 283.02; 123.04
*Procyanidin*							
**22**	procyanidin	6.43	C_30_H_26_O_12_	L2a	577.1333 [M − H]^−^	−2.3	451.10; 407.08; 339.08; 289.07; 161.02; 137.02; 125.02;
*Quinic acid derivatives*							
**23**	3-*O*-caffeoylshikimic acid	7.52	C_16_H_16_O_8_	L2a	335.0764 [M − H]^−^	−0.9	179.04; 173.05; 161.02; 135.04
**24**	3-*O*-*p*-coumaroylquinic acid	6.85	C_16_H_18_O_8_	L2a	337.0923 [M − H]^−^	0.0	191.06; 176.05; 163.04; 137.02; 119.05
**25**	chlorogenic acid	5.91	C_16_H_18_O_9_	L2a	353.0871 [M − H]^−^	−0.6	191.06; 173.05; 161.03; 135.04; 109.03
**26**	3-*O*-caffeoyl-4-*O*-methylquinic acid	7.62	C_17_H_20_O_9_	L2a	367.1027 [M − H]^−^	−0.5	191.05; 173.05; 161.02; 135.05
**27**	3,5-dicaffeoylquinic acid	8.97	C_25_H_24_O_12_	L2a	515.1183 [M − H]^−^	−1.4	353.09; 335.07; 191.06; 179.03; 173.05; 161.02; 135.04
*Coumarins*							
**28**	aesculin	5.44	C_15_H_16_O_9_	L2a	339.0721 [M − H]^−^	1.5	177.02; 149;02; 133.03; 105.04
**29**	phyllocoumarin	8.14	C_18_H_14_O_7_	L2a	341.0661 [M − H]^−^	0.0	323.06; 231.03; 203.03; 189.02; 189.02; 187.04; 151.04; 123.04; 109.03
**30**	fraxin	6.38	C_16_H_18_O_10_	L2a	393.0794 [M + Na]^+^	−1.0	231.03
**31**	fraxidin	8.38	C_11_H_10_O_5_	L2a	223.0599 [M + H]^+^	−3.1	208.04
**32**	naringenin-(3→8)-5,7-dihydroxychromone	10.12	C_24_H_16_O_9_	L2a	447.0717 [M − H]^−^	0.2	323.02; 295.03; 267.03; 151.04; 123.05
*Flavonolignans*							
**33**	cinchonain Ib	8.77	C_24_H_20_O_9_	L1	451.1030 [M − H]^−^	0.2	341.07; 323.06; 297.08; 289.07; 231.03; 217.01; 189.02; 177.02; 151.04
**34**	apocynin (A, B or C)	7.21	C_24_H_20_O_10_	L2a	467.0980 [M − H]^−^	0.4	357.06; 327.05; 305.07; 299.06; 231.03; 217.01; 189.02; 177.02; 139.04
**35**	cinchonain Ib derivative I	7.99	C_29_H_28_O_13_	L3	583.1444 [M − H]^−^	−1.4	451.10; 431.06; 341.07; 329.06; 299.06; 289.07; 161.03
**36**	cinchonain Ib derivative II	9.91	C_33_H_26_O_12_	L3	613.1349 [M − H]^−^	0.5	503.10; 461.09; 451.10; 393.06; 379.05; 351.05; 341.07; 323.06; 161.02
**37**	cinchonain Ib derivative III	9.39	C_48_H_38_O_18_	L3	901.1961 [M − H]^−^	−2.1	451.10; 417.06; 353.06; 341.07; 299.05; 287.06; 177.02; 161.02
**38**	cinchonain II	7.78	C_39_H_32_O_15_	L3	739.1650 [M − H]^−^	−1.8	569.11; 459.07; 417.06; 339.05; 289.07; 177.02; 161.02
*Triterpenes*							
**39**	11-oxooleanolic acid	20.71	C_30_H_46_O_4_	L2a	471.3459 [M + H]^−^	3.2	453.34; 425.34; 407.33; 341.24; 219.17; 159.12; 95.09
**40**	esterified triterpene with ferulic acid	21.76	C_40_H_56_O_7_	L3	647.3941 [M − H]^−^	−1.1	573.36; 465.26; 153.33; 193.05; 175.04; 149.06
**41**	esterified triterpene with *p*-coumaric acid	23.72	C_39_H_54_O_6_	L3	617.3826 [M − H]^−^	2.6	463.28; 161.02; 134.04
**42**	esterified triterpene with caffeic acid	21.19	C_39_H_54_O_7_	L3	633.3788 [M − H]^−^	0.5	589.39; 497.32; 479.28; 179.04; 161.02; 135.05
*Phenylpropanoid derivative*							
**43**	syringin	6.01	C_17_H_24_O_9_	L2a	395.1313 [M + Na]^+^	−1.3	232.07; 185.04
*Benzoic acid derivative*							
**44**	gaultherin	7.08	C_19_H_26_O_12_	L2a	469.1314 [M + Na]^+^	−1.7	317.08
*Primary metabolism*							
**45**	sucrose	0.77	C_12_H_22_O_11_	L2a	365.1055 [M + Na]^+^	−1.4	203.05; 185.04
**46**	pheophorbide A	24.08	C_35_H_36_N_4_O_5_	L2a	593.275 [M + H]^+^	−2.4	533.25
GC-MS							
*Fat acids*							
**47**	palmitic acid (methyl ester)	33.57	C_17_H_34_O_2_	L2a	270 [M^+ •]^	-	-
**48**	linoleic acid (methyl ester)	38.77	C_19_H_34_O_2_	L2a	294 [M^+ •]^	-	-
**49**	stearic acid (methyl ester)	39.86	C_19_H_38_O_2_	L2a	298 [M^+ •]^	-	-
**50**	oleic acid (methyl ester)	39.00	C_19_H_36_O_2_	L2a	296 [M^+ •^]	-	-

## Data Availability

The MS/MS data presented in this study are available in the Supporting Information.

## Supplementary Materials

The following are available online: Scheme S1.-Molecular networking fractions on negative polarity, Table S2. Collision energy, Fragmentation mechanism propose to dereplicated compounds, Mass spectrometry data (MS^1^ and MS^2^)-Figures S1–S100.
