# Supplementary material for: Chemophenetic Significance of Anomalocalyx uleanus Metabolites Are Revealed by Dereplication Using Molecular Networking Tools
Source: Molecules. 2021 Feb 9;26(4):925. doi: 10.3390/molecules26040925 (PMC7916253; doi:10.3390/molecules26040925)
Supplement: Supplementary file 1 [file molecules-26-00925-s001.pdf]

Supporting information

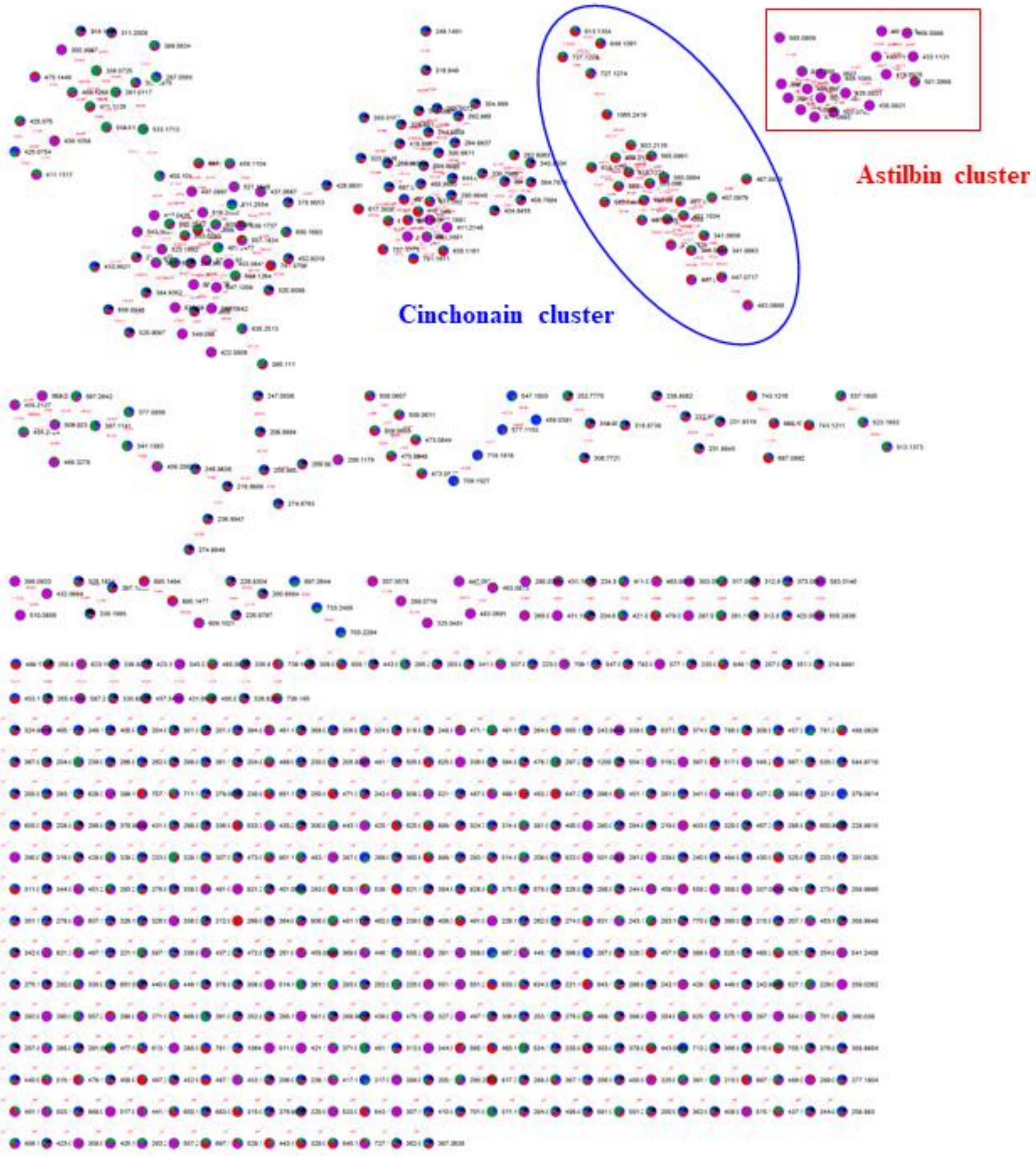

Scheme S1. - Molecular networking fractions on negative polarity.

Table S2. Collision energy.

Mass	Z1	Z2	Z3	Z4	Mass	Z1	Z2	Z3	Z4
100	30	28	25	25	100	30	28	25	25
300	35	32	30	30	300	35	32	30	30
500	40	38	35	35	500	40	38	35	35
700	50	48	45	45	700	50	48	45	45
1000	60	58	55	55	1000	60	58	55	55

Fragmentation mechanism propose to dereplicated compounds.

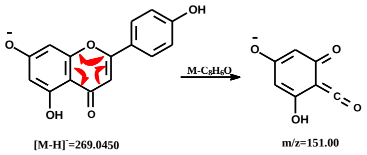

Apigenin **(1).**

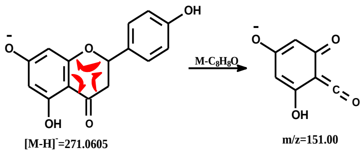

Narigenin **(2).**

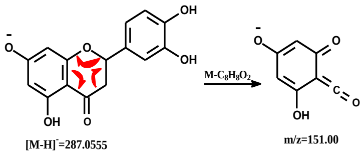

Eriocdictyol **(4).**

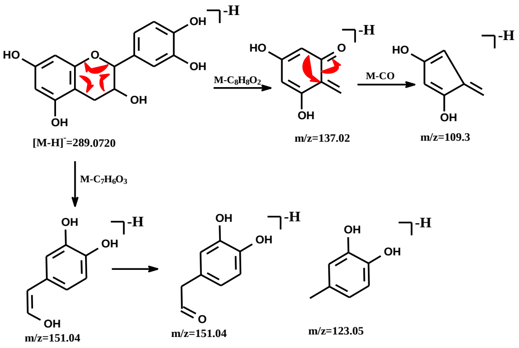

Catechin/epicatechin **(5a/5b).**

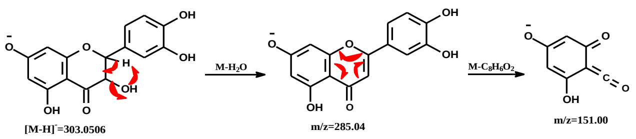

Taxifolin (**6**)

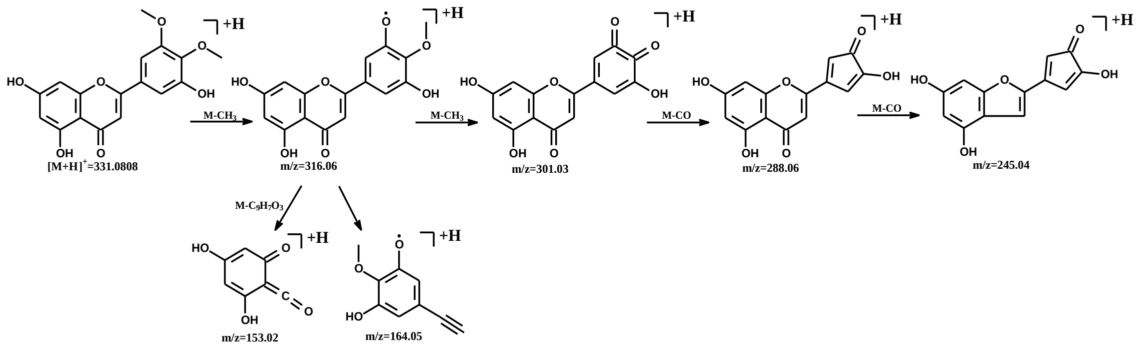

Apometzgerin **(7).**

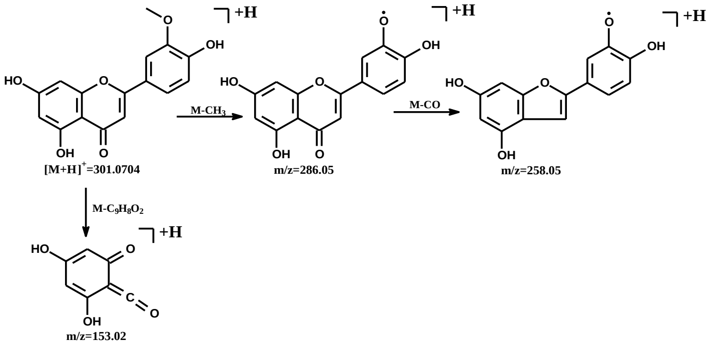

Chrysoeriol **(8).**

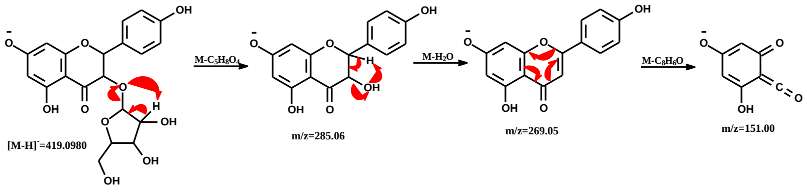

3-(arabinofuranosyloxy)-2,3-dihydro-5,7-dihydroxy-2-(4-hydroxyphenyl)-4H-1-benzopyran-4-one **(11).**

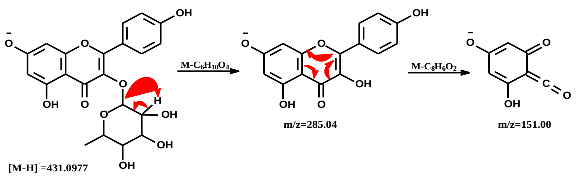

Afzelin **(12).**

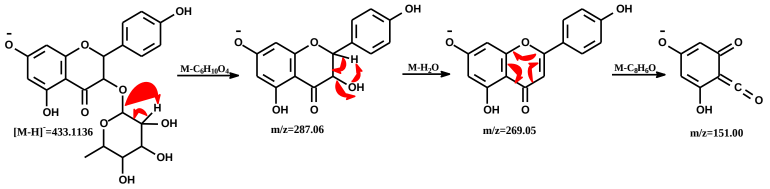

naringenin 3-O-glucoside (13).

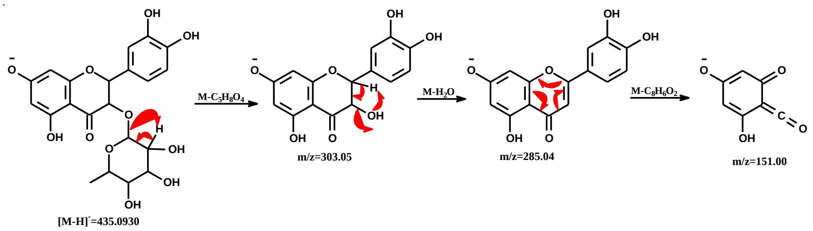

Taxifolin 3-*O*-xyloside (**14**)

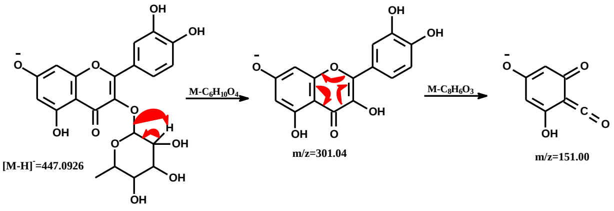

Quercetrin **(15).**

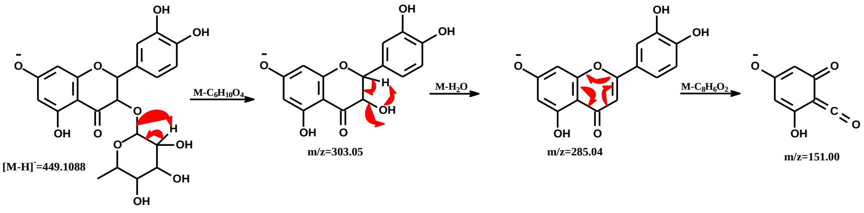

Astilbin **(16).**

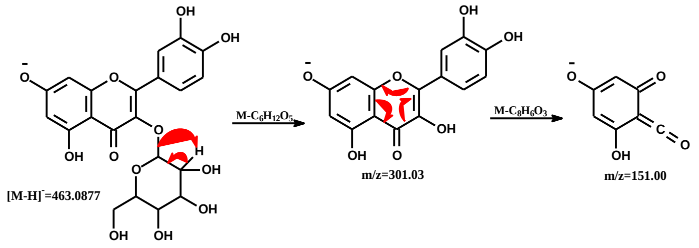

quercetin 3-galactoside (isoquercetrin) **(17).**

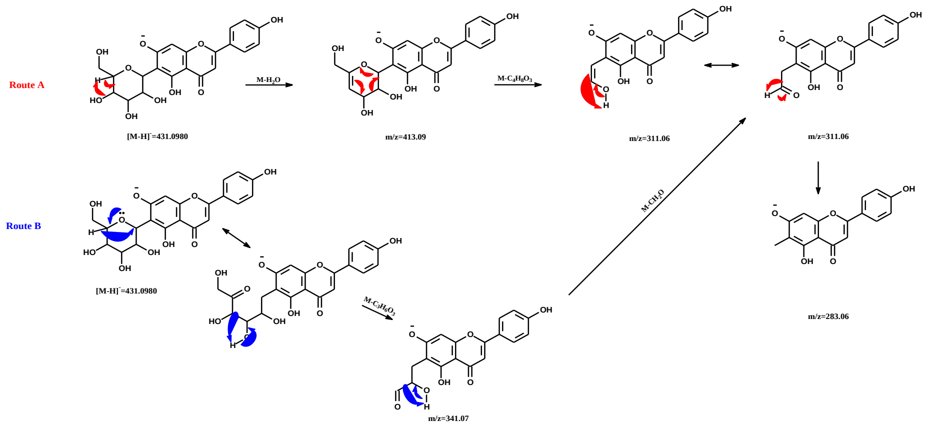

Isovitexin **(20).**

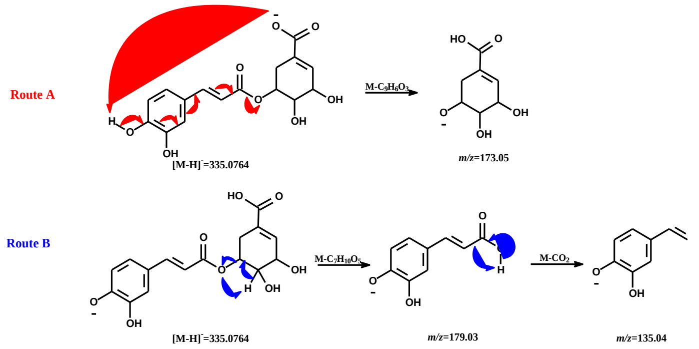

3-*O*-caffeoylshikimic acid **(23).**

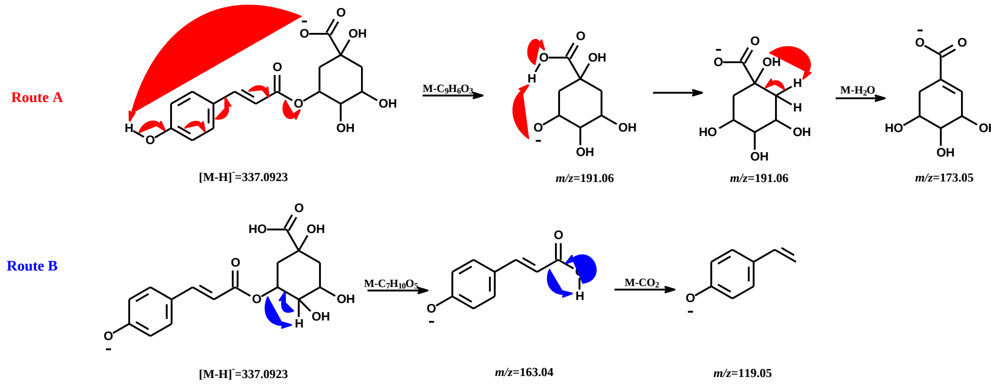

3-*O*-p-coumaroylquinic acid **(24).**

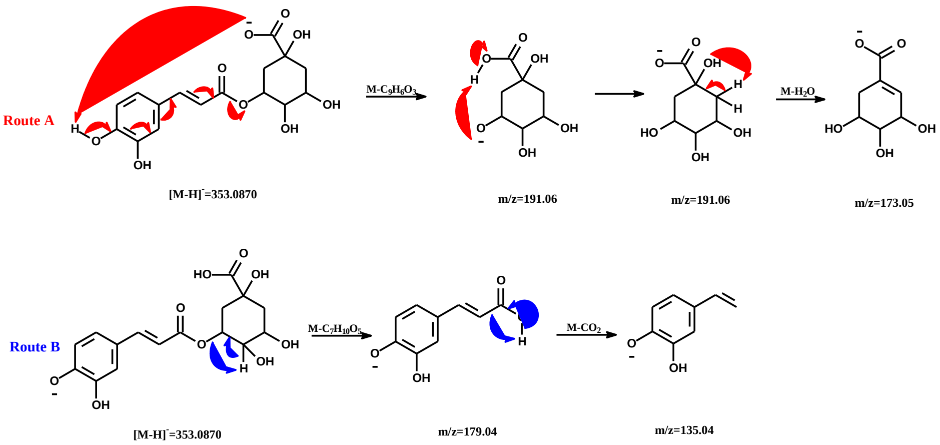

chlorogenic acid **(25).**

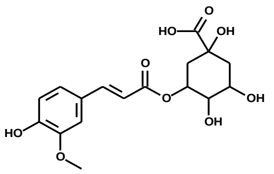

3,5-dicaffeoylquinic acid **(27).**

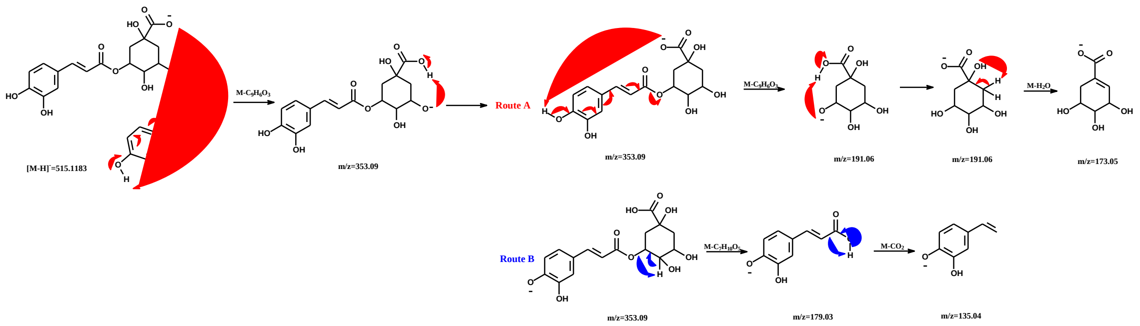

Aesculin **(28).**

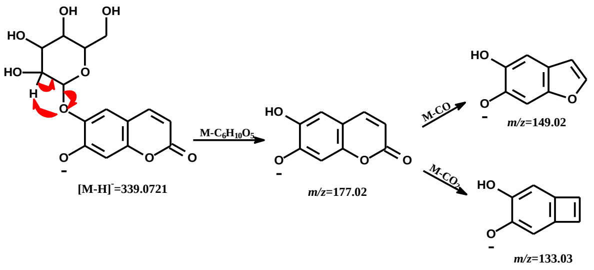

Phyllocoumarin **(29).**

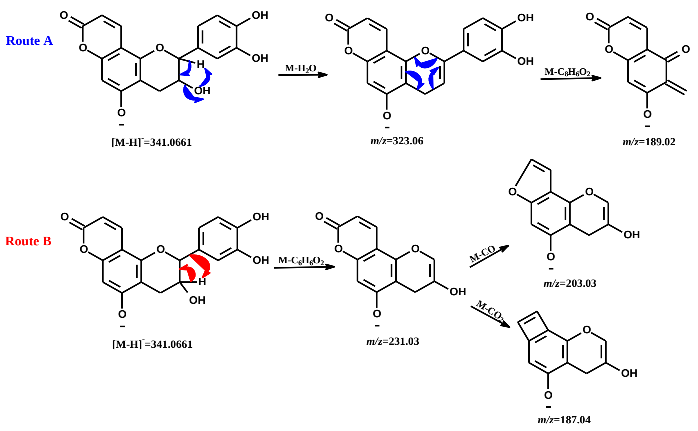

Cinchonain l **(33).**

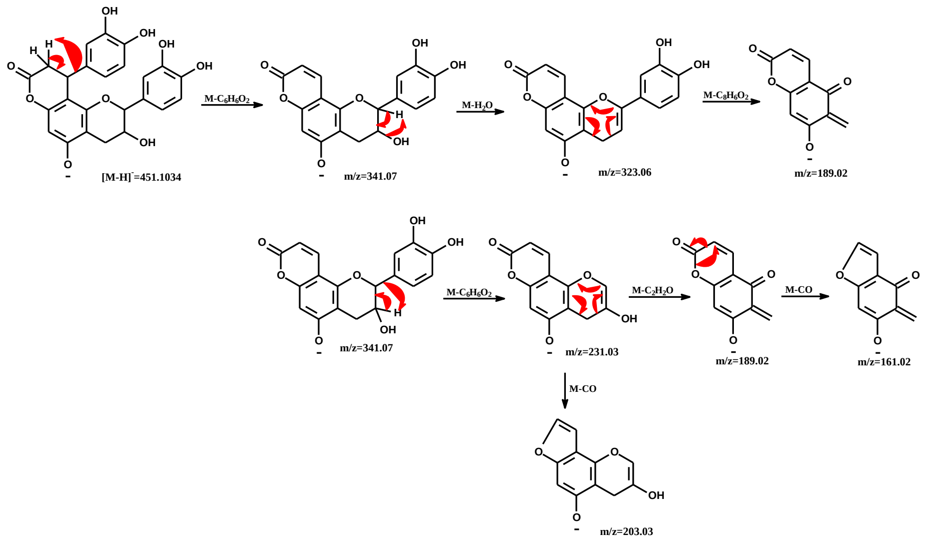

apocynin **(34).**

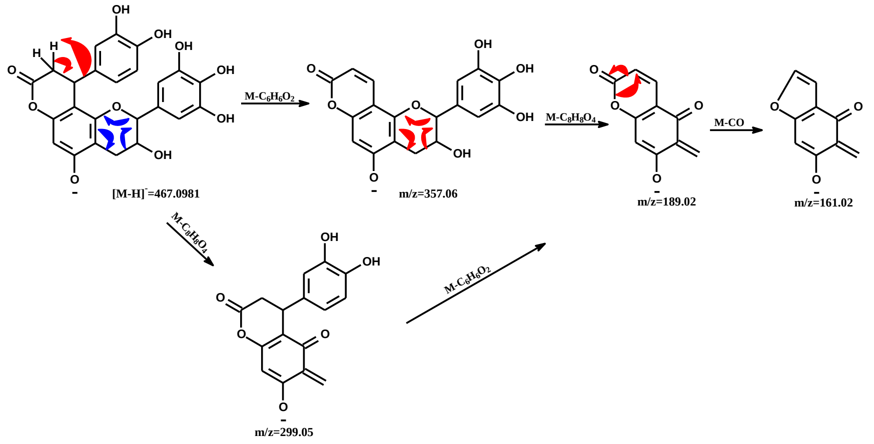

triterpene esterified with ferulic acid I (**40**)

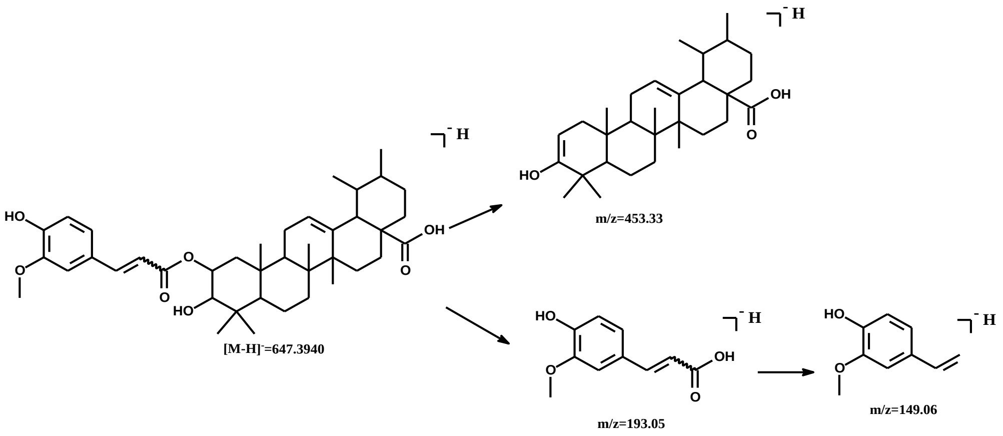

Supporting information of mass spectrometry data (MS[1] and MS[2])

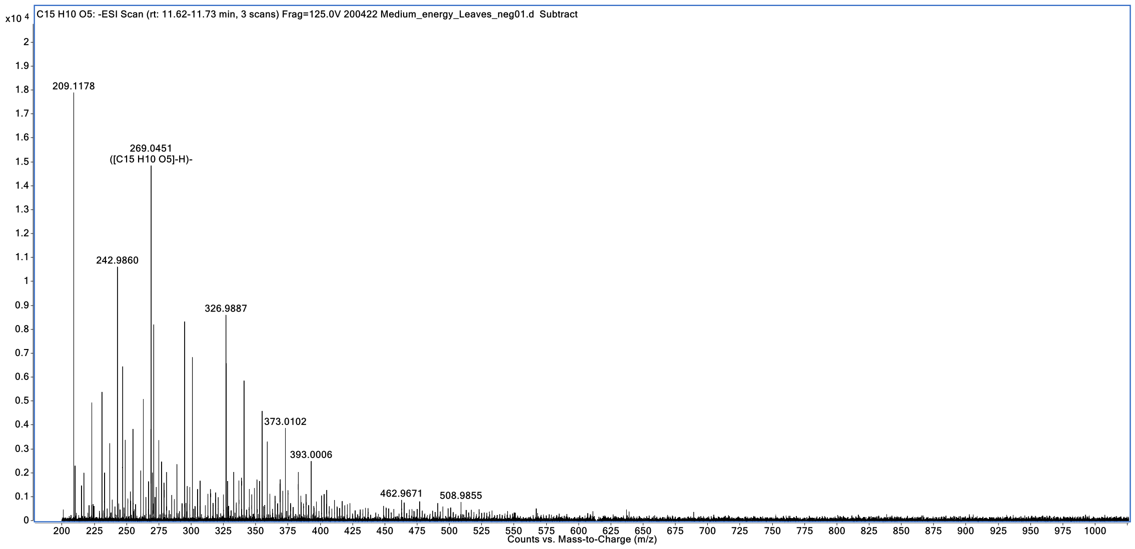

Figure S1. MS[1] spectra data from [M − H]⁻ = 269.0451, apigenin (**1**).

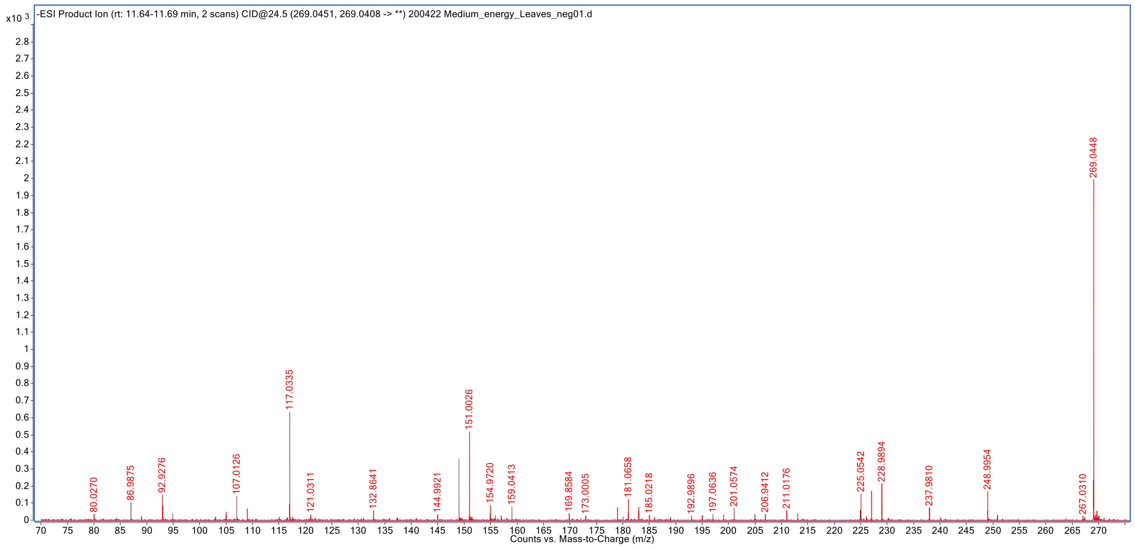

Figure S2. MS[2] spectra data from [M − H]⁻ = 269.0451, apigenin (**1**).

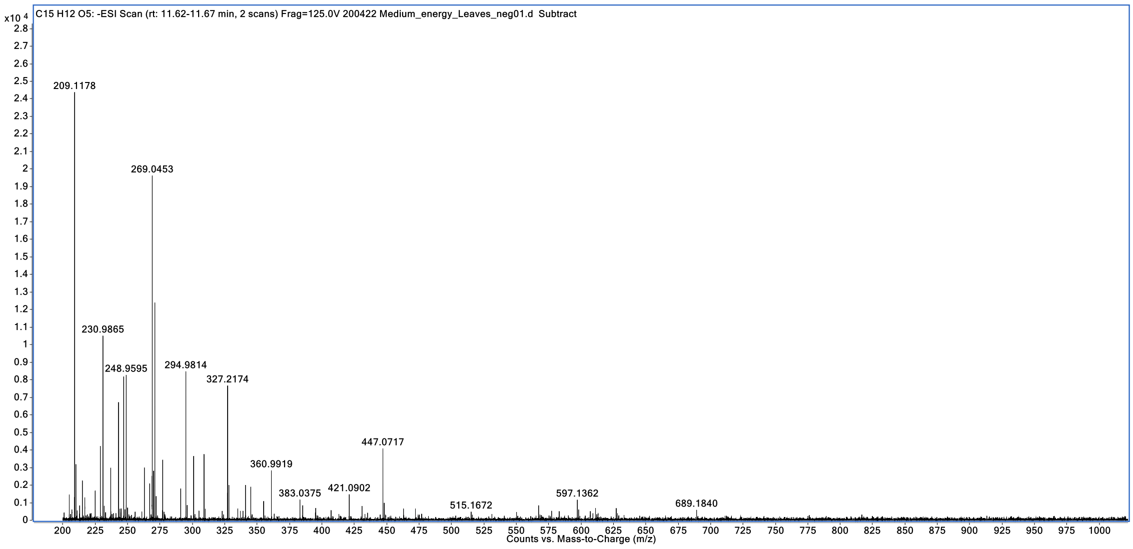

Figure S3. MS[1] spectra data from $[M-H]^-$ = 271.0610, naringenin (**2**).

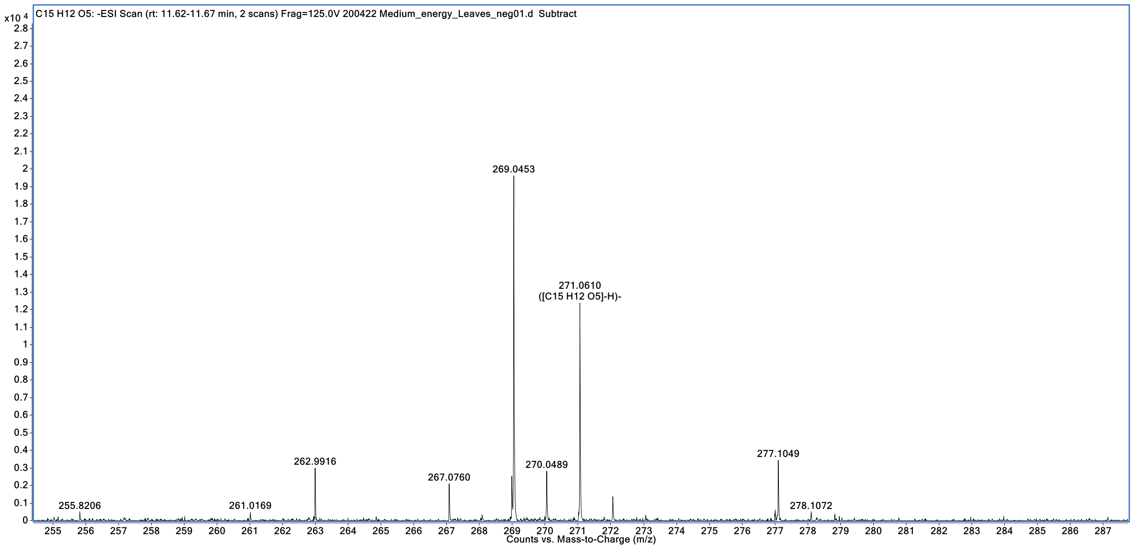

Figure S4. MS[1] expansion spectra data from $[M-H]^-$ = 271.0610 naringenin (**2**).

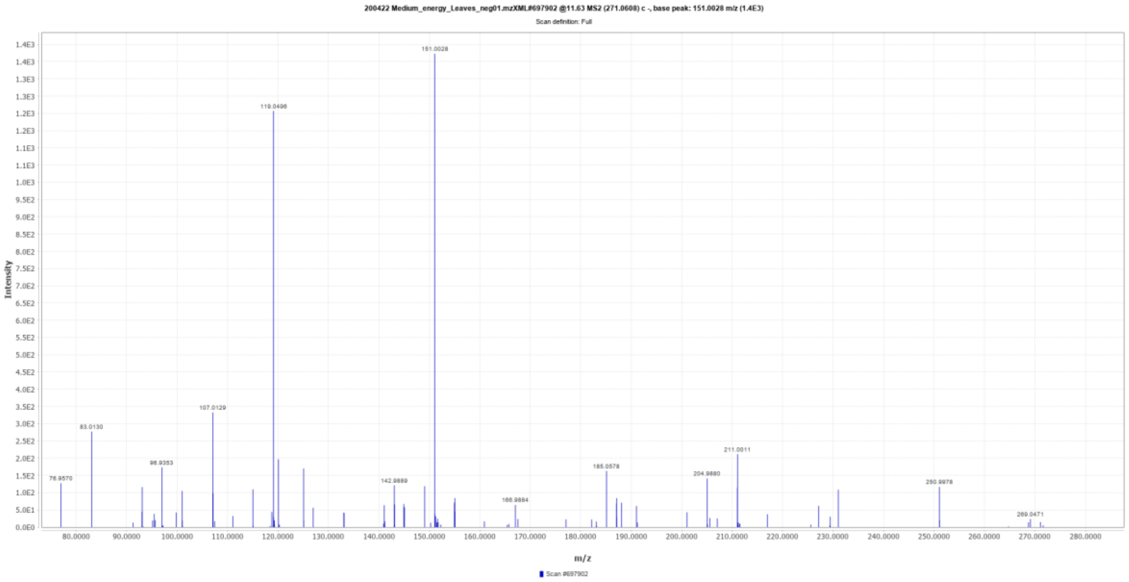

Figure S5. MS^2 spectra data from $[M-H]^-$ = 271.0610, naringenin (**2**).

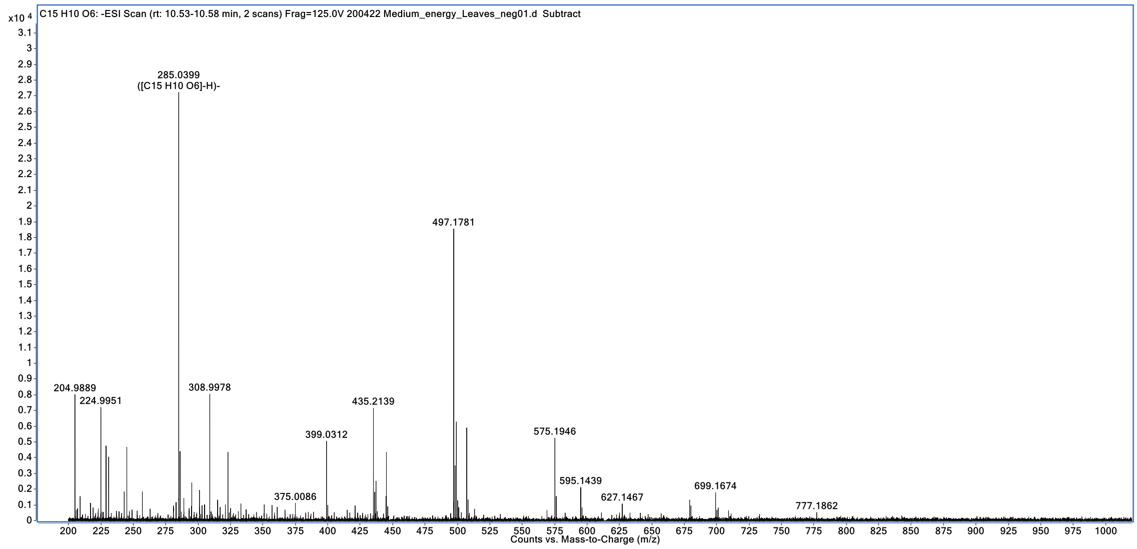

Figure S6. MS^1 spectra data from $[M-H]^-$ = 285.0399, kaempferol (**3**).

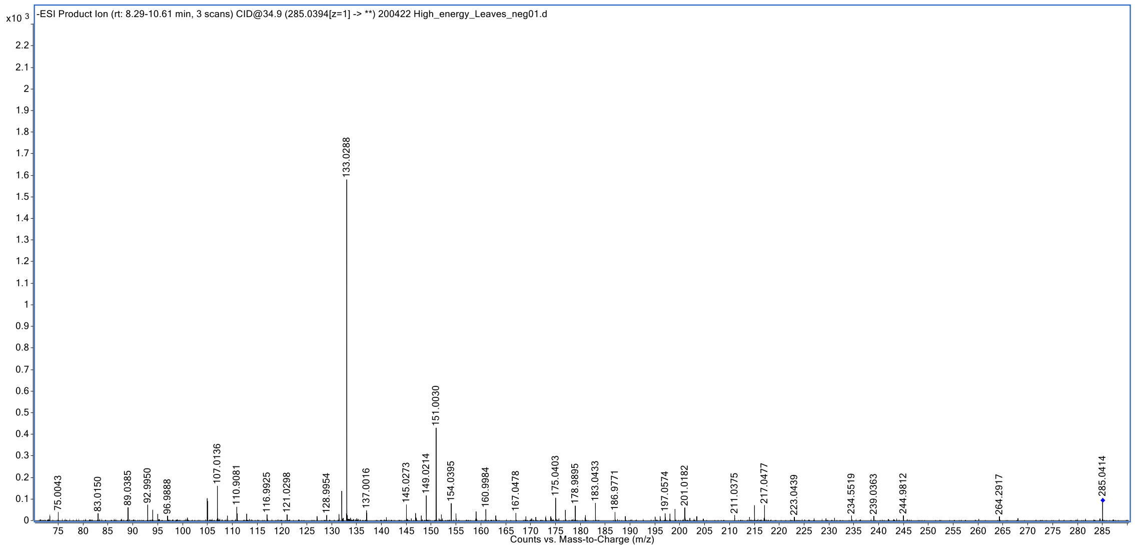

Figure S7. MS^2 spectra data from $[M-H]^-$ = 285.0399, kaempferol (**3**).

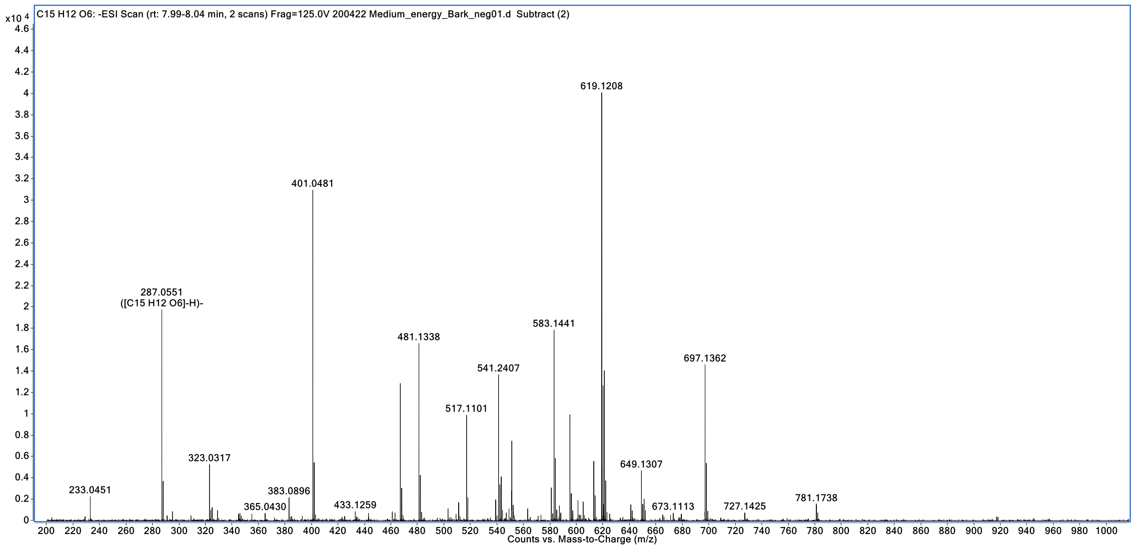

Figure S8. MS^1 spectra data from $[M-H]^-$ = 287.0551, eriodictyol (**4**).

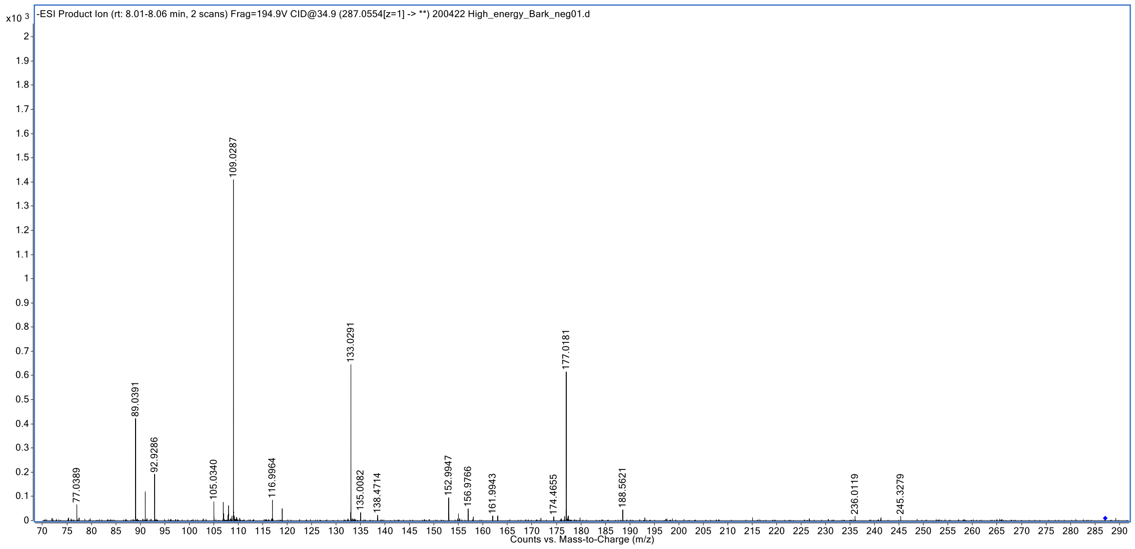

Figure S9. MS^2 spectra data from $[M - H]^-$ = 287.0551, eriodictyol (**4**).

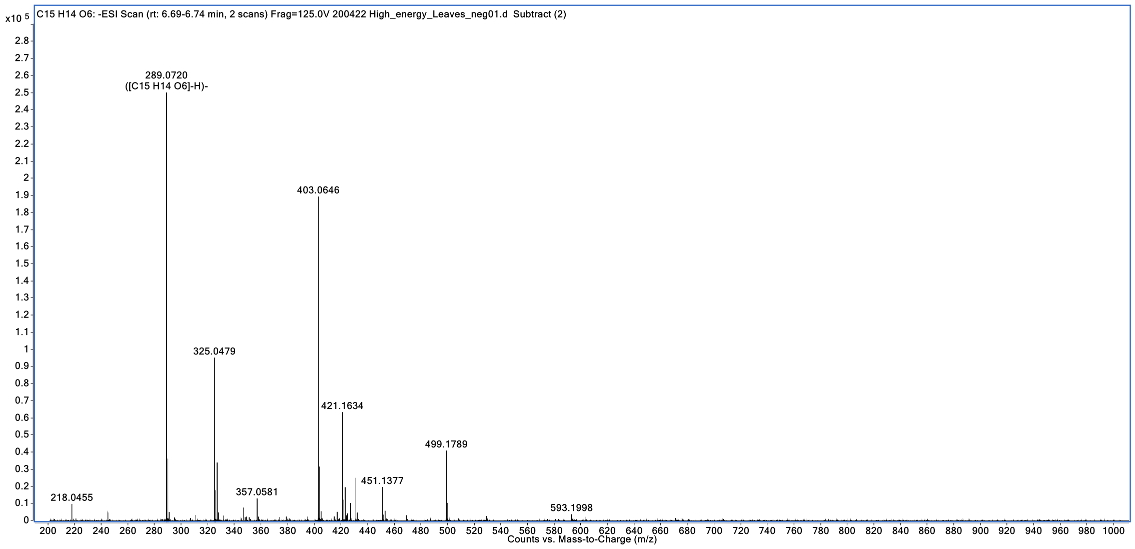

Figure S10. MS^1 spectra data from $[M - H]^-$ = 289.0720, catechin (**5a**)/epicatechin (**5b**).

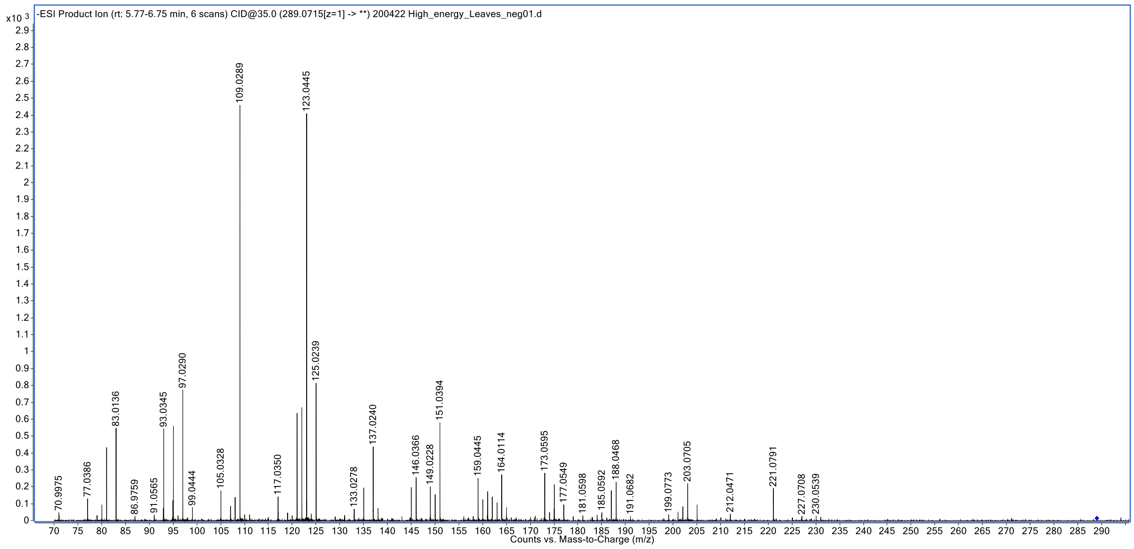

Figure S11. MS² spectra data from [M − H]⁻ = 289.0720, catechin (**5a**)/epicatechin (**5b**).

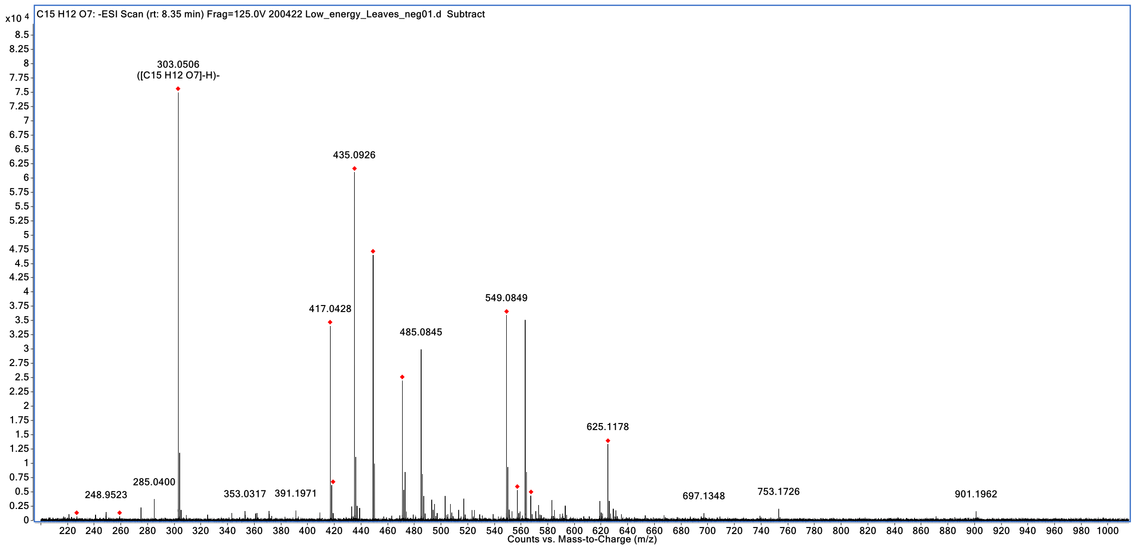

Figure S12. MS¹ spectra data from [M − H]⁻ = 303.0506, taxifolin (**6**).

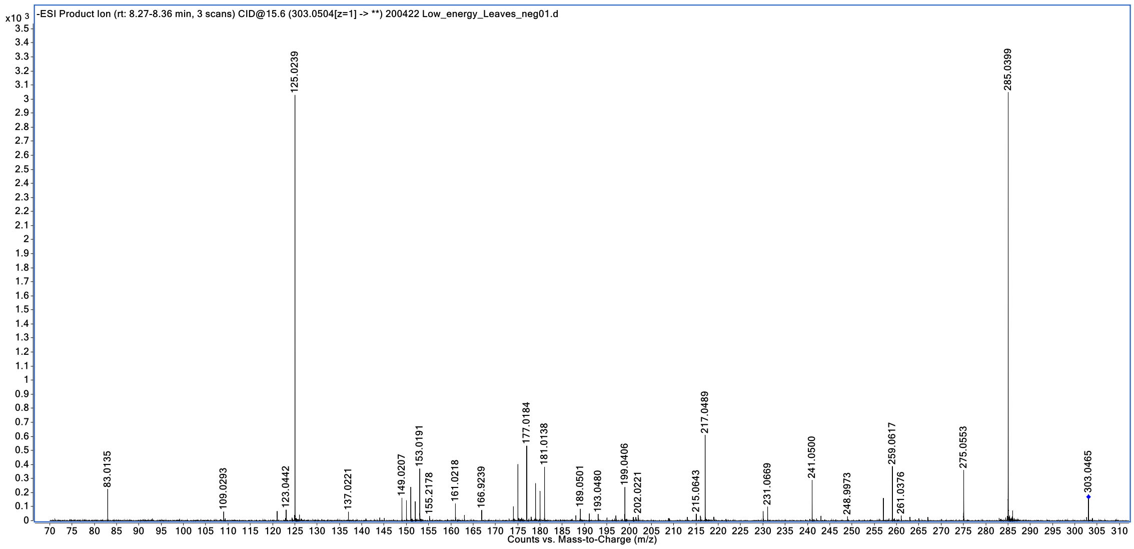

Figure S13. MS² spectra data from [M − H]⁻ = 303.0506, taxifolin (**6**).

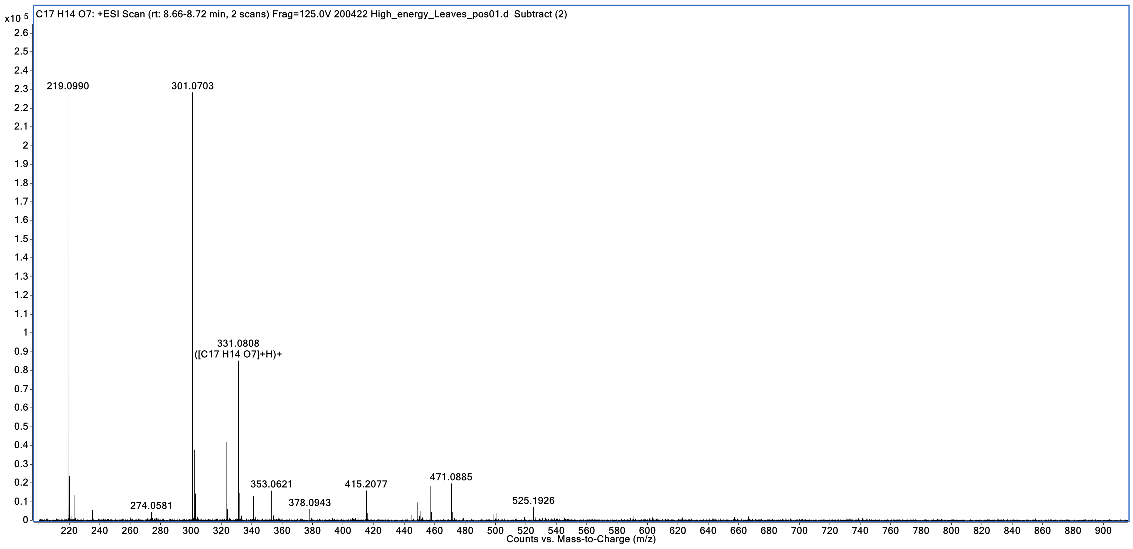

Figure S14. MS[1] spectra data from [M − H]⁻ = 331.0808, apometzgerin (**7**).

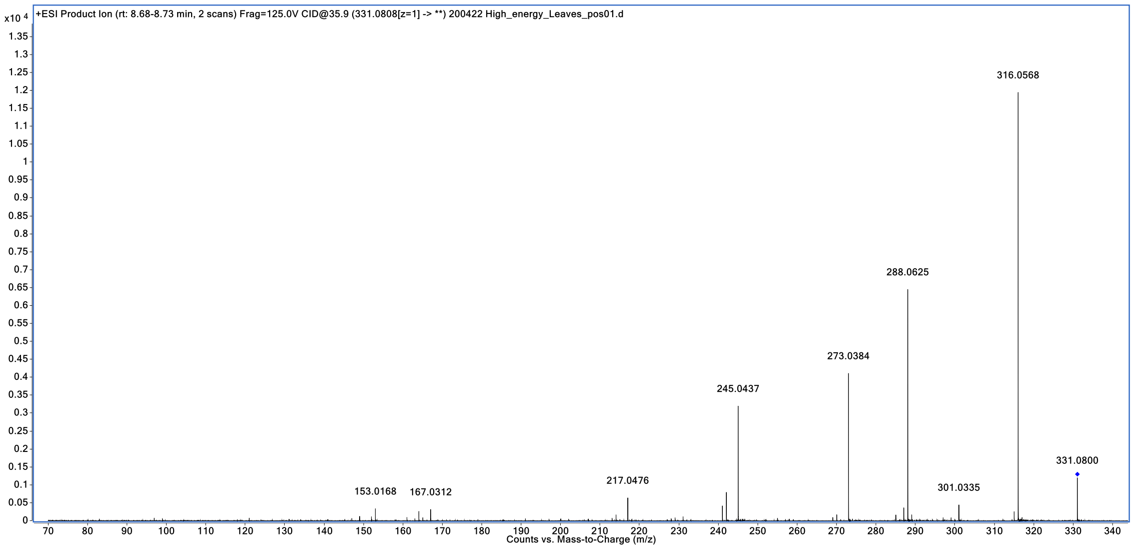

Figure S15. MS[2] spectra data from [M − H]⁻ = 331.0808, apometzgerin (7).

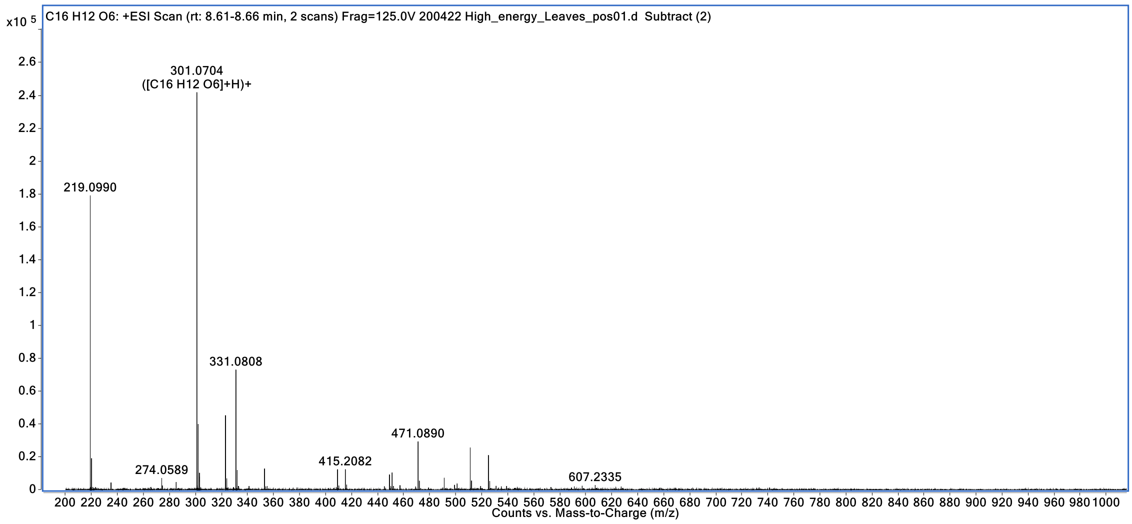

Figure S16. MS[1] spectra data from [M − H]⁻ = 301.0704, chrysoeriol (**8**).

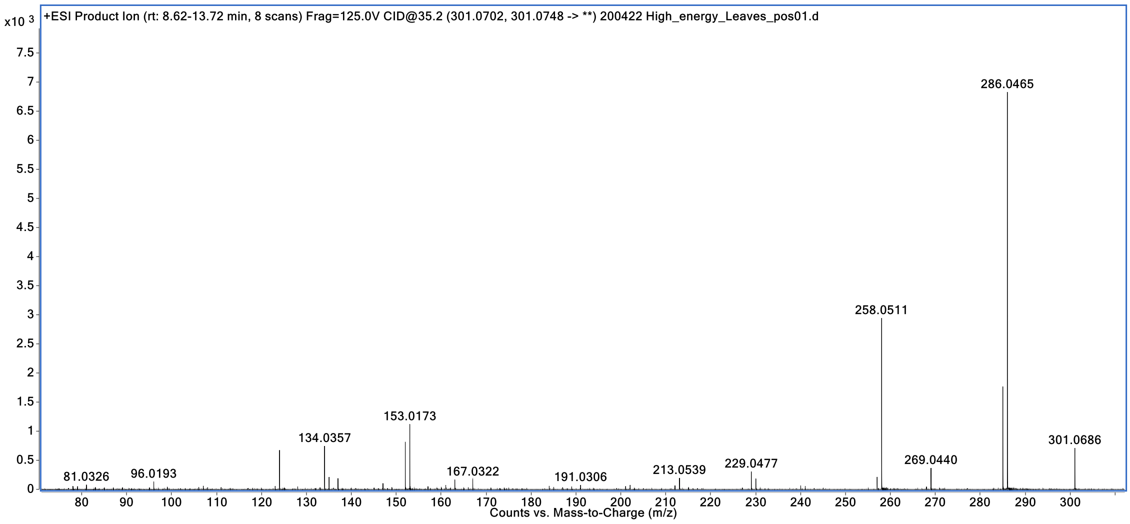

Figure S17. MS^2 spectra data from $[M-H]^-$ = 301.0704, chrysoeriol (**8**).

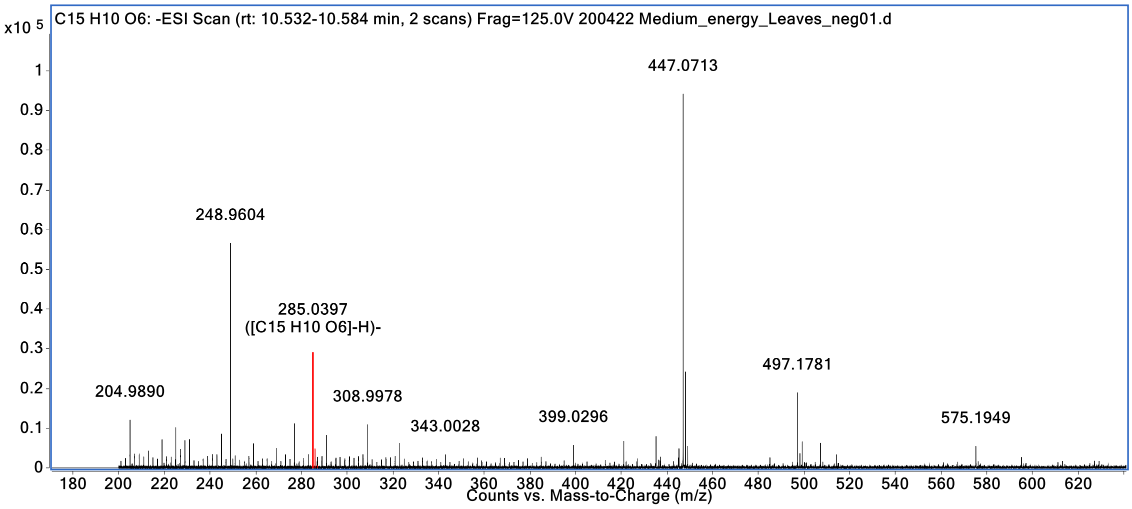

Figure S18. MS1 Spectra from $[M-H]^-$ = 285.0397, luteolin (**9**).

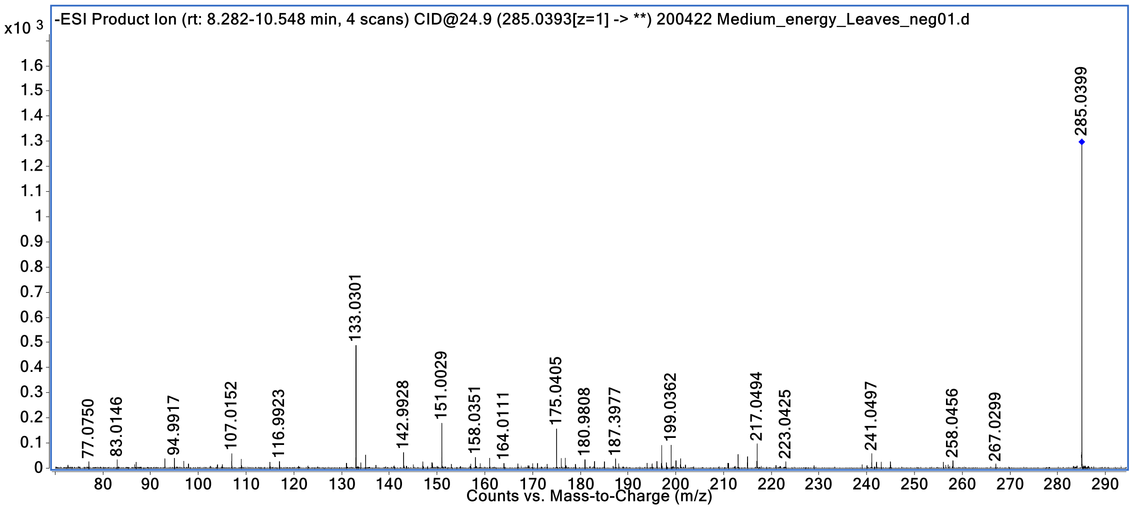

Figure S19. MS2 Spectra from $[M-H]^-$ = 285.0397, luteolin (**9**).

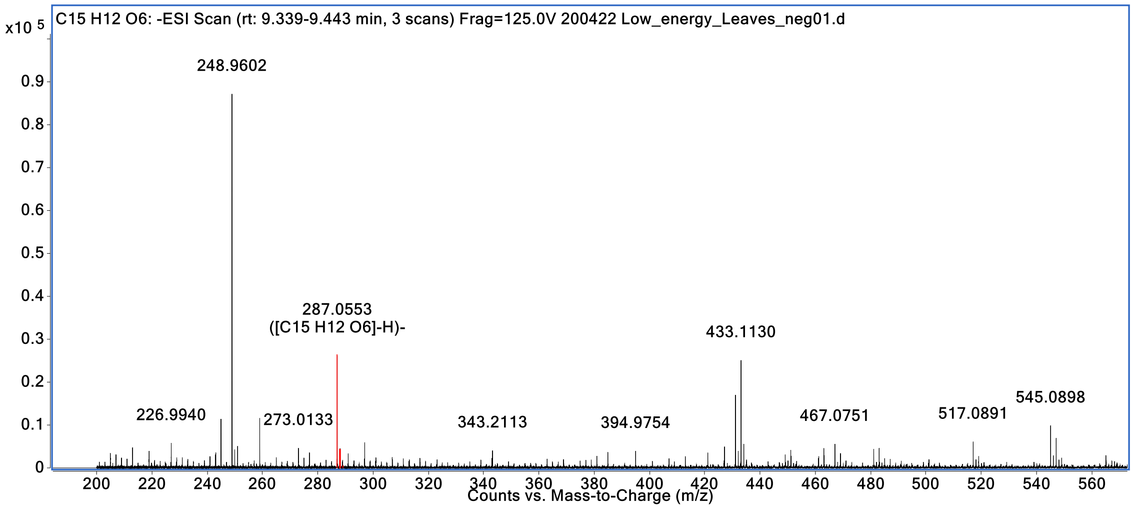

Figure S20. MS1 Spectra from [M − H]⁻ = 287.0553, aromadendrin (**10**).

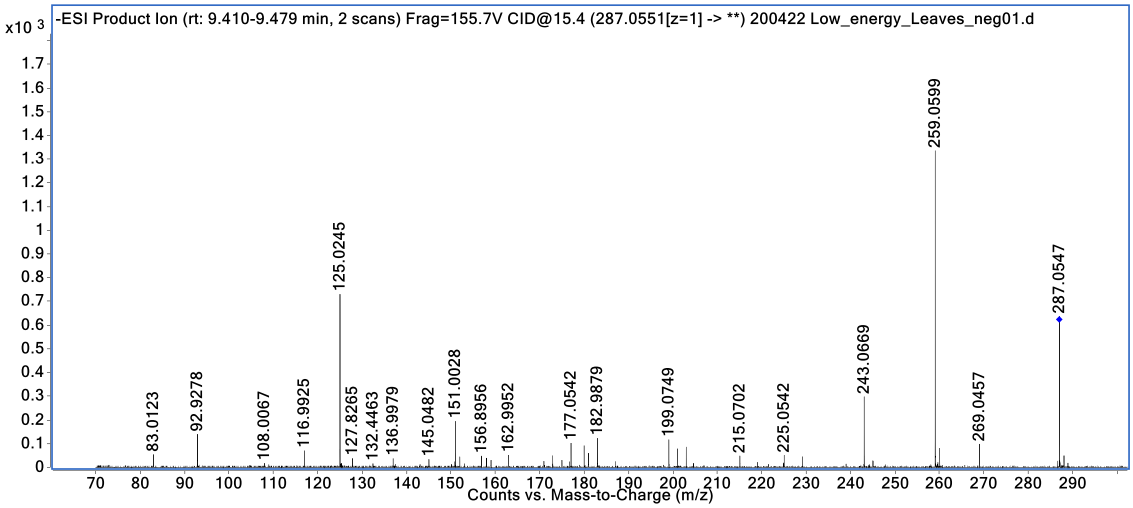

Figure S2 Spectra from [M − H]⁻ = 287.0553, aromadendrin (**10**).

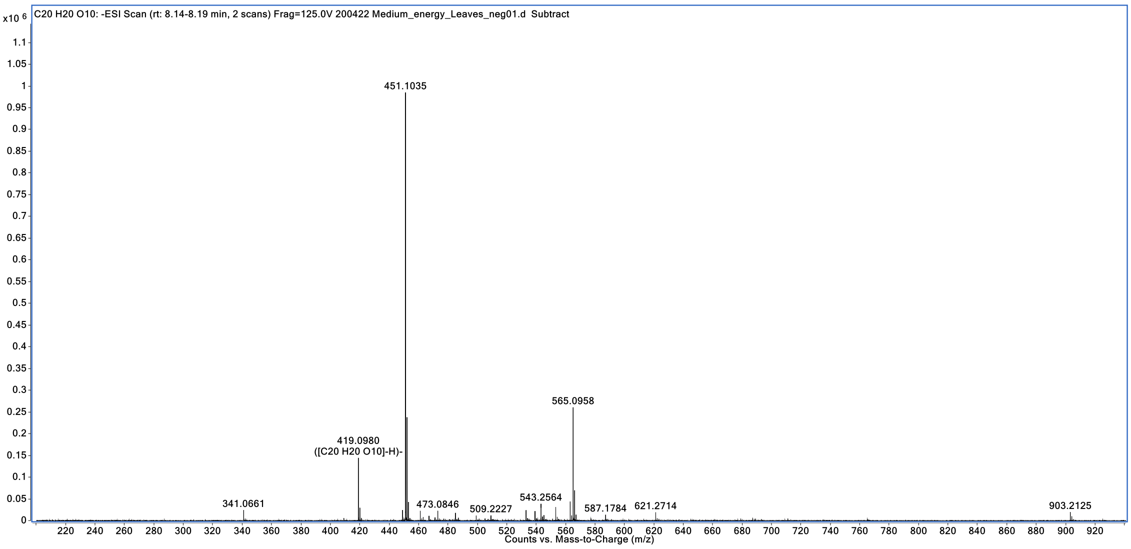

Figure S22. MS[1] spectra data from [M − H]⁻ = 419.0980, 3-(α-L-arabinofuranosyloxy)-2,3-dihydro-5,7-dihydroxy-2-(4-hydroxyphenyl)-4H-1-benzopyran-4-one (**11**).

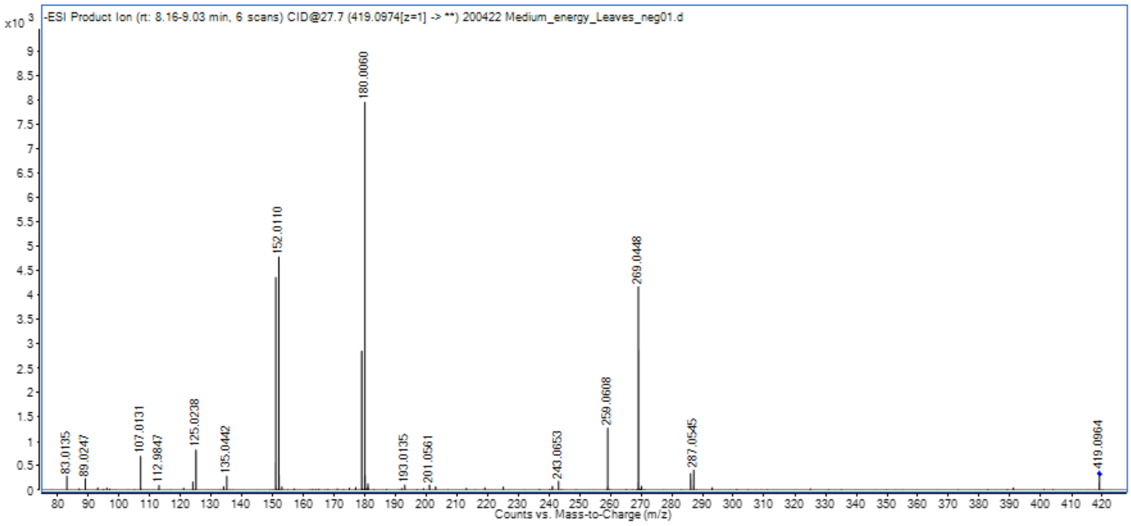

Figure S23. MS[2] spectra data from [M − H]⁻ = 419.0980, 3-(α-L-arabinofuranosyloxy)-2,3-dihydro-5,7-dihydroxy-2-(4-hydroxyphenyl)-4H-1-benzopyran-4-one (**11**).

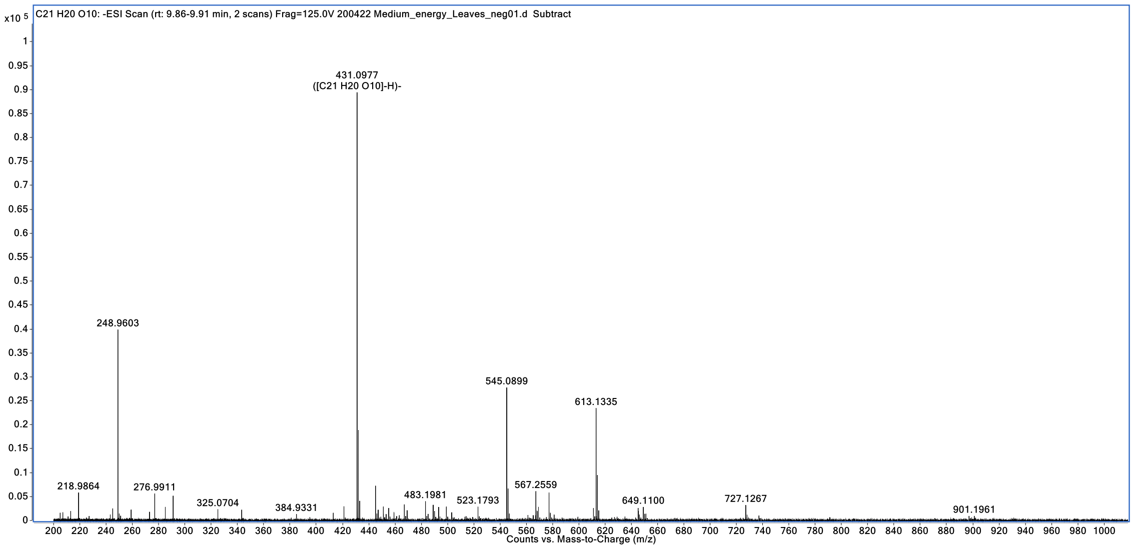

Figure S24. MS[1] spectra data from [M − H]⁻ = 431.0977, afzelin (**12**).

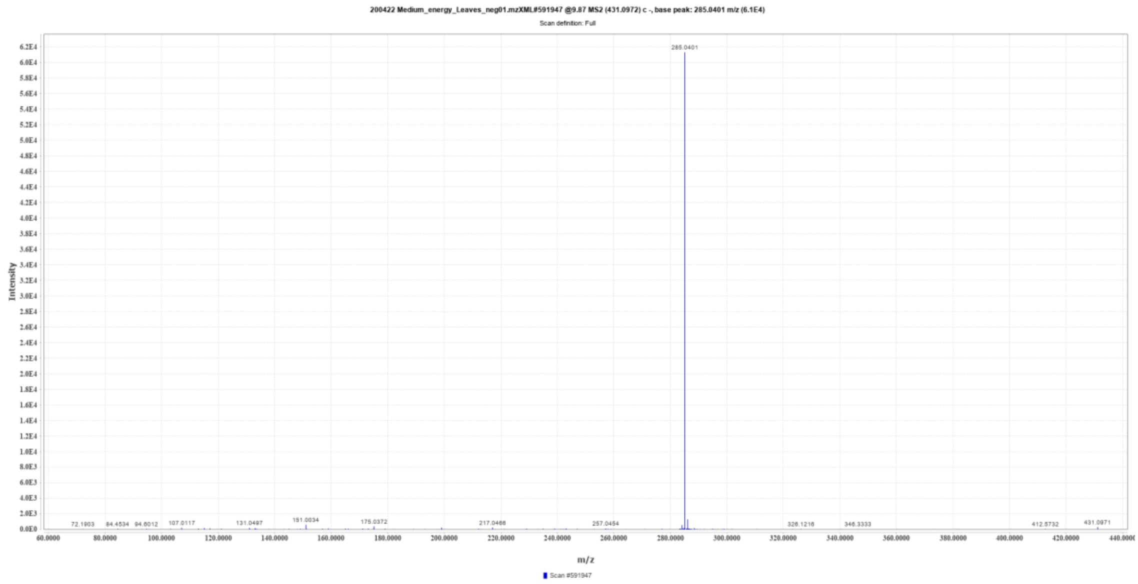

Figure S25. MS^2 spectra data from $[M-H]^-$ = 431.0977, afzelin (**12**).

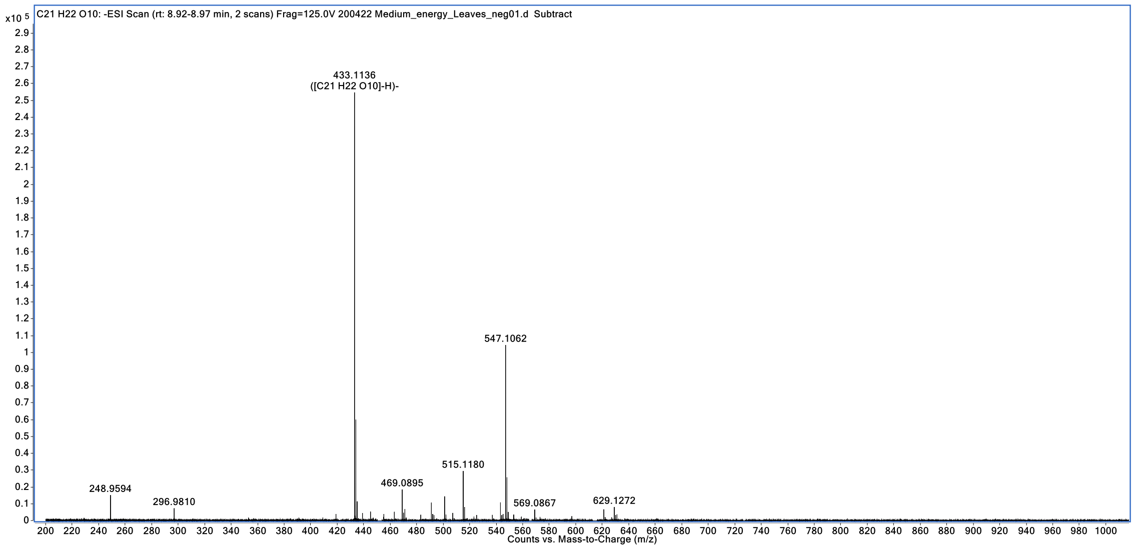

Figure S26. MS^1 spectra data from $[M-H]^-$ = 433.1136, naringenin 7-O-glucoside (hyperoside) (**13**).

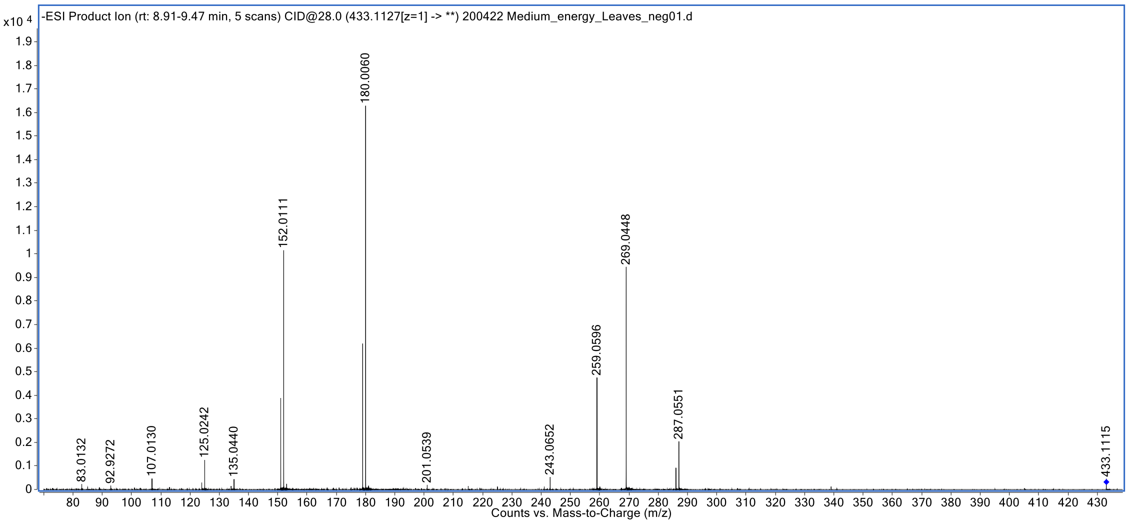

Figure S27. MS^2 spectra data from $[M-H]^-$ = 433.1136, naringenin 7-O-glucoside (hyperoside) (**13**).

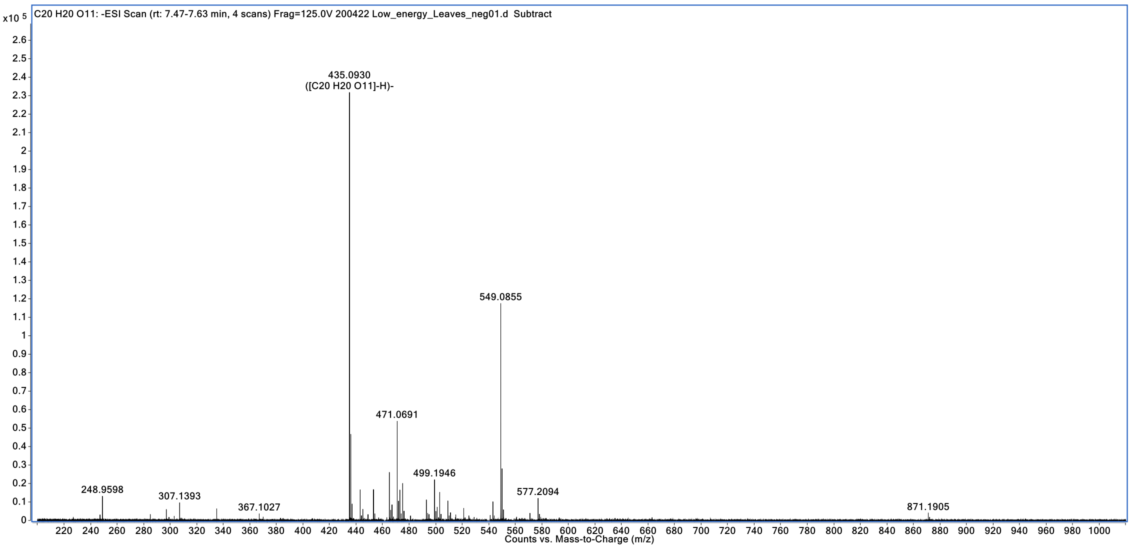

Figure S28. MS^1 spectra data from $[M-H]^-$ = 435.0930, taxifolin 3-xyloside (**14**).

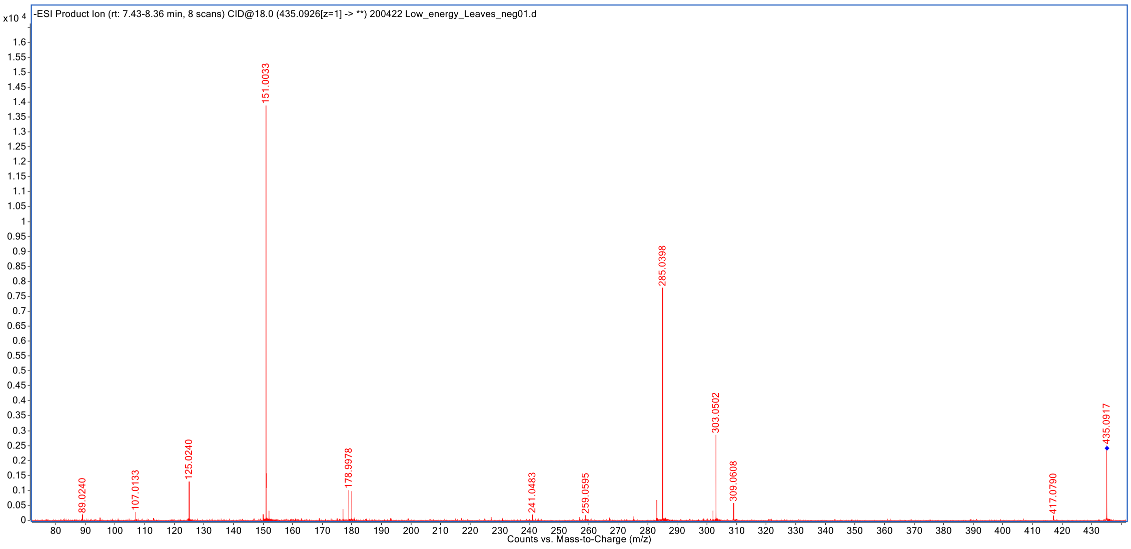

Figure S29. MS^2 spectra data from $[M-H]^-$ = 435.0930, taxifolin 3-xyloside (**14**).

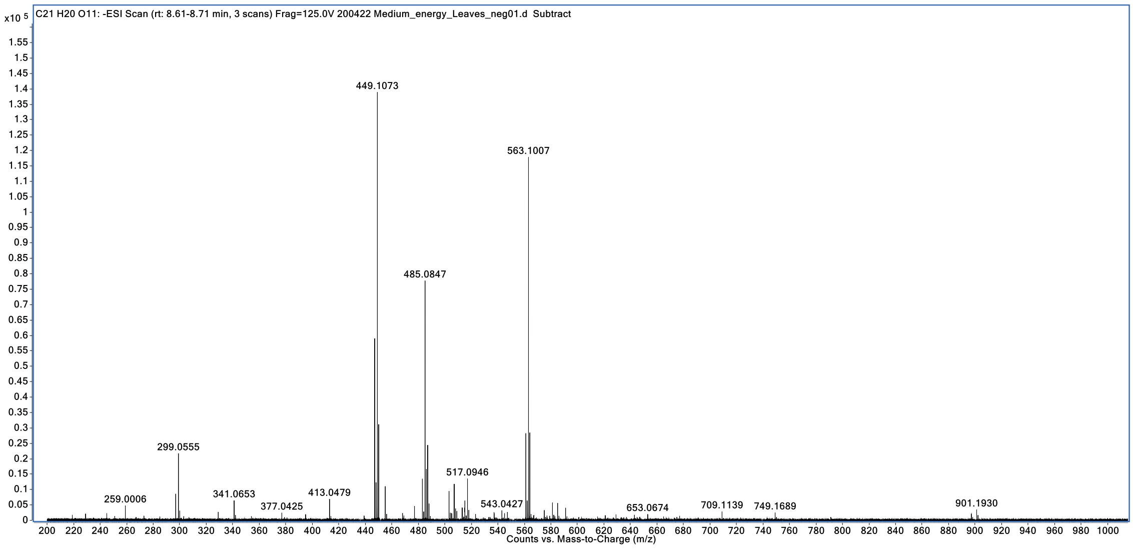

Figure S30. MS[1] spectra data from $[M-H]^-$ = 447.0926, quercetrin (**15**).

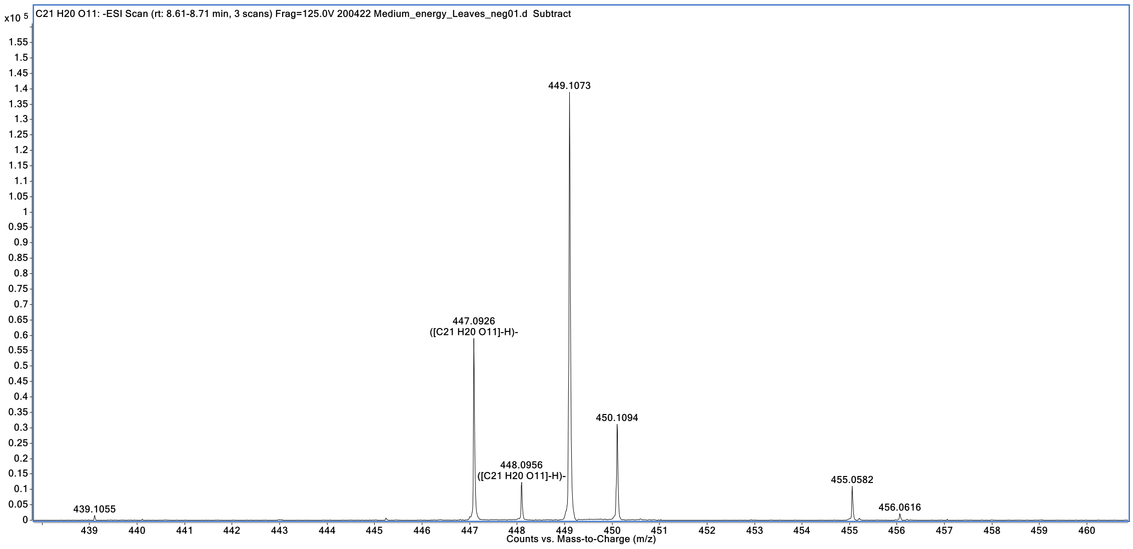

Figure S31. MS[1] expansion spectra data from $[M-H]^-$ = 447.0926, quercetrin (**15**).

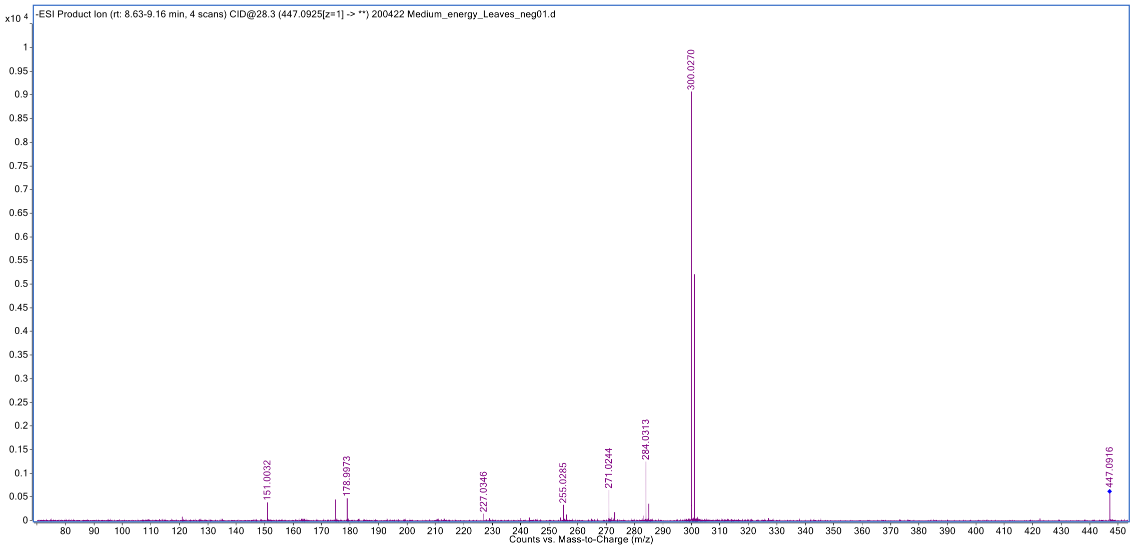

Figure S32. MS[2] spectra data from $[M-H]^-$ = 447.0926, quercetrin (**15**).

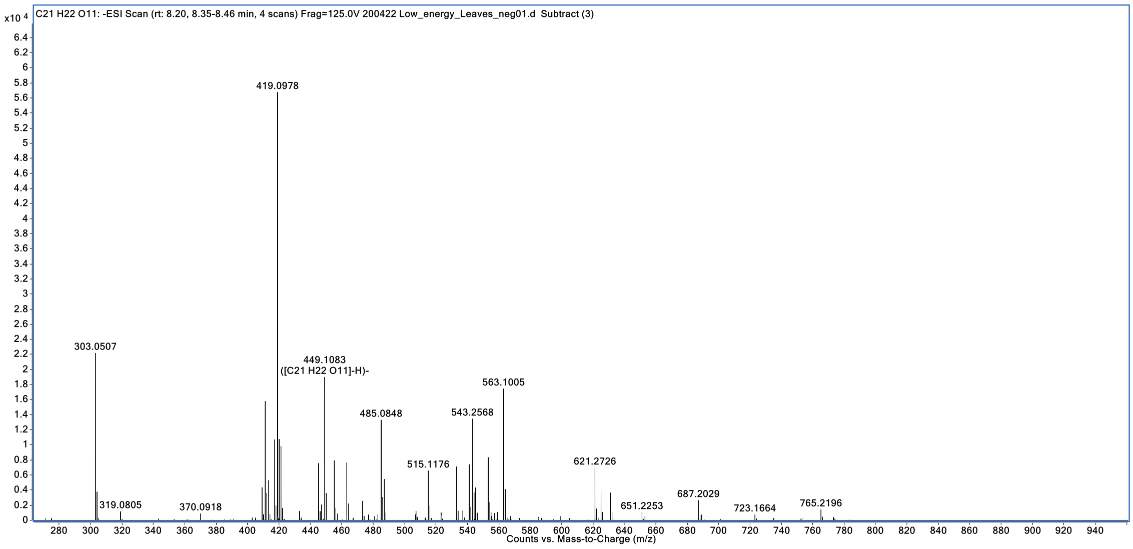

Figure S33. MS[1] spectra data from [M − H]$^-$ = 449.1083, astilbin (**16**).

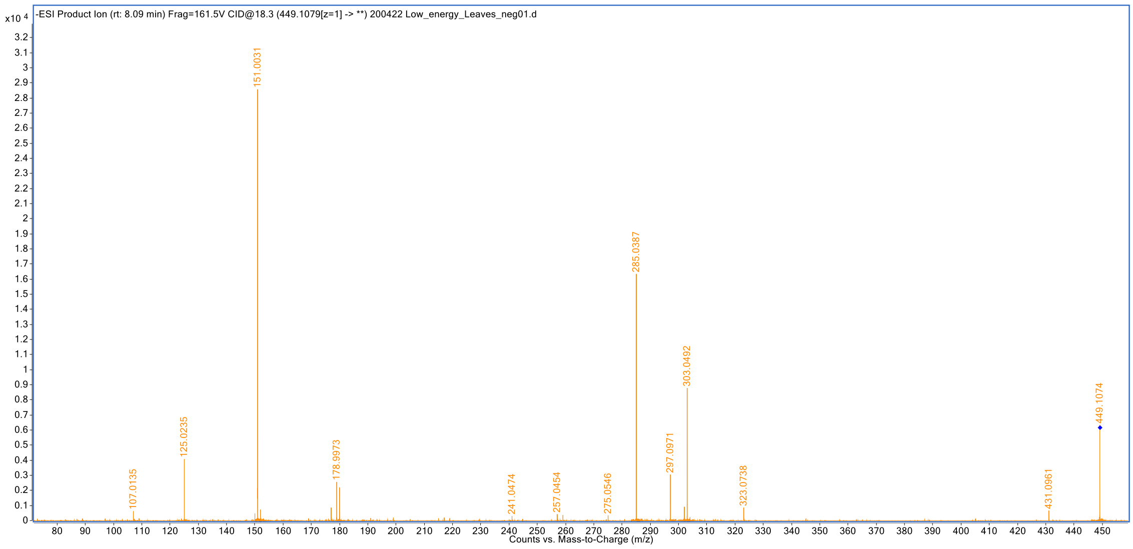

Figure S34. MS[2] spectra data from [M − H]$^-$ = 449.1083, astilbin (**16**).

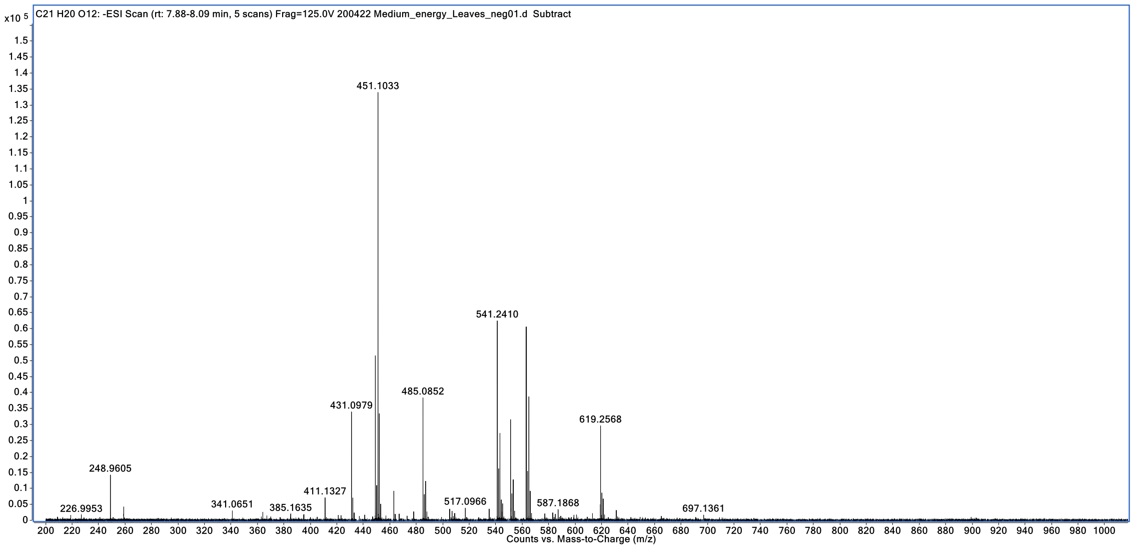

Figure S35. MS[1] spectra data from [M − H]$^-$ = 463.0873, quercetin 3-galactoside (isoquercetrin) (**17**).

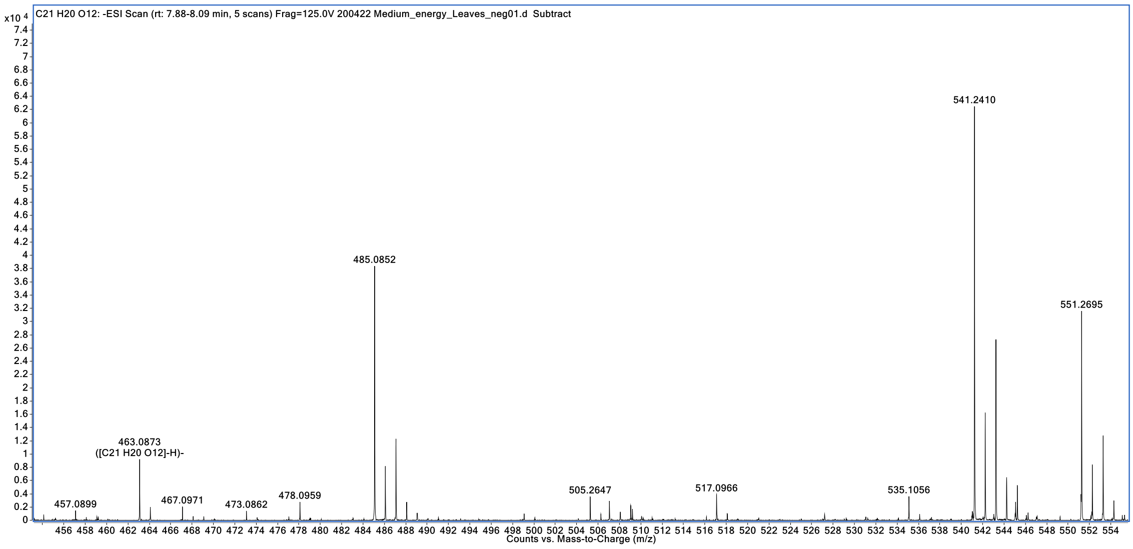

Figure S36. MS^1 expansion spectra data from $[M-H]^-$ = 463.0873, quercetin 3-galactoside (isoquercetrin) (**17**).

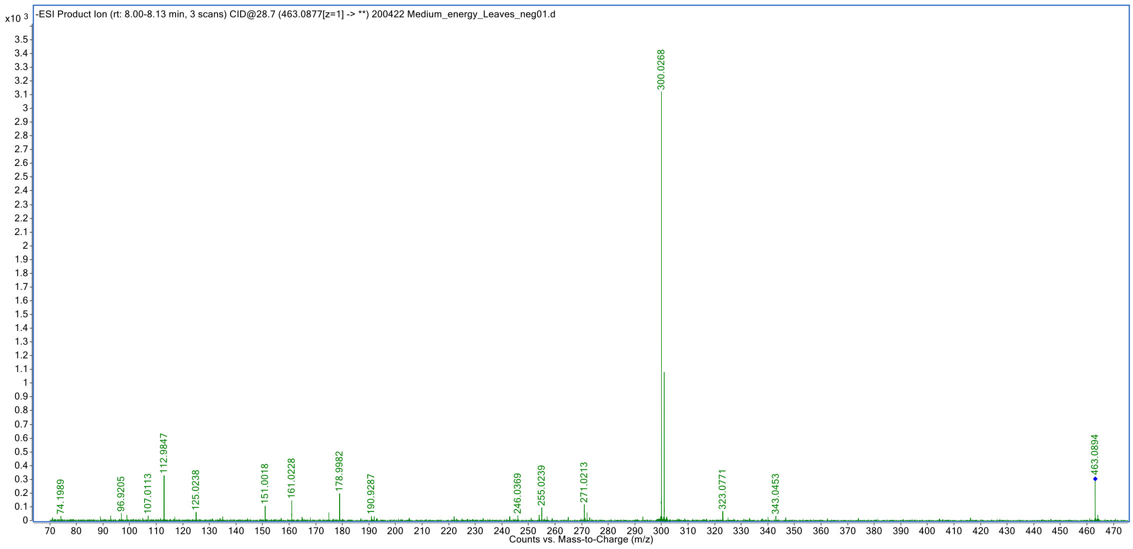

Figure S37. MS^2 spectra data from $[M-H]^-$ = 463.0873, quercetin 3-galactoside (isoquercetrin) (**17**).

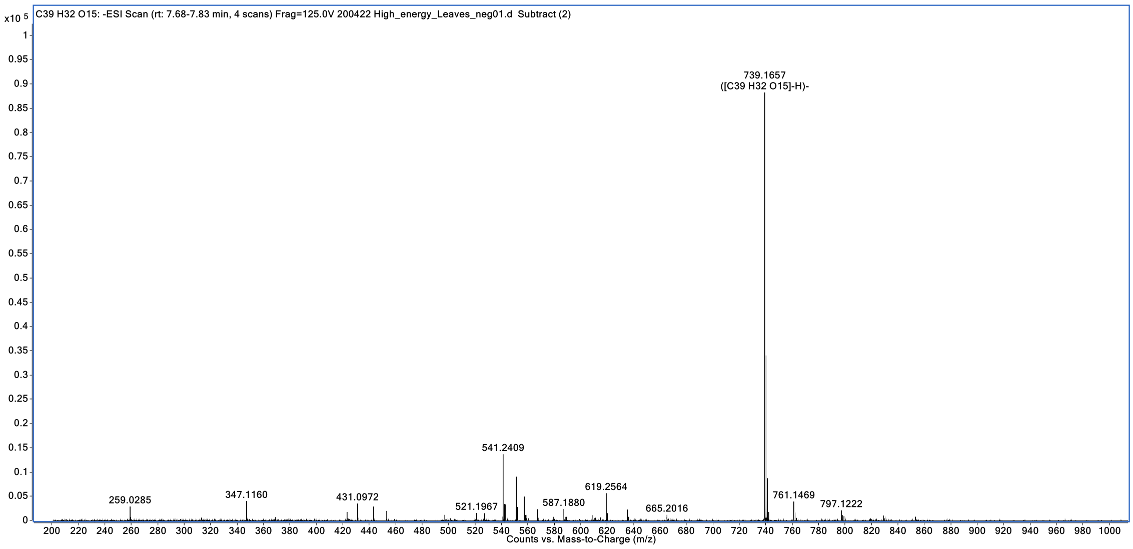

Figure S38. MS^1 spectra data from $[M-H]^-$ = 739.1657, 3'',6''-di-O-p-coumaroyltrifolin (**18**).

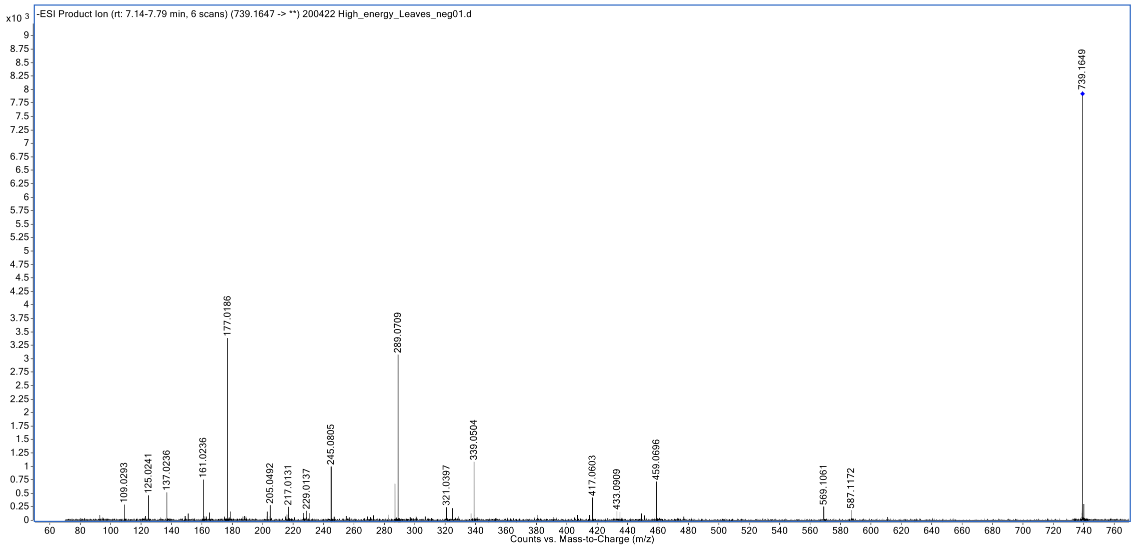

Figure S39. MS^2 spectra data from $[M-H]^-$ = 739.1657, 3'',6''-di-O-p-coumaroyltrifolin (**18**).

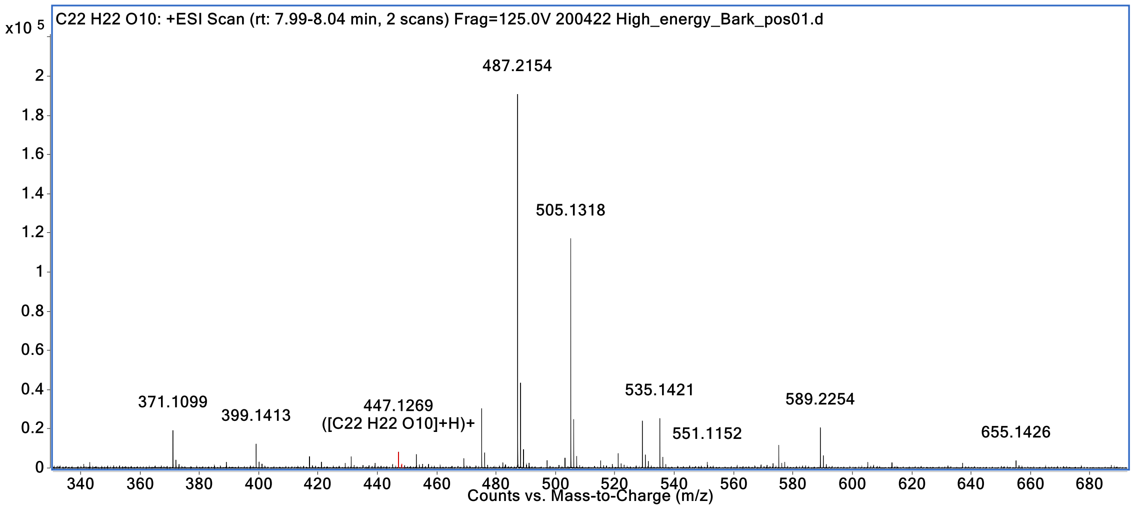

Figure 40. MS1 spectra data from [M+H]+=447.1269, kaempferide 3-rhamnoside (**19**).

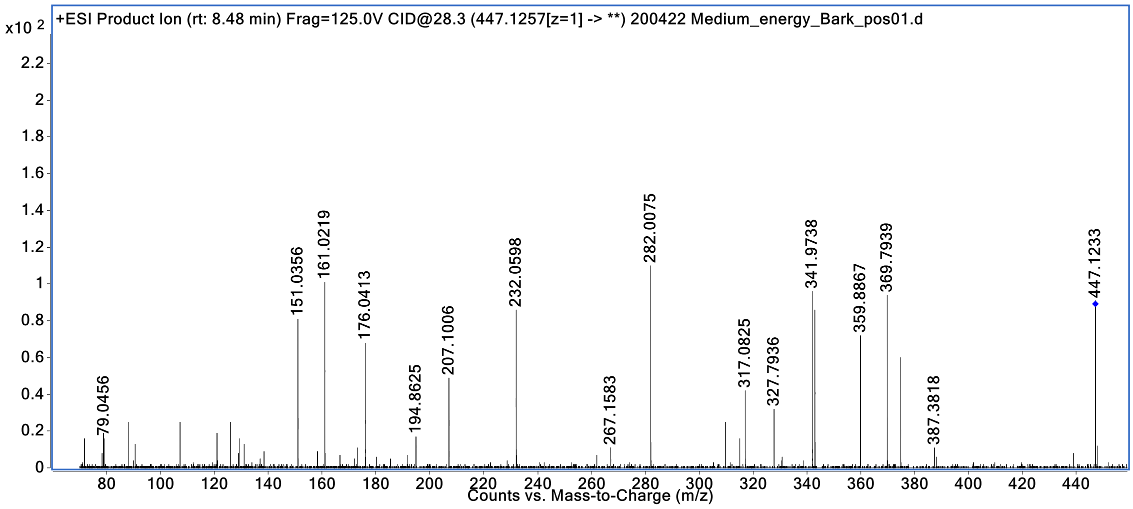

Figure 41. MS2 spectra data from [M+H]+=447.1269, kaempferide 3-rhamnoside (**19**).

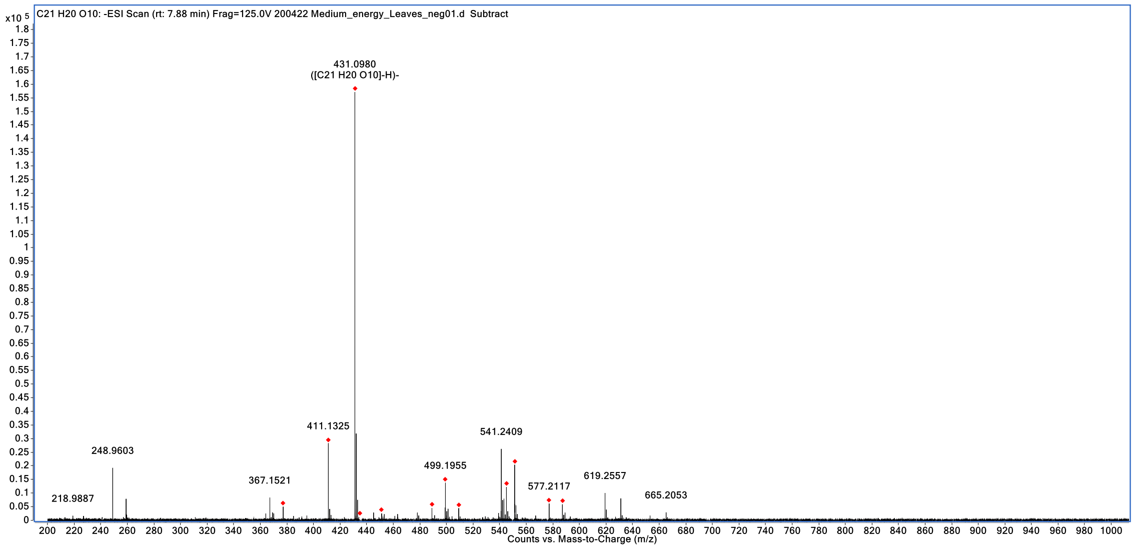

Figure 42. MS[1] spectra data from [M − H]− = 431.0980, isovitexin (**20**).

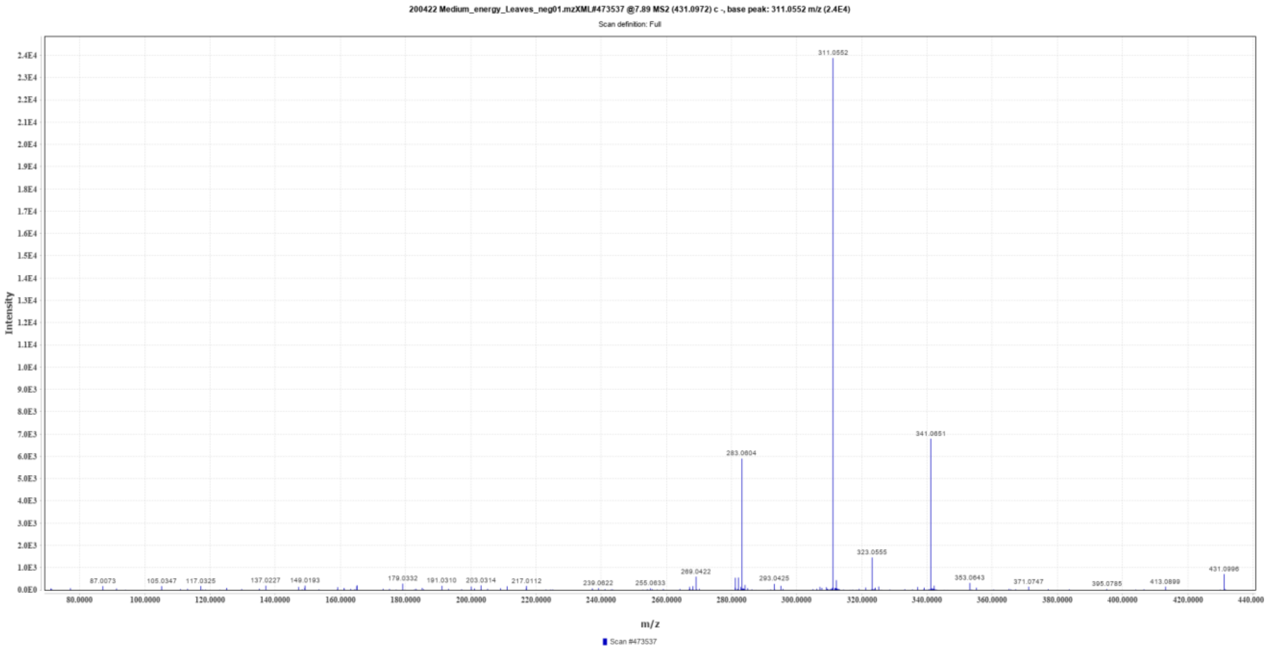

Figure 43. MS[2] spectra data from [M − H]− = 431.0980, isovitexin (**20**).

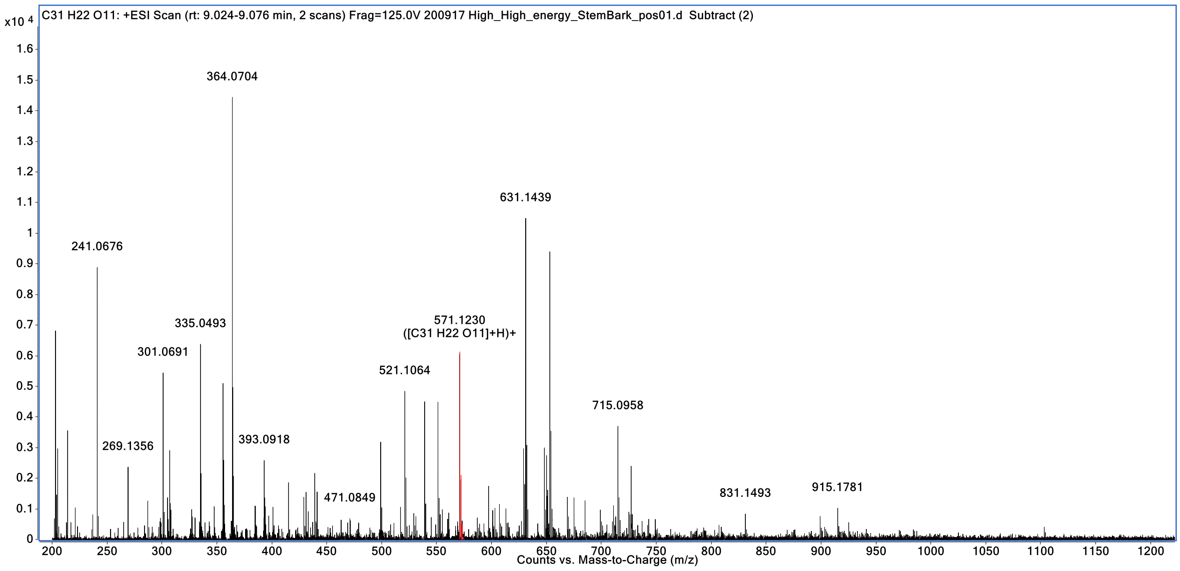

Figure 44. MS^1 spectra data from [M − H]− = 571.1230, 3'''-O-methylfukugetin (**21**).

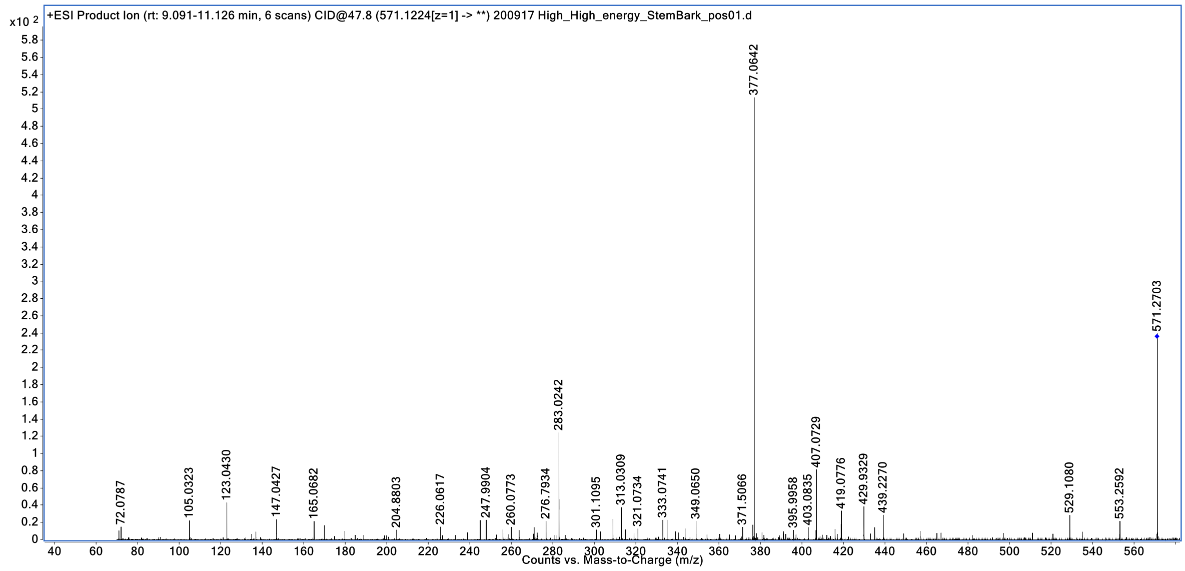

Figure 45. MS^2 spectra data from [M-H]$^-$=571.1230, 3'''-O-methylfukugetin (**21**).

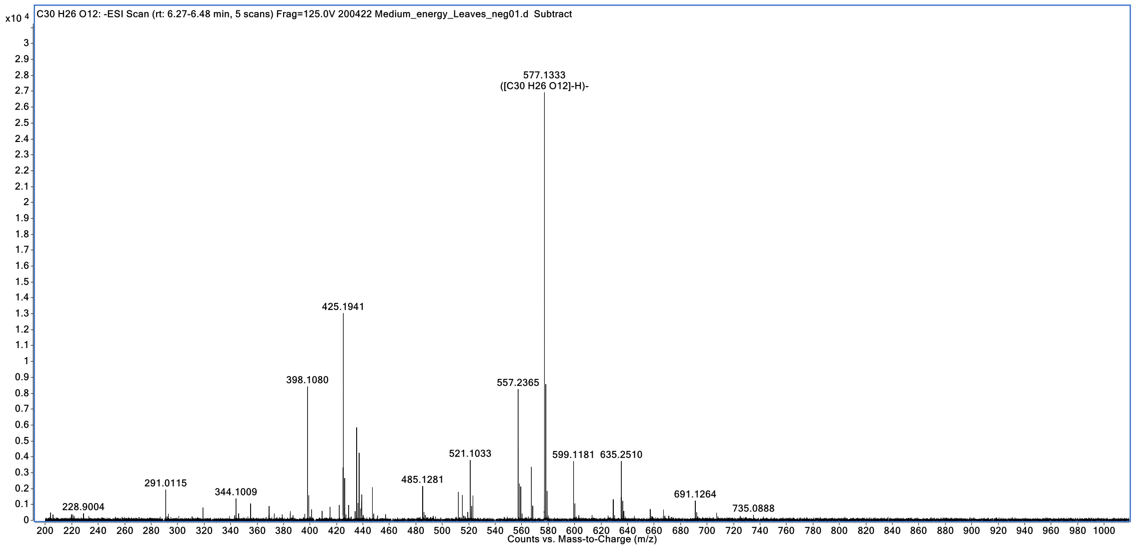

Figure S46. MS^1 spectra data from [M-H]$^-$=577.1333, procyanidin (**22**).

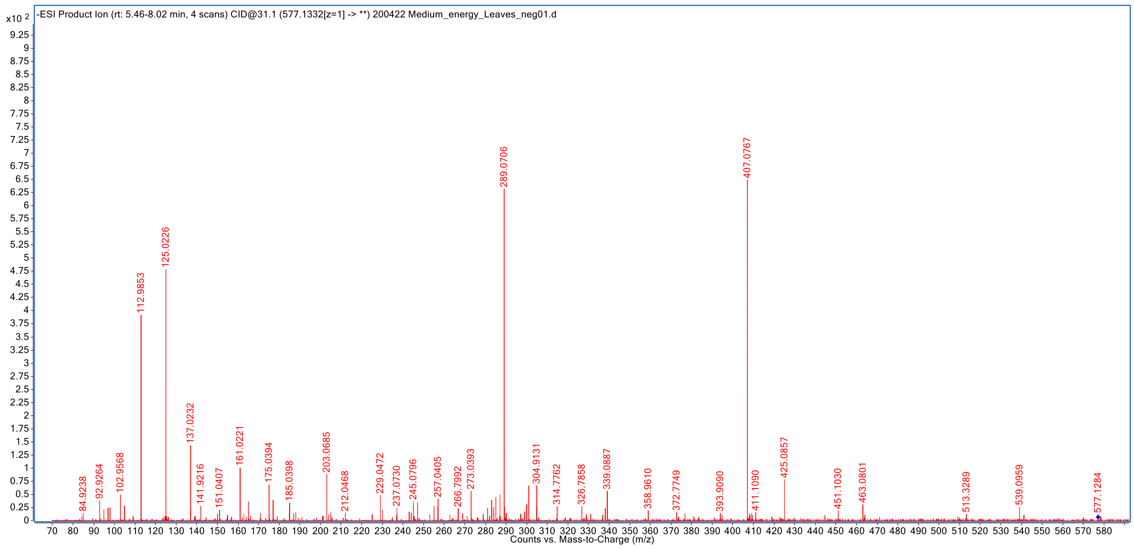

Figure S47. MS^2 spectra data from $[M-H]^-$=577.1333, procyanidin (**22**).

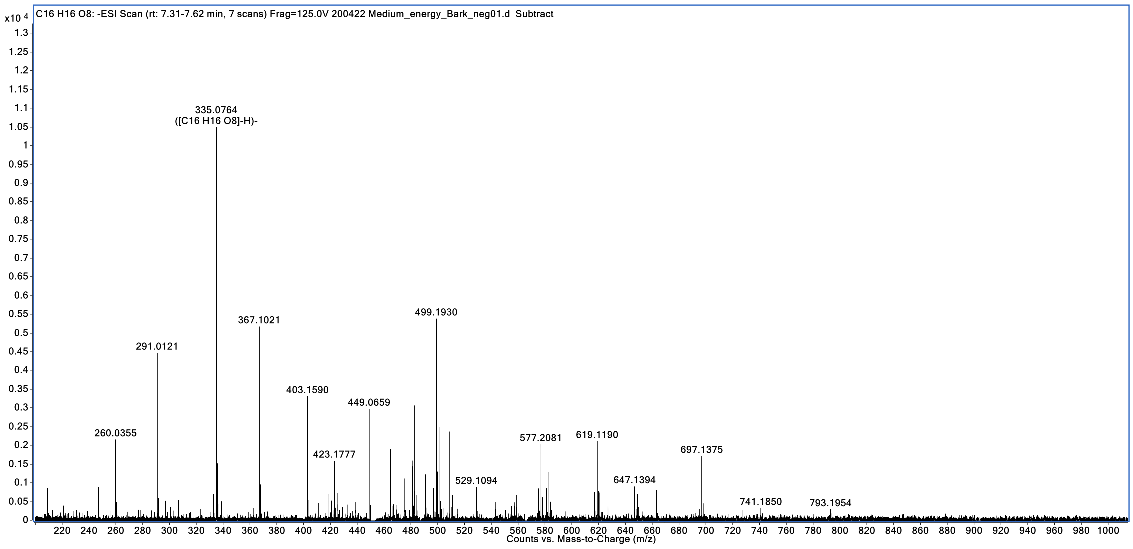

Figure S48. MS^1 spectra data from $[M-H]^-$=335.0764, 3-O-caffeoylshikimic acid (**23**).

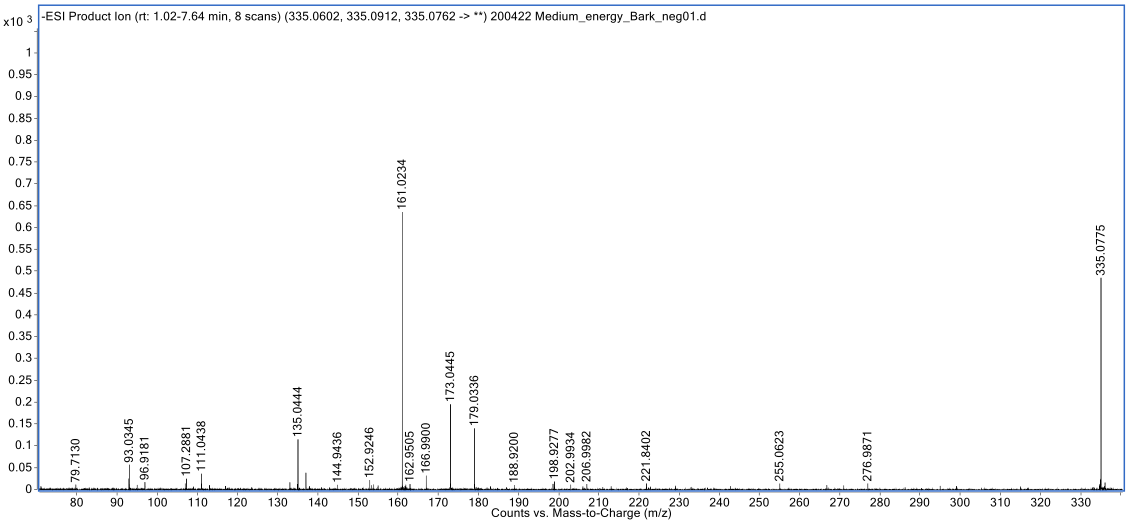

Figure S49. MS^2 spectra data from $[M-H]^-$=335.0764, 3-O-caffeoylshikimic acid (**23**).

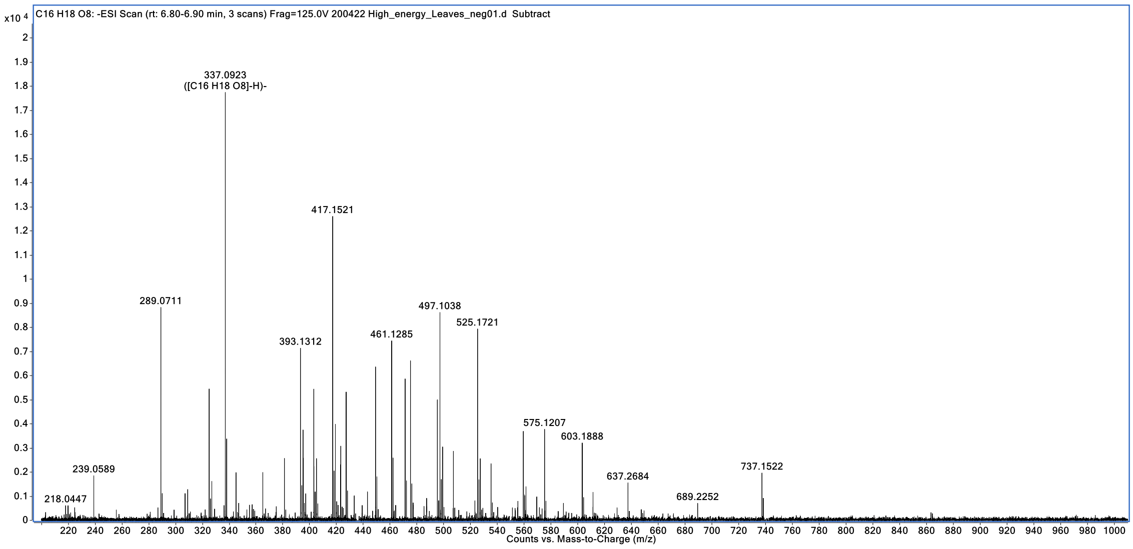

Figure S50. MS^1 spectra data from $[M-H]^-$=337.0923, 3-O-*p*-coumaroylquinic acid (**24**).

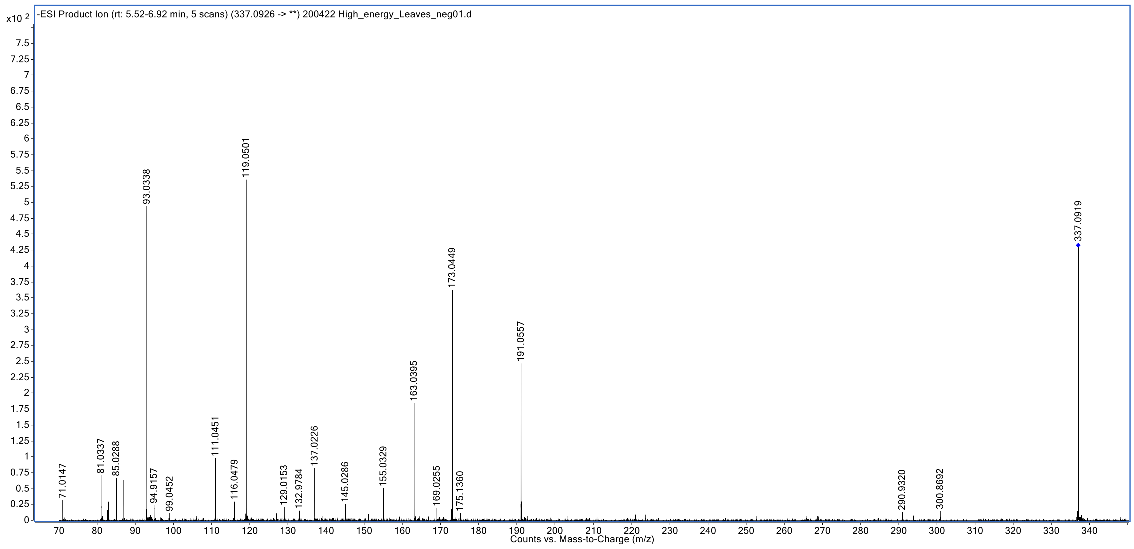

Figure S51. MS^2 spectra data from $[M\text{-}H]^-$=337.0923, 3-O-p-coumaroylquinic acid (**24**).

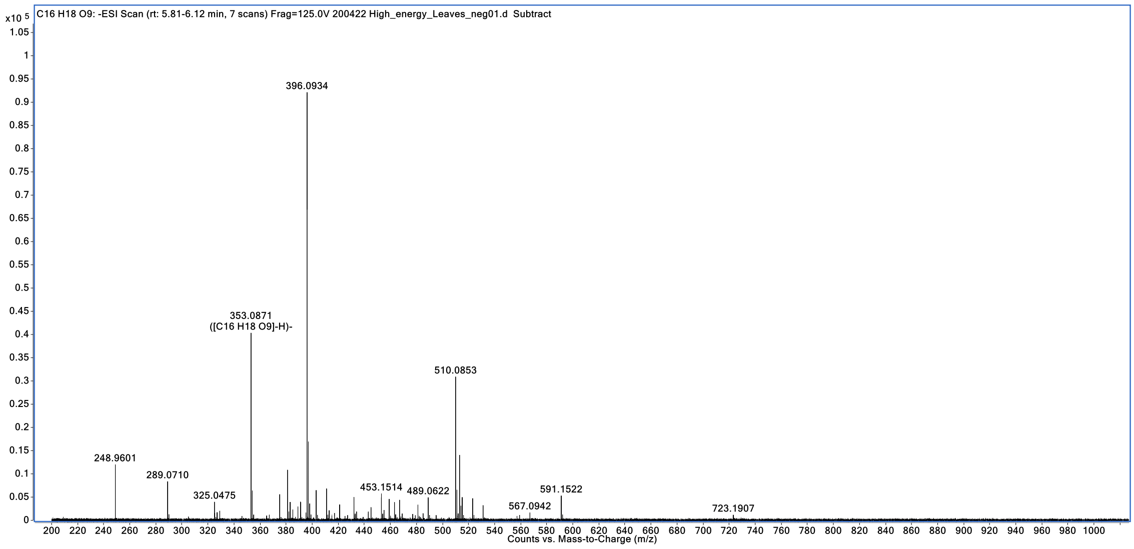

Figure S52. MS^1 spectra data from $[M\text{-}H]^-$=353.0871, chlorogenic acid (**25**).

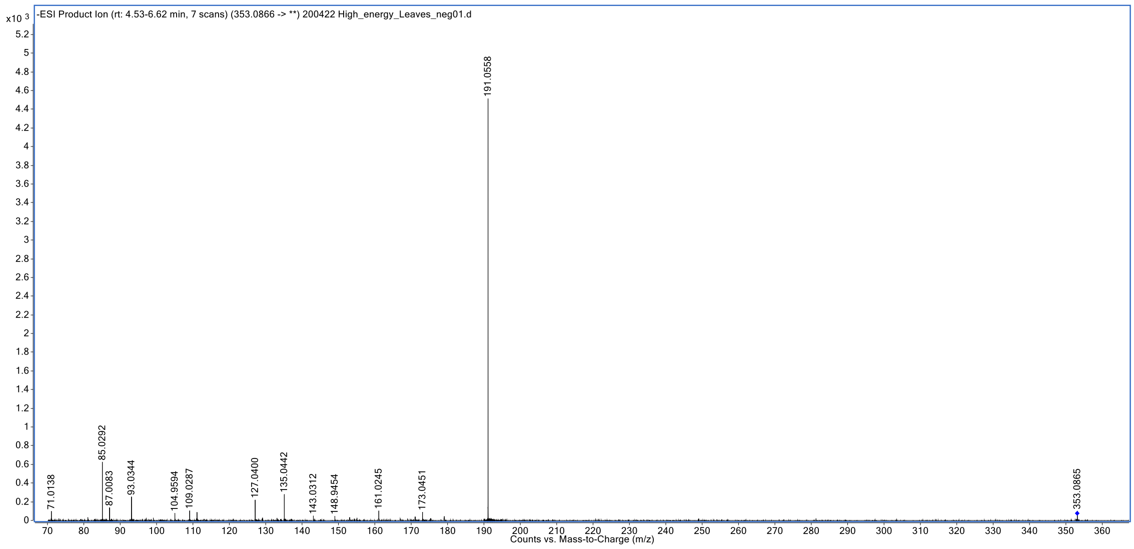

Figure S53. MS^2 spectra data from $[M\text{-}H]^-$=353.0871, chlorogenic acid (**25**).

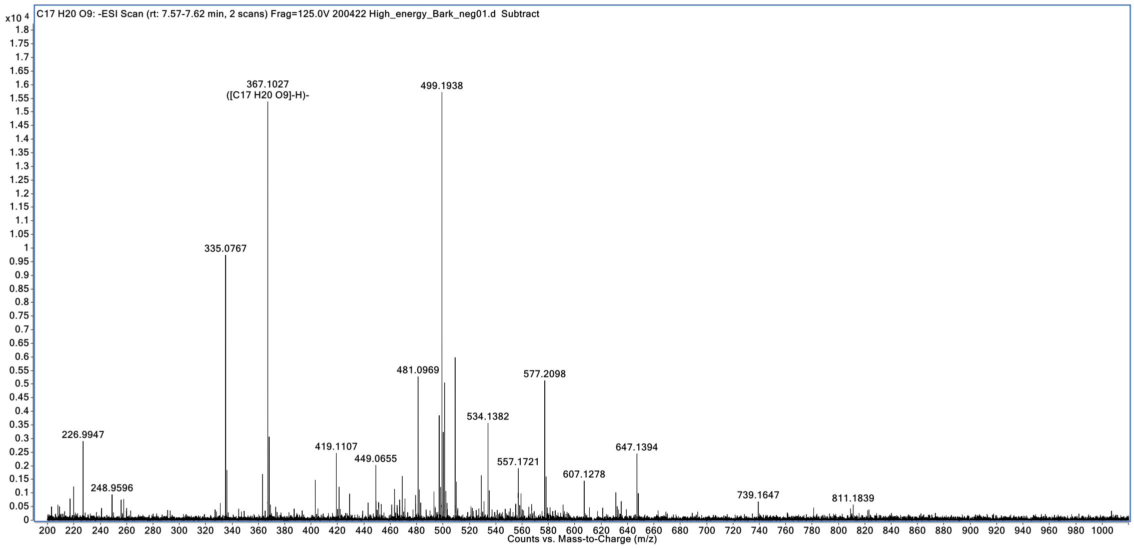

Figure S54. MS[1] spectra data from [M-H]⁻=367.1027, 3-O-caffeoyl-4-O-methylquinic acid (**26**).

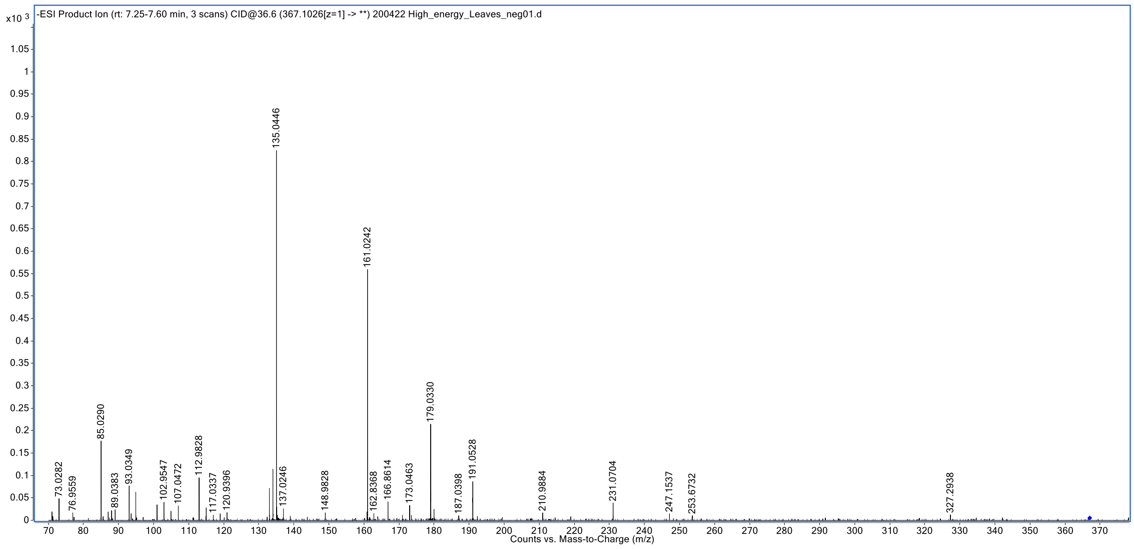

Figure S55. MS[2] spectra data from [M-H]⁻=367.1027, 3-O-caffeoyl-4-O-methylquinic acid (**26**).

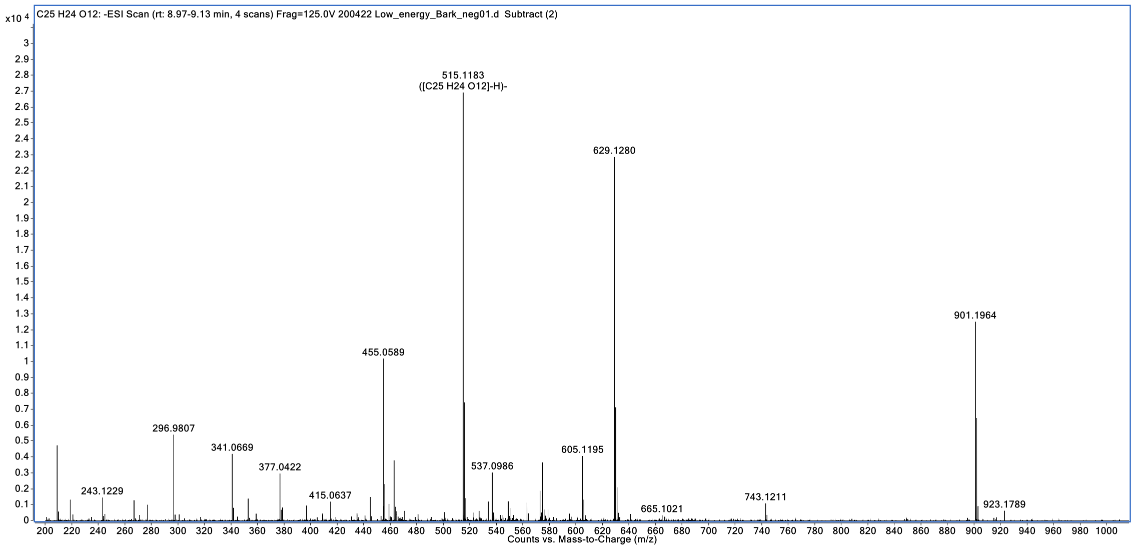

Figure S56. MS[1] spectra data from [M-H]⁻=515.1183, 1,3-dicaffeoylquinic acid (**27**).

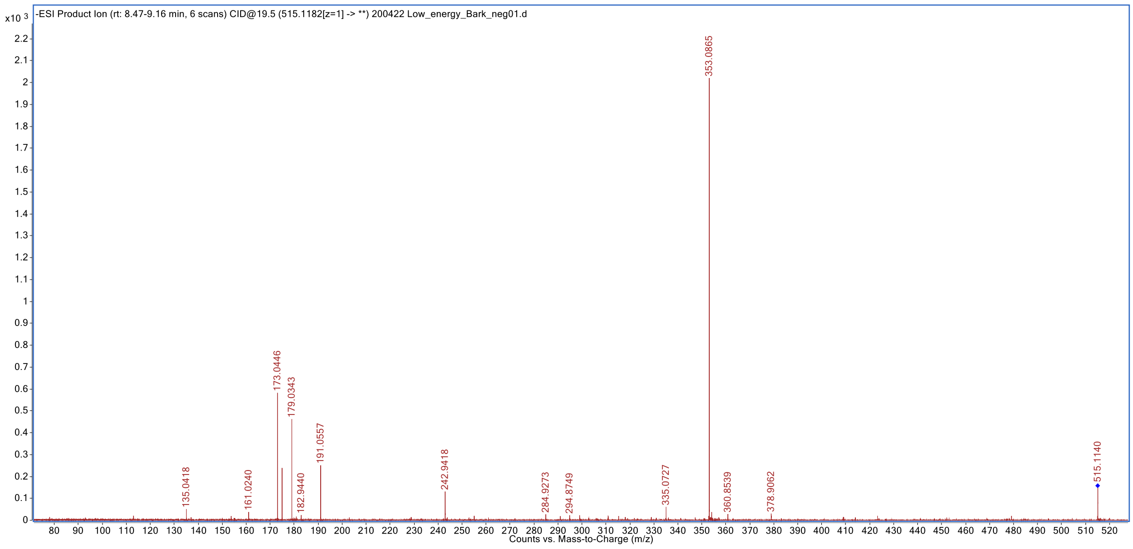

Figure S57. MS[2] spectra data from [M-H]-=515.1183, 1,3-dicaffeoylquinic acid (**27**).

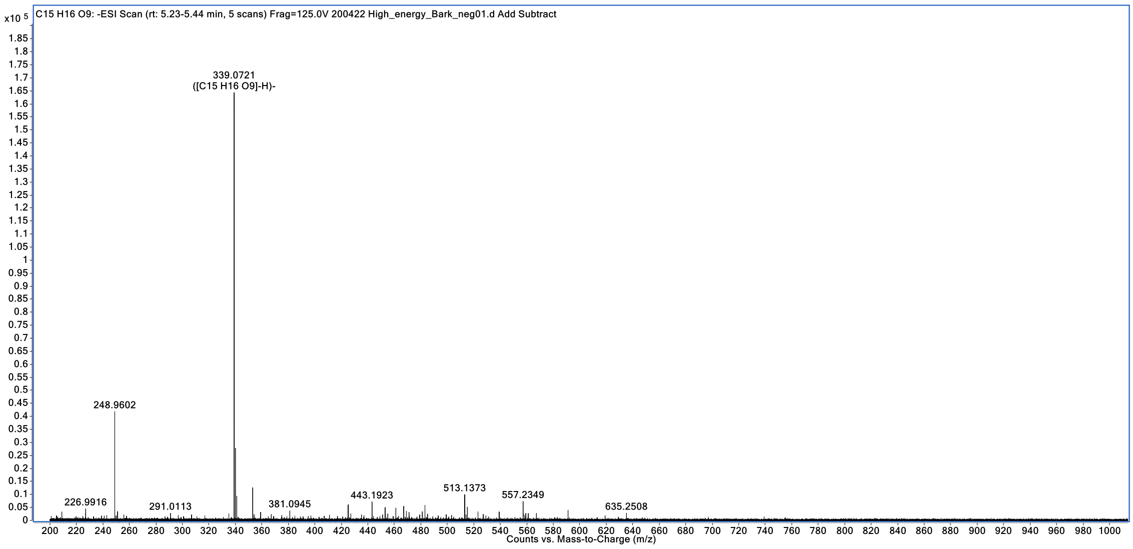

Figure S58. MS[1] spectra data from [M-H]-=339.0721, aesculin (**28**).

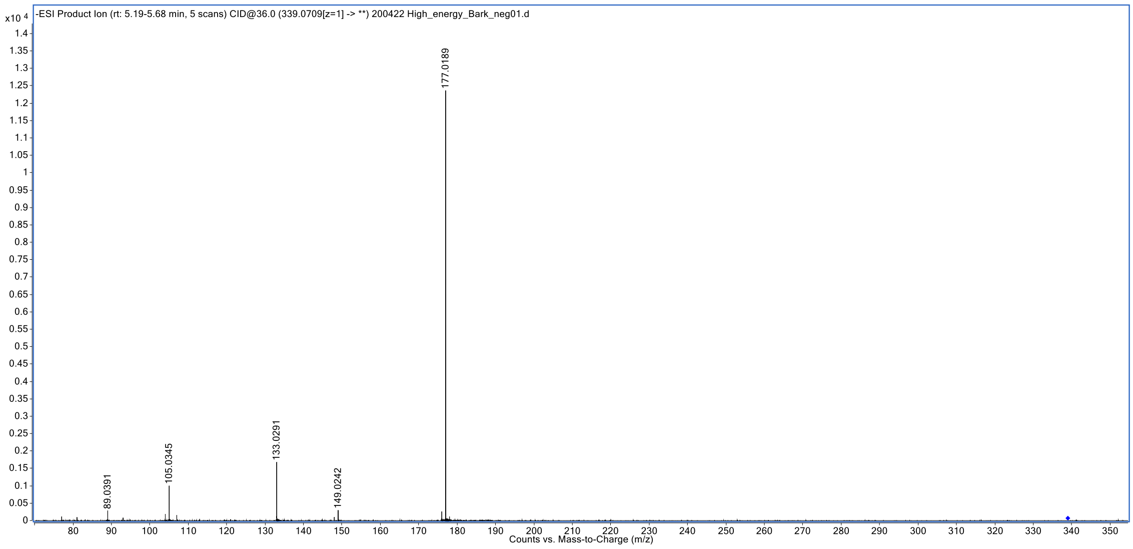

Figure S59. MS[2] spectra data from [M-H]-=339.0721, aesculin (**28**).

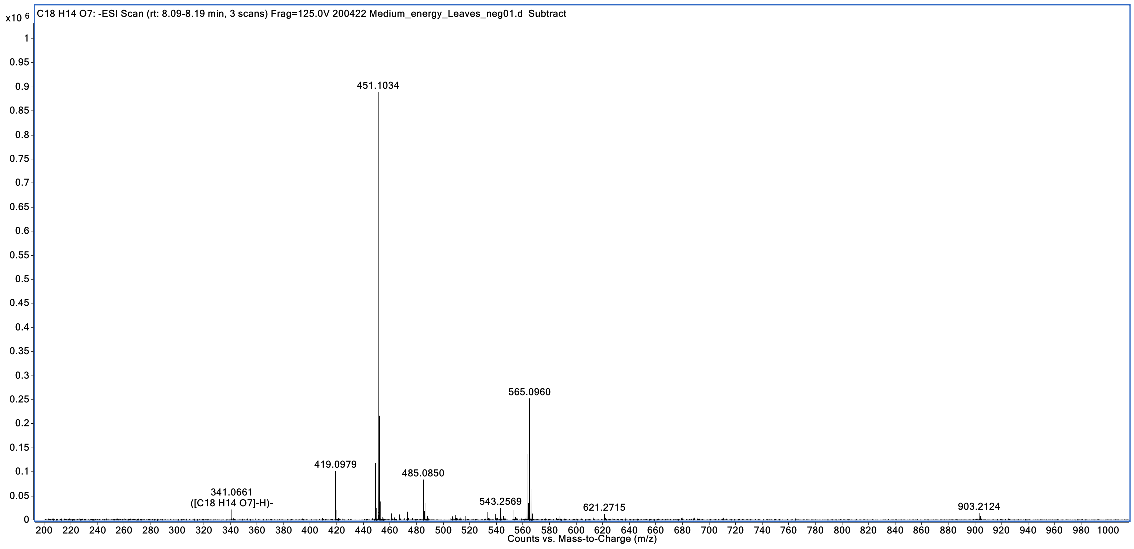

Figure S60. MS¹ spectra data from [M-H]⁻=341.0661, phyllocoumarin (**29**).

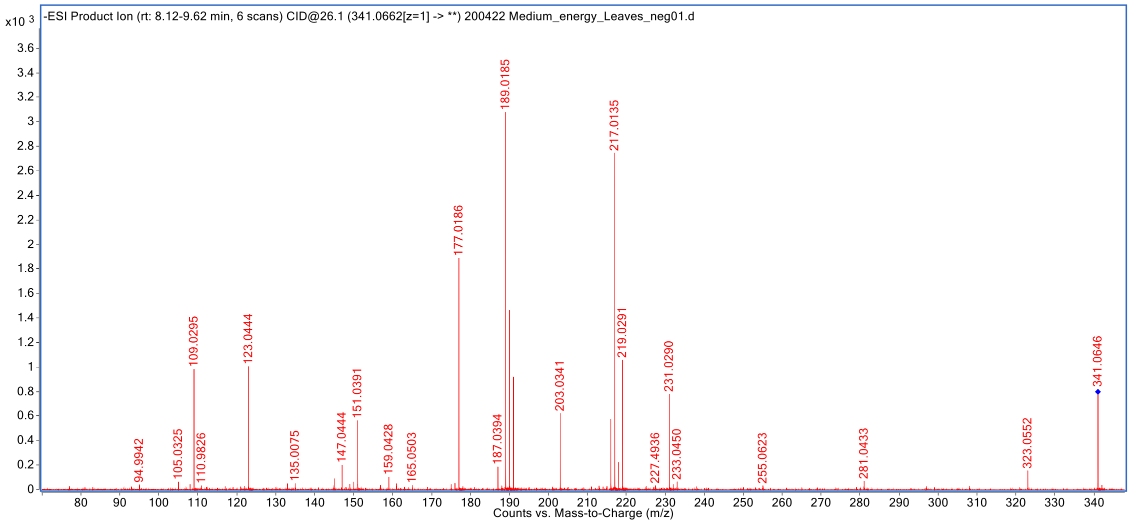

Figure S61. MS² spectra data from [M-H]⁻=341.0661, phyllocoumarin (**29**).

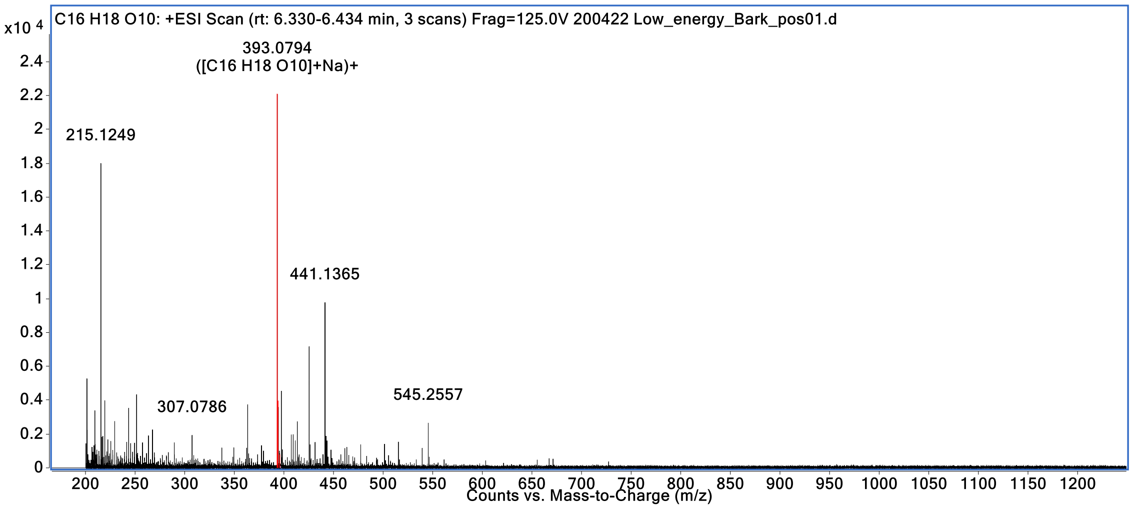

Figure S62. MS1 spectra from [M+Na]+=393.0794, fraxin (**30**).

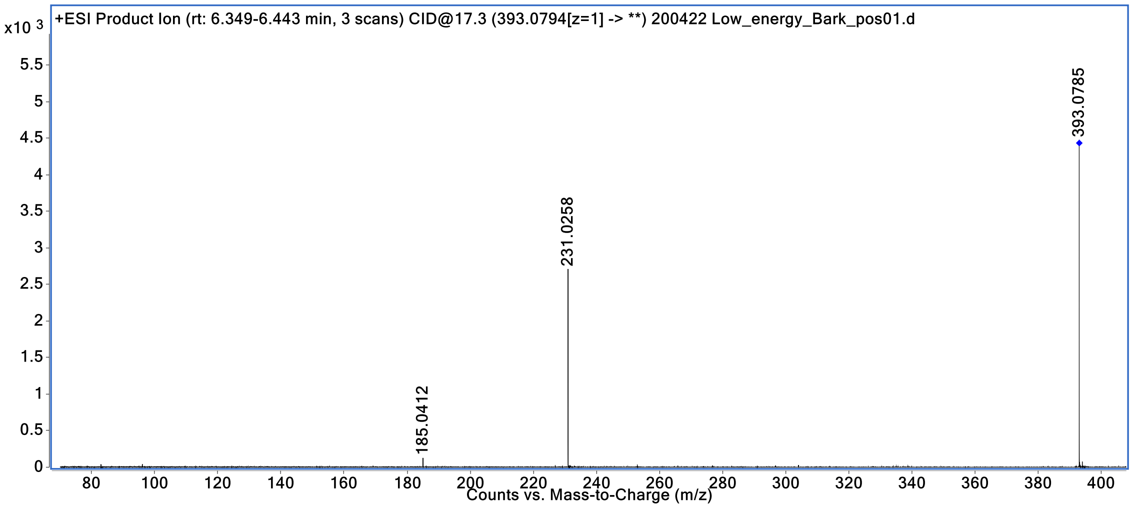

Figure S63. MS2 spectra from [M+Na]+=393.0794, fraxin (**30**).

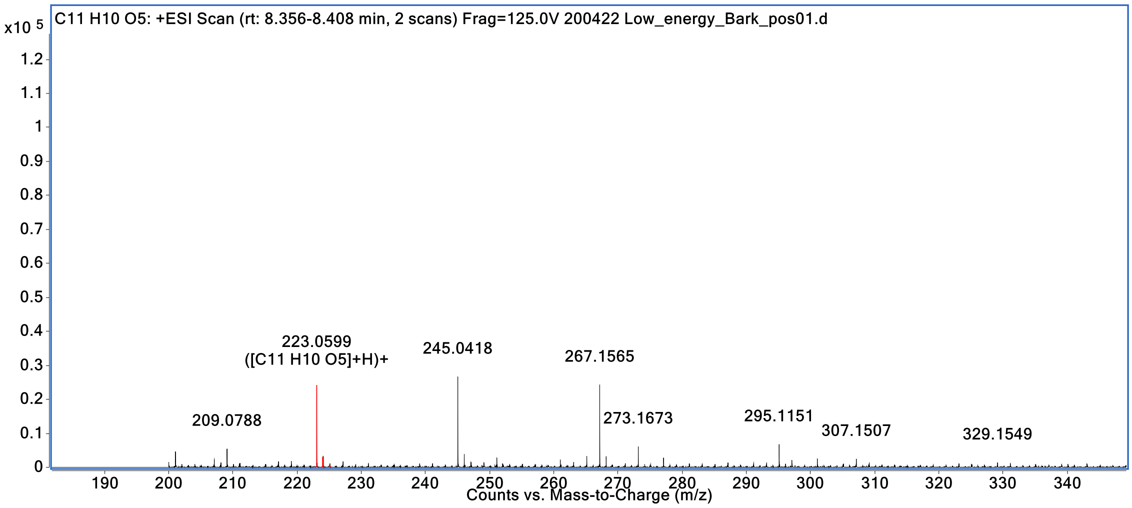

Figure S64. MS1 Spectra from [M+H]+=223.0599, fraxidin (**31**).

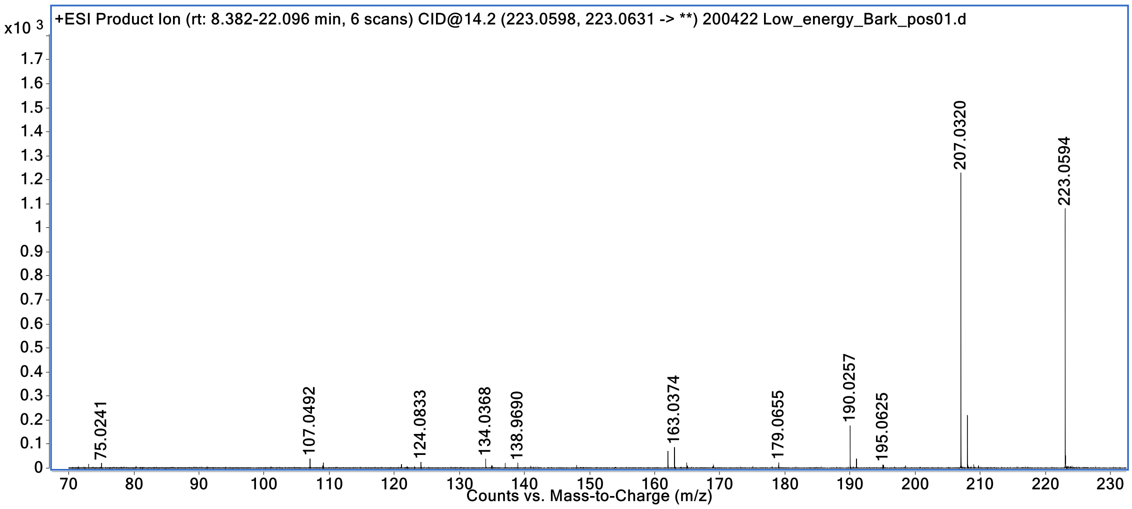

Figure S65. MS2 Spectra from [M+H]+=223.0599, fraxidin (**31**).

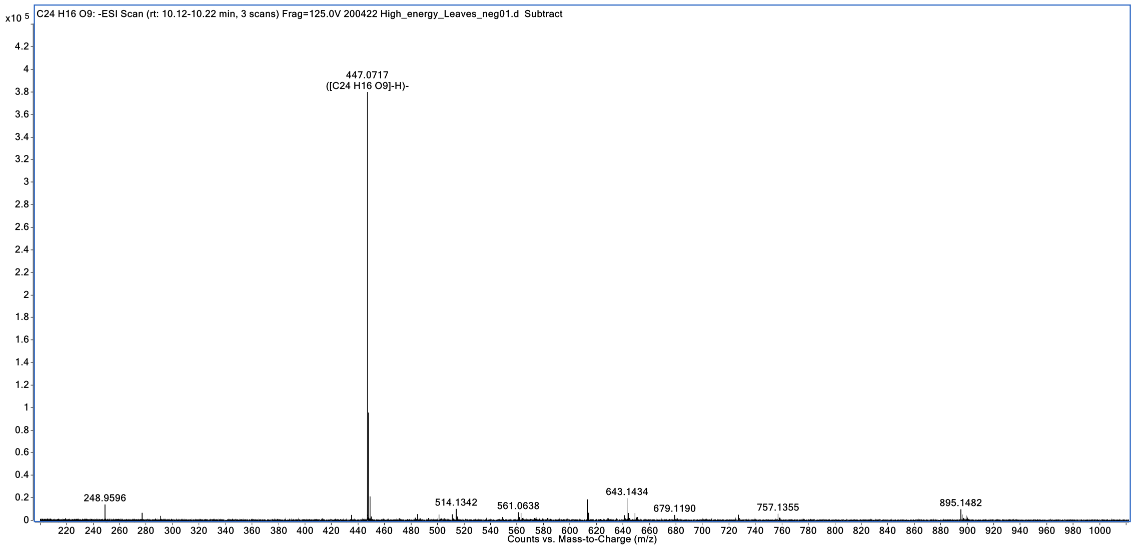

Figure S66. MS[1] spectra data from $[M\text{-}H]^-$=447.0717, naringenin-(3→8)-5,7-dihydroxychromone (**32**).

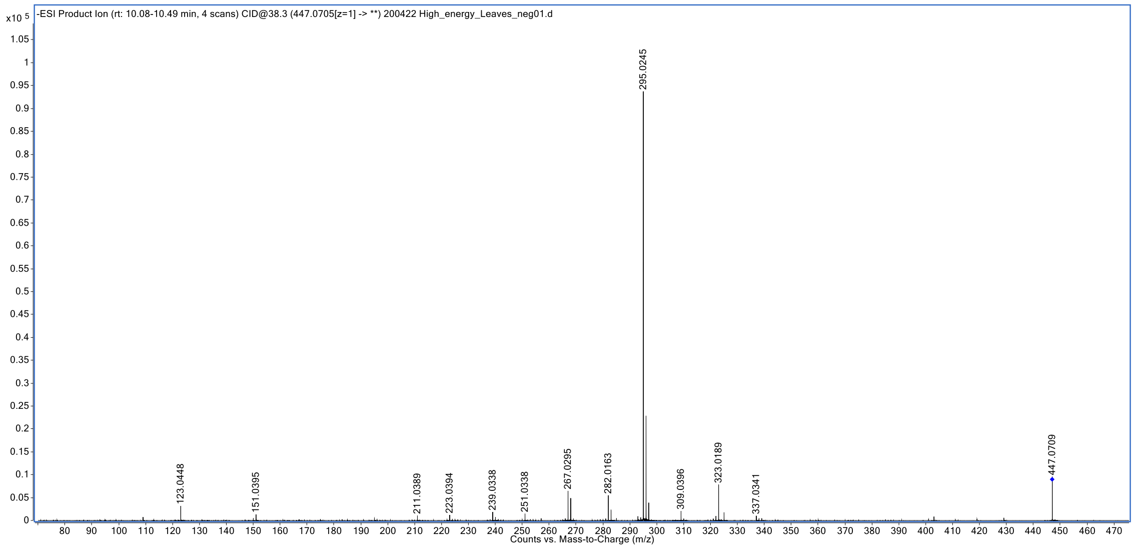

Figure S67. MS[2] spectra data from $[M\text{-}H]^-$=447.0717, naringenin-(3→8)-5,7-dihydroxychromone (**32**).

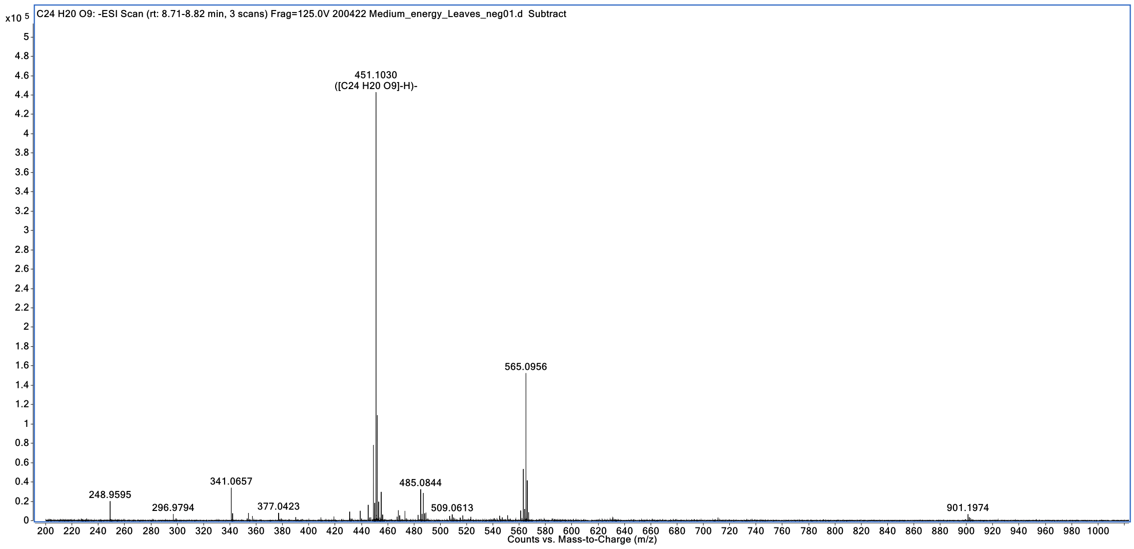

Figure S68. MS[1] spectra data from $[M\text{-}H]^-$=451.1030, cinchonain l (**33**).

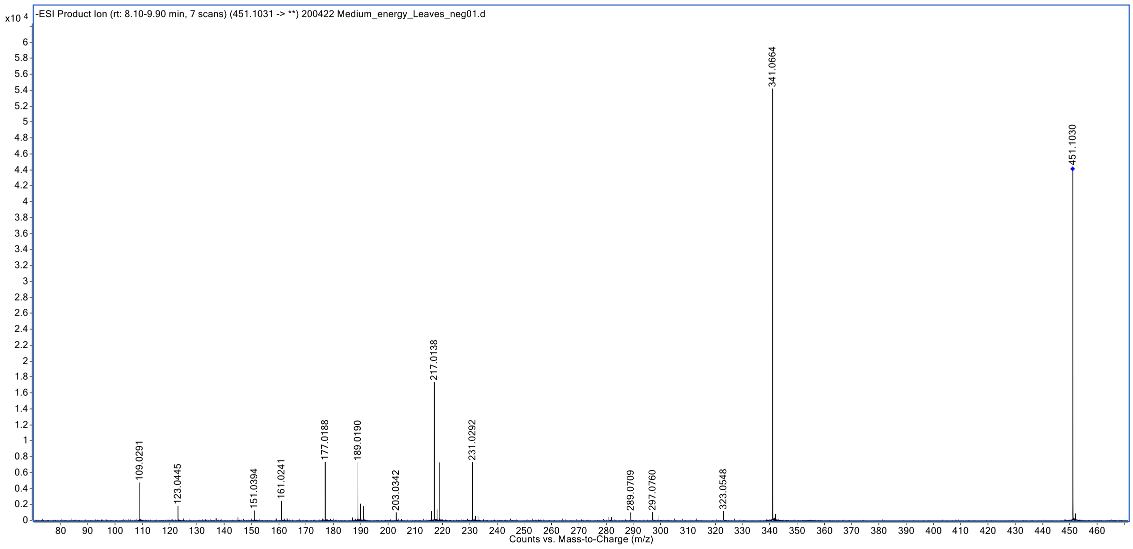

Figure S69. MS² spectra data from [M-H]⁻=451.1030, cinchonain l (**33**).

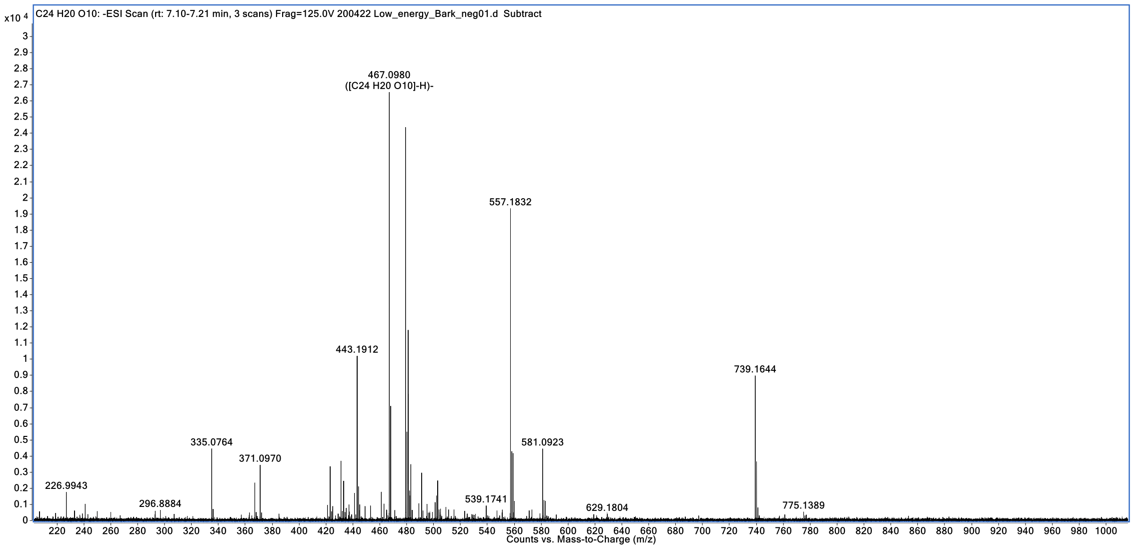

Figure S70. MS¹ spectra data from [M-H]⁻=467.0980, apocynin (**34**).

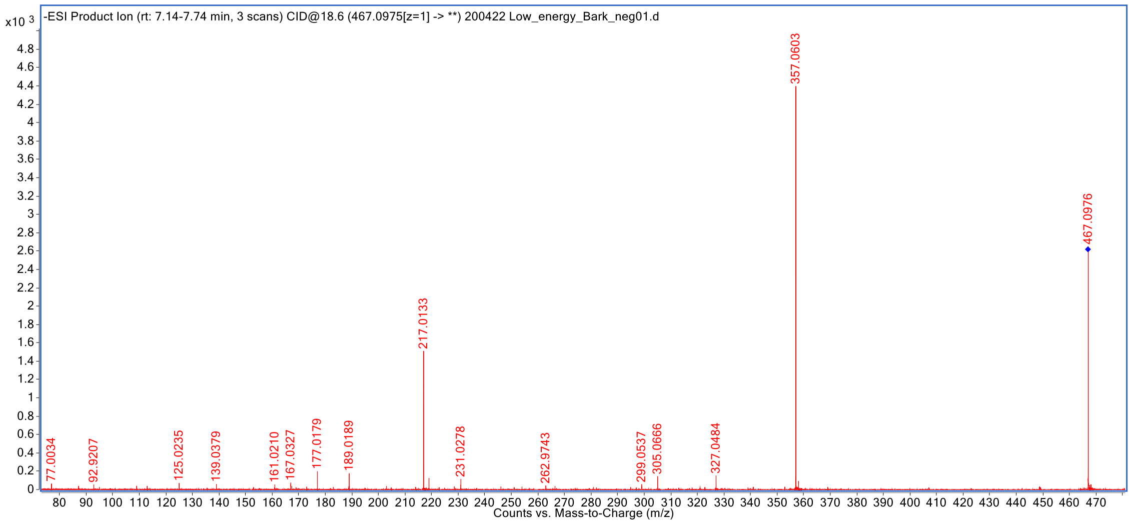

Figure S71. MS² spectra data from [M-H]⁻=467.0980, apocynin (**34**).

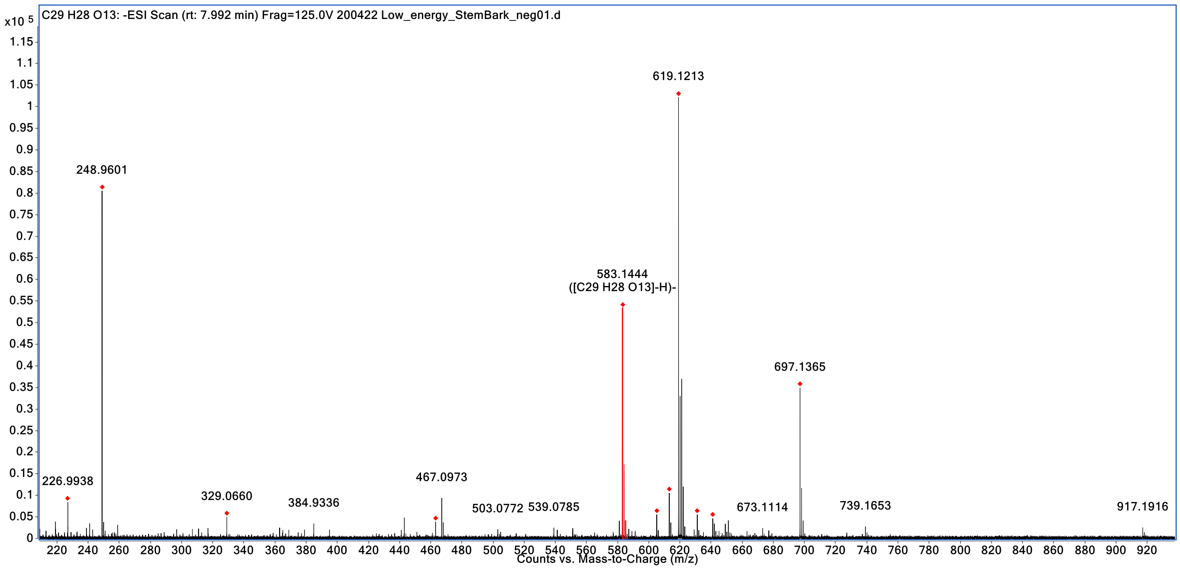

Figure S72. MS^1 spectra data from $[M-H]^-$=583.1444, cinchonain l derivative I (**35**).

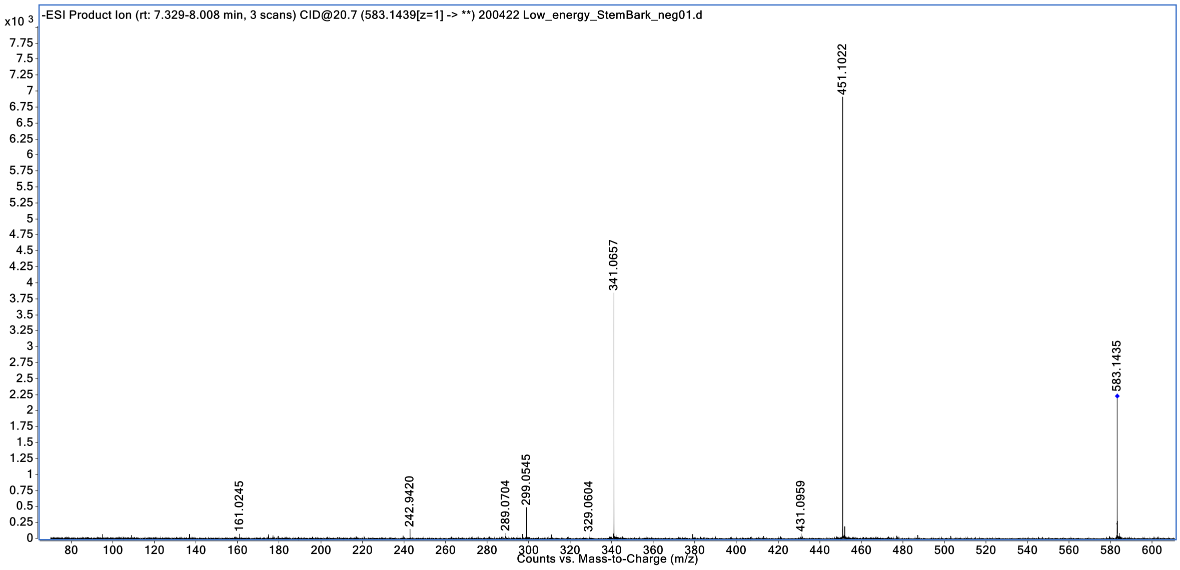

Figure S73. MS^2 spectra data from $[M-H]^-$=583.1444, cinchonain l derivative I (**35**).

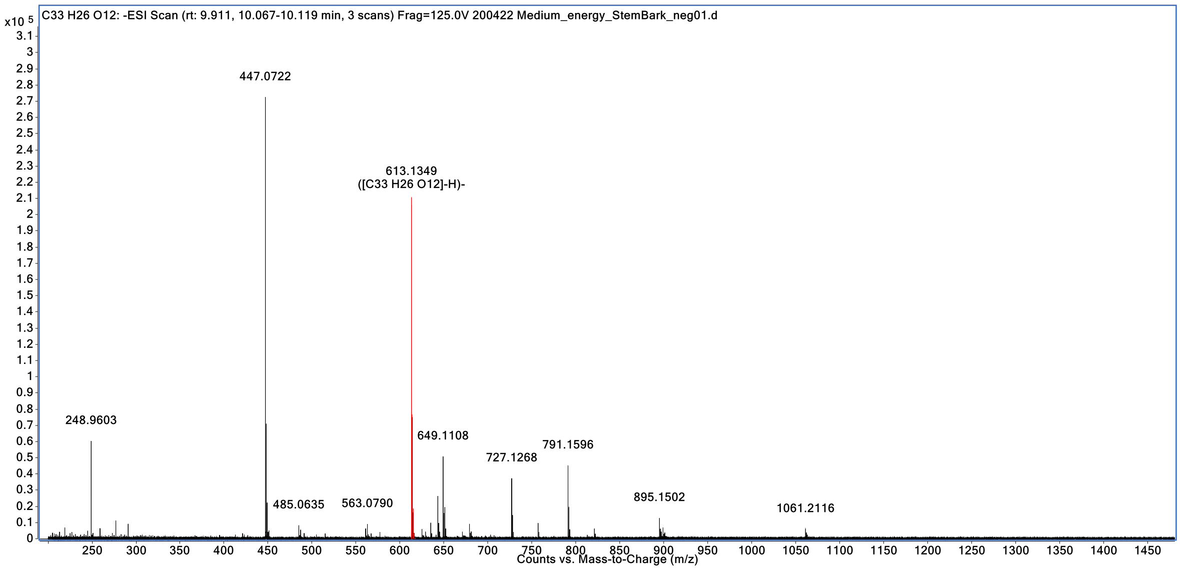

Figure S74. MS^1 spectra data from $[M-H]^-$=613.1349, cinchonain l derivative II (**36**).

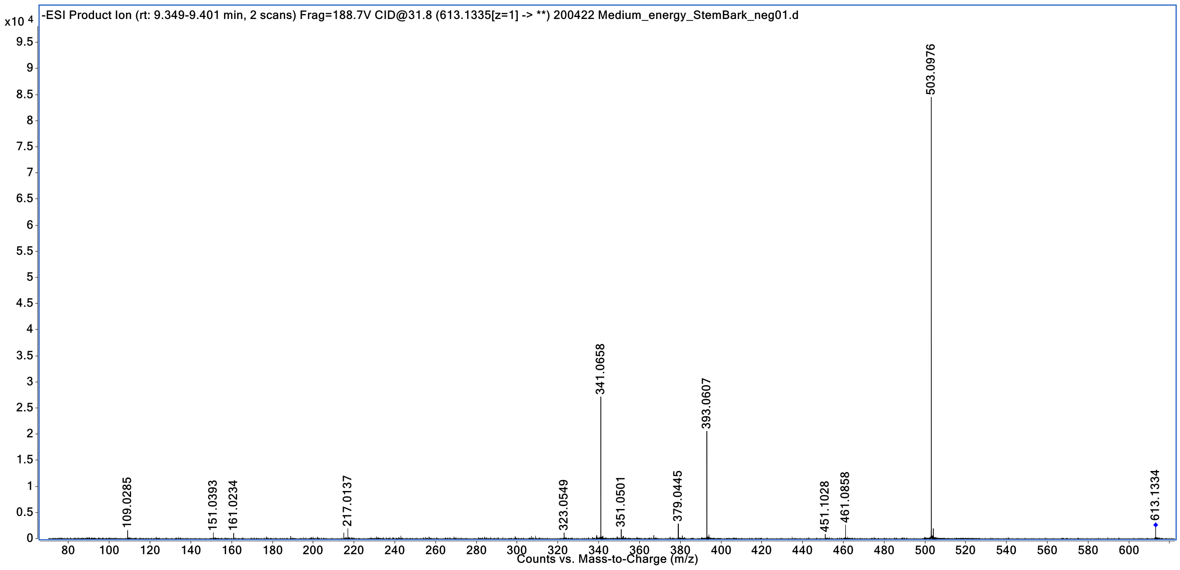

Figure S75. MS² spectra data from [M-H]⁻=613.1349, cinchonain l derivative II (**36**).

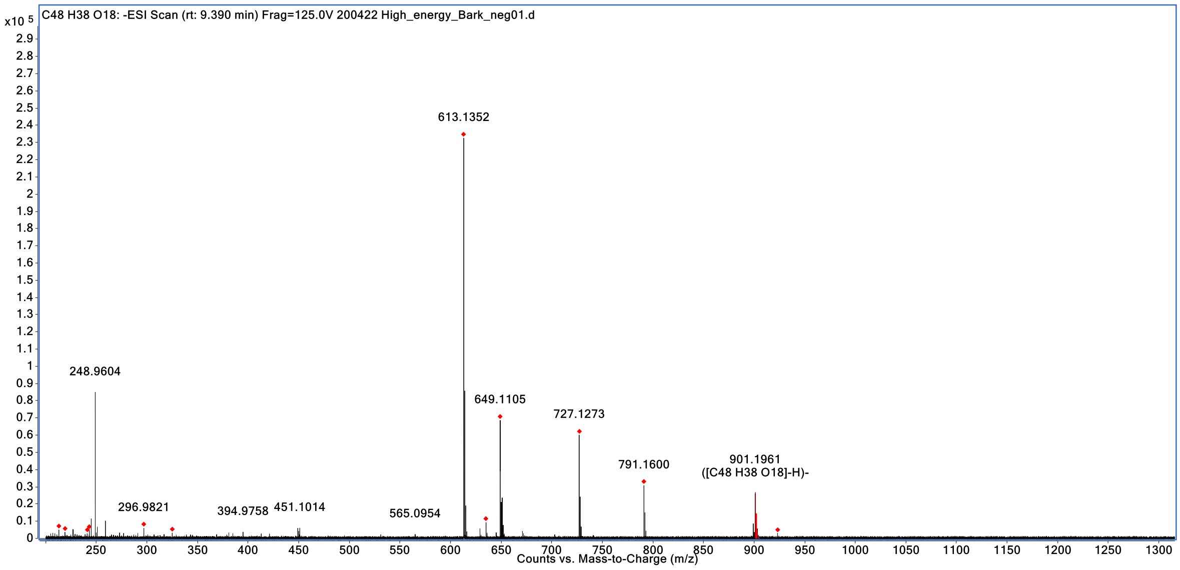

Figure S76. MS¹ spectra data from [M-H]⁻=901.1961, cinchonain l derivative III (**37**).

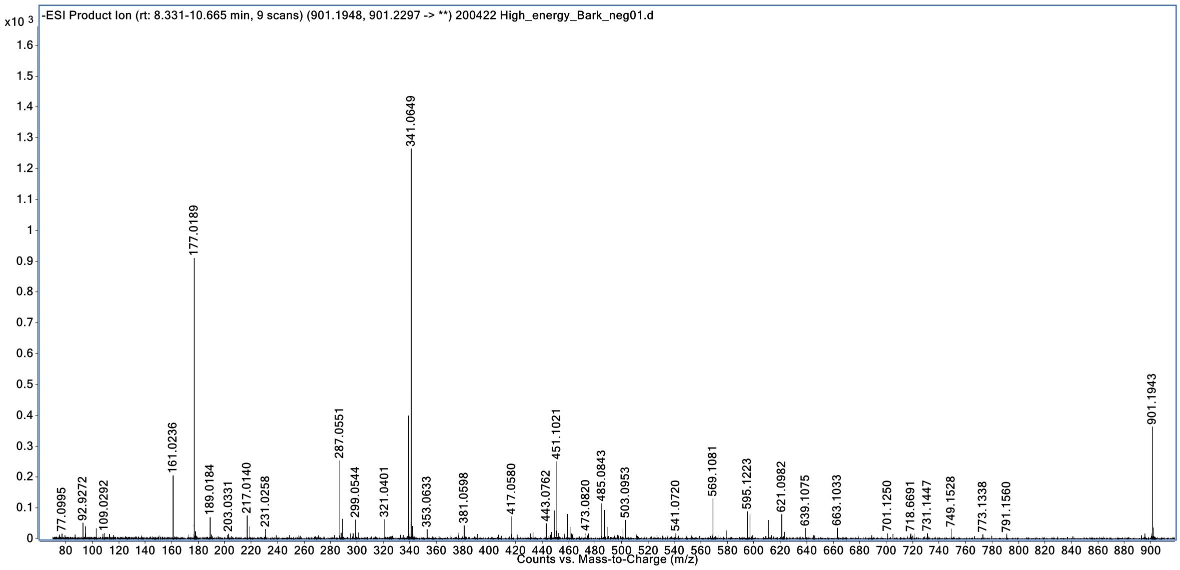

Figure S77. MS² spectra data from [M-H]⁻=901.1961, cinchonain l derivative III (**37**).

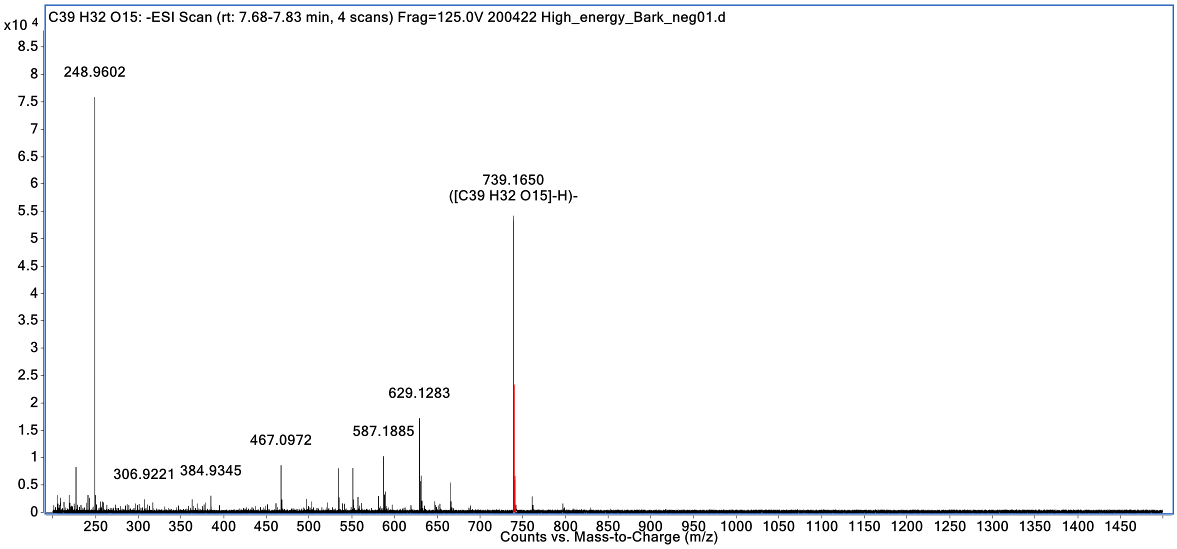

Figure S78. MS1 spectra data from [M-H]-=739.1650, cinchonain ll (**38**).

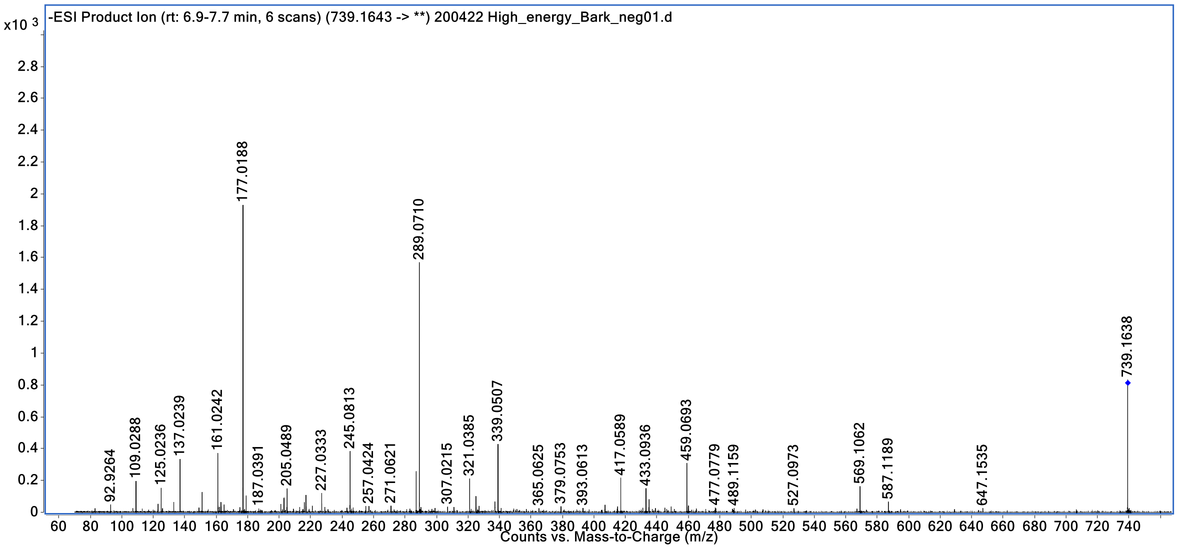

Figure S79. MS2 spectra data from [M-H]-=739.1650, cinchonain ll (**38**).

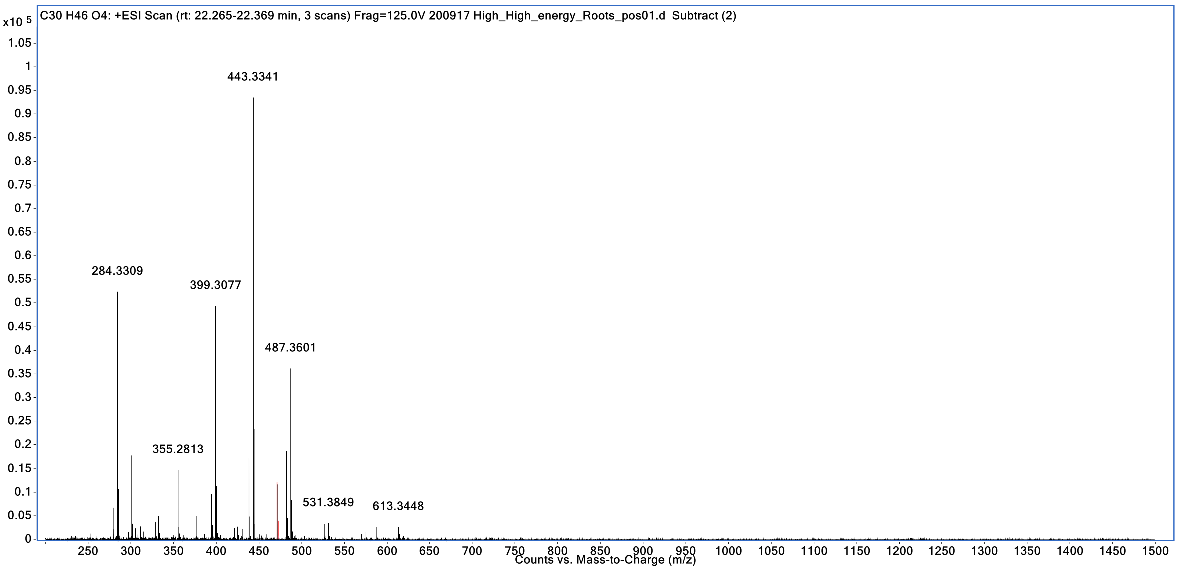

Figure S80. MS[1] spectra data from [M-H]=471.3459, 11-oxooleanolic acid (**39**).

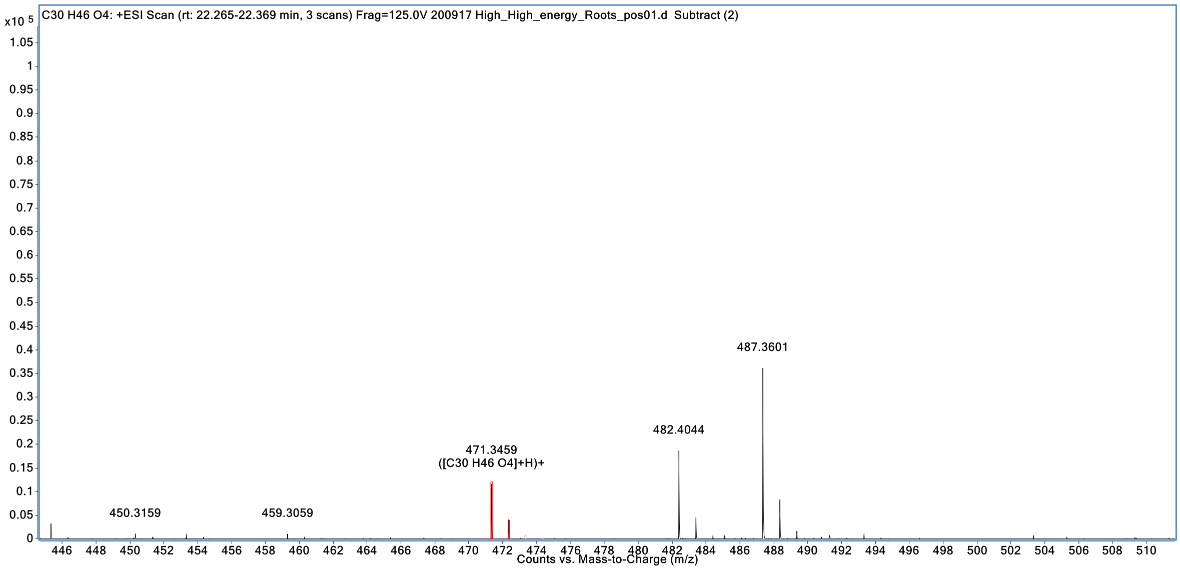

Figure S81. Expansion of MS[1] spectra data from [M-H]⁻=471.3459, 11-oxooleanolic acid (**39**).

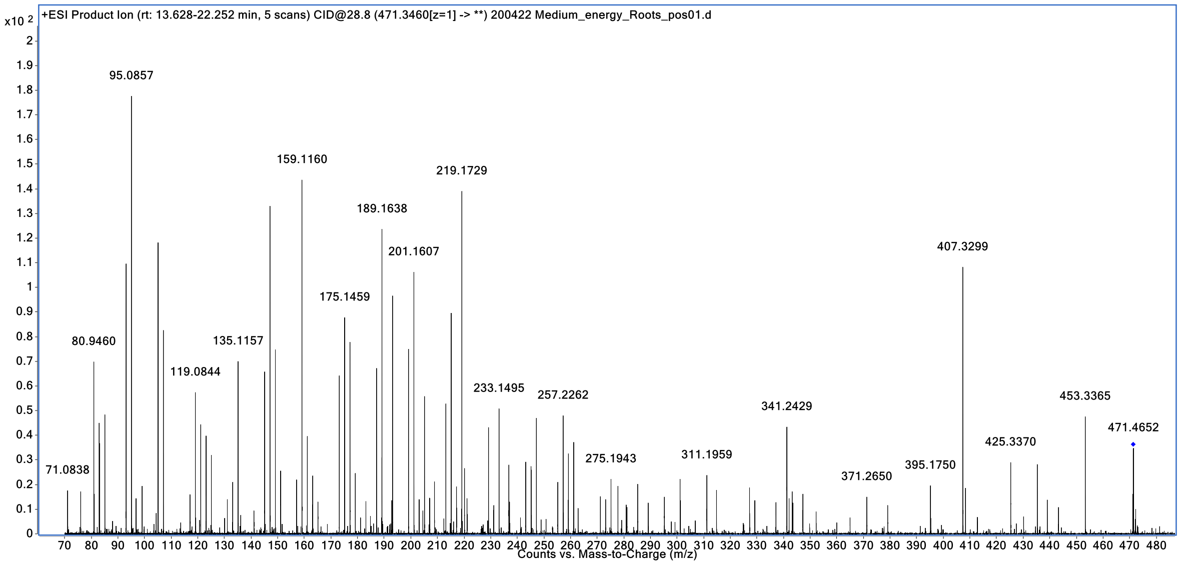

Figure S82. MS[2] spectra data from [M-H]⁻=471.3459, 11-oxooleanolic acid (**39**).

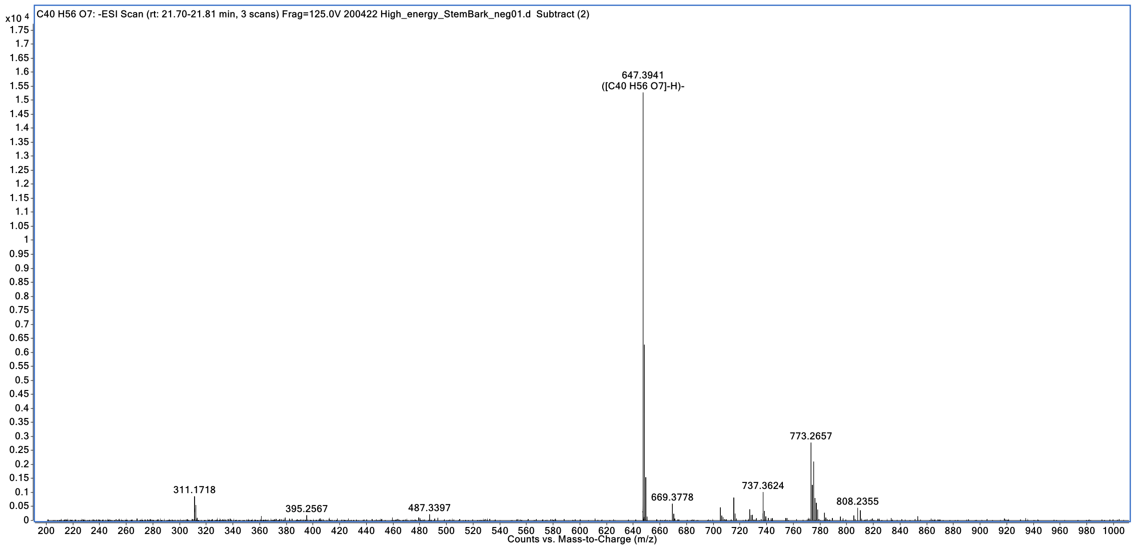

Figure S83. MS[1] spectra data from [M-H]⁻=647.3941, triterpene esterified with ferulic acid (**40**).

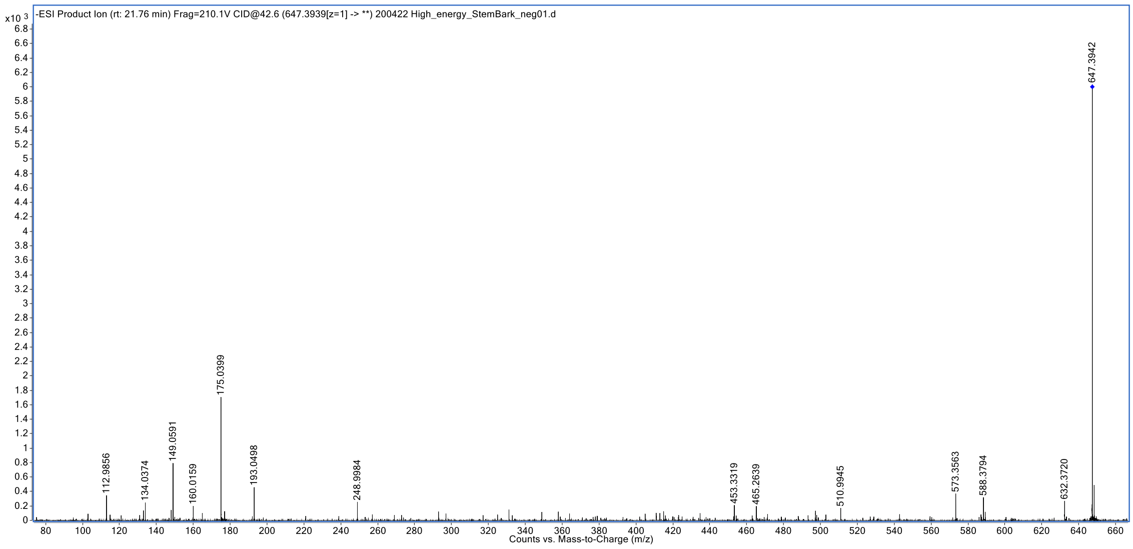

Figure S84. MS^2 spectra data from $[M-H]^-$=647.3941, triterpene esterified with ferulic acid (**40**).

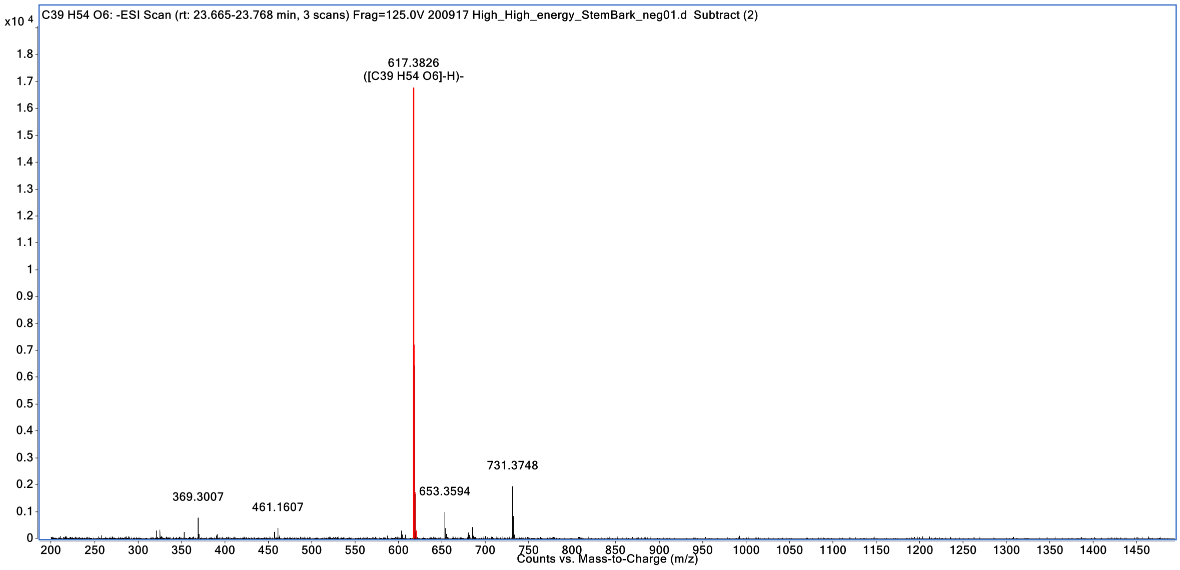

Figure S85. MS^1 spectra data from $[M-H]^-$=617.3826, triterpene esterified with caffeic acid I (**41**).

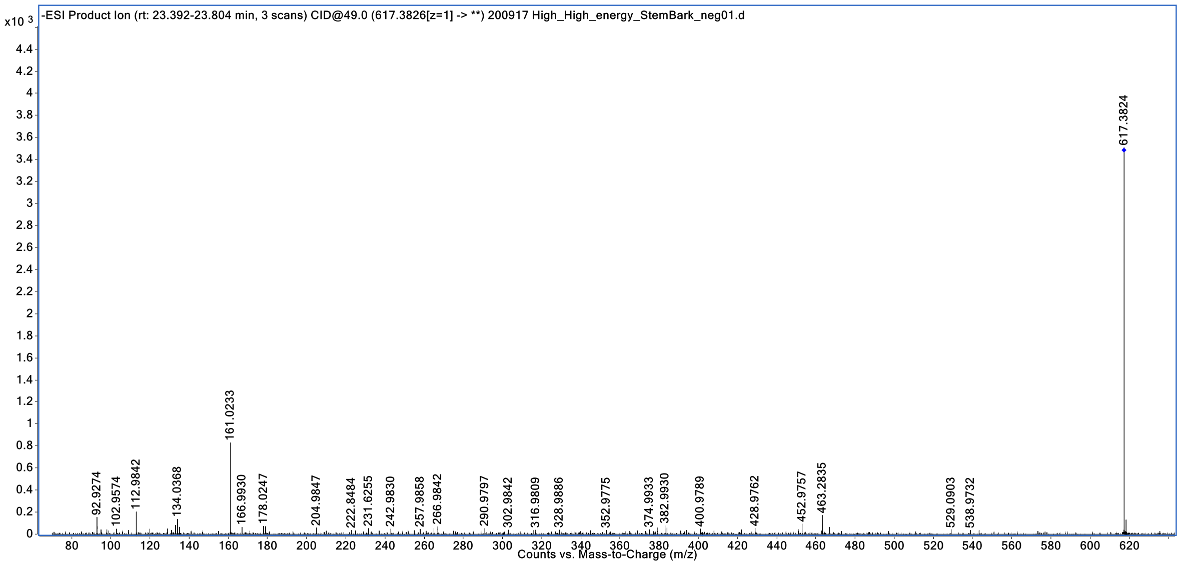

Figure S86. MS^2 spectra data from [M-H]$^-$=617.3826, triterpene esterified with caffeic acid I (**41**).

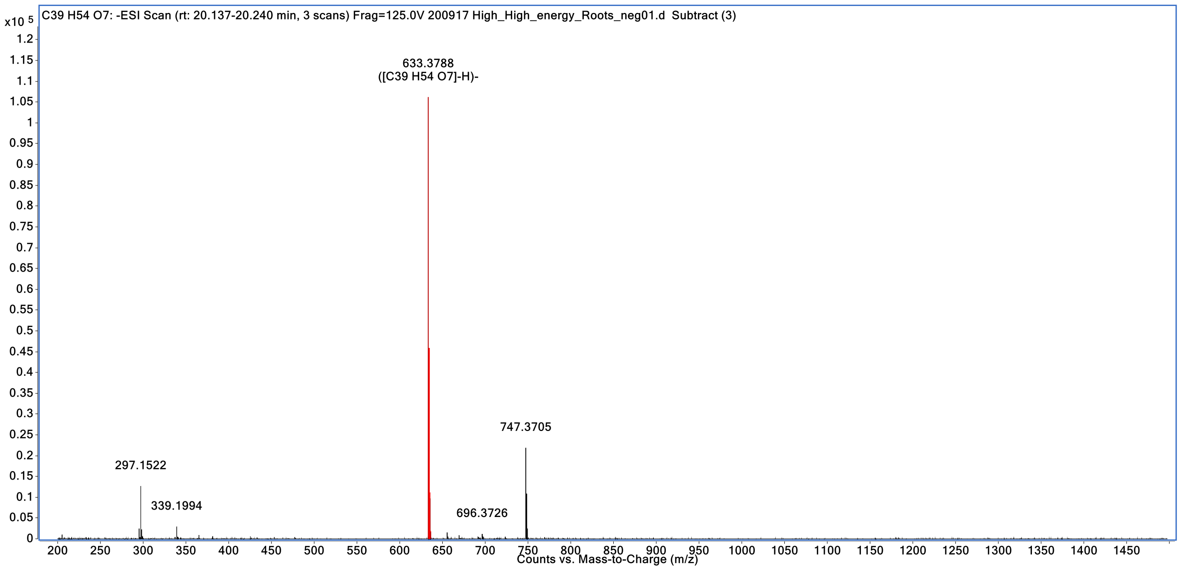

Figure S87. MS^1 spectra data from [M-H]$^-$=633.3788, triterpene esterified with caffeic acid II (**42**).

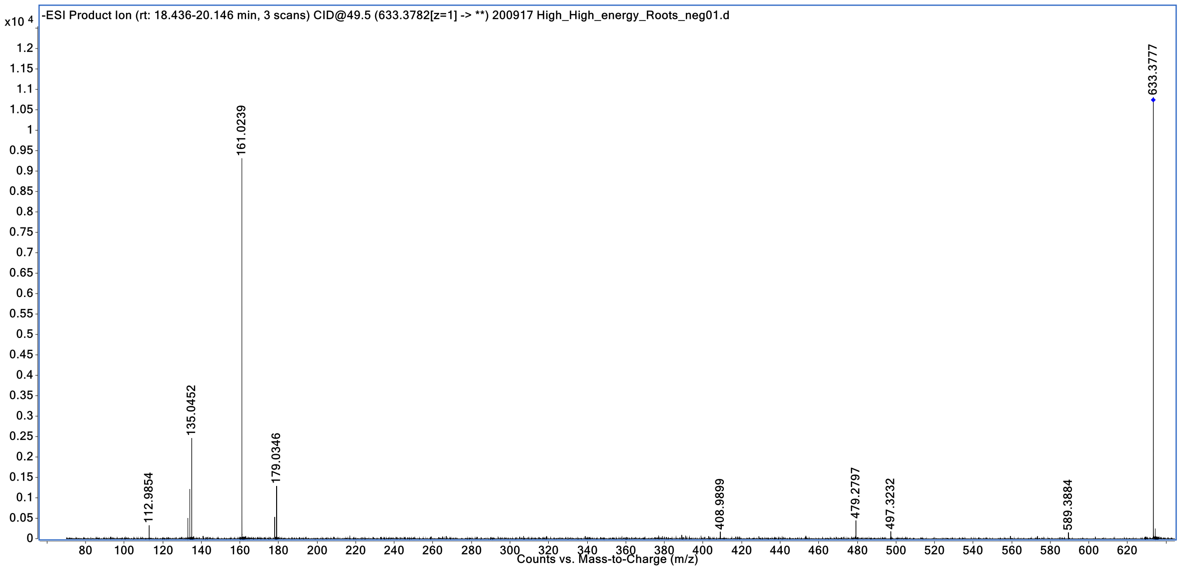

Figure S88. MS^2 spectra data from [M-H]$^-$=633.3788, triterpene esterified with caffeic acid II (**42**).

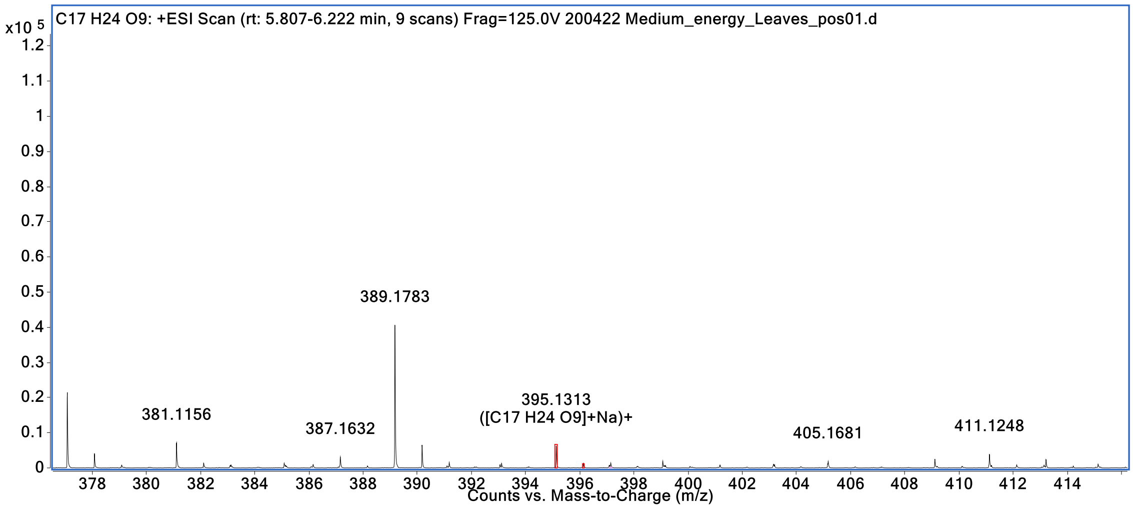

Figure S89. MS1 Spectra from [M+Na]+=395.1313, syringin (**43**).

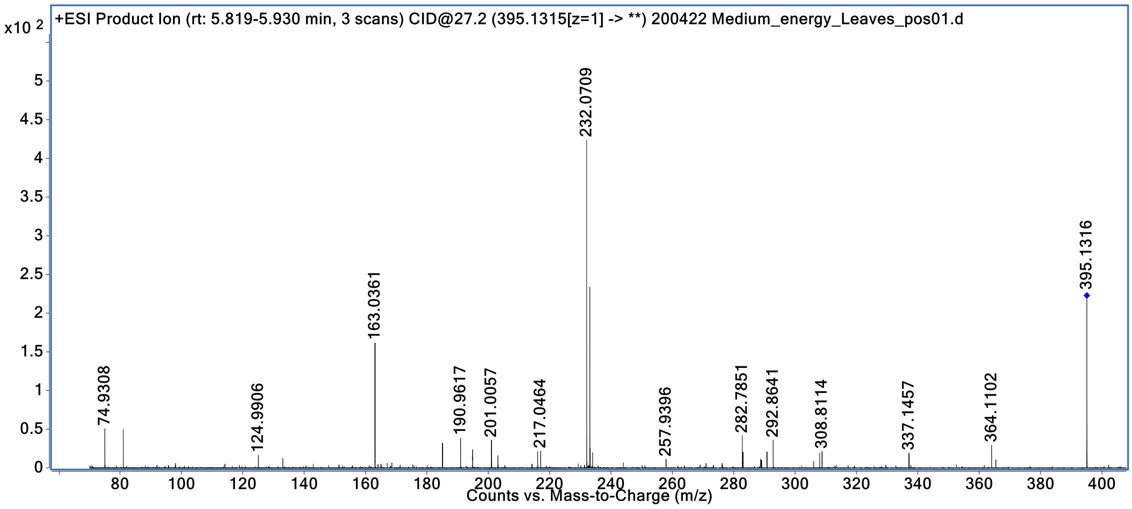

Figure S90. MS2 Spectra from [M+H]+=395.1313, syringin (**43**).

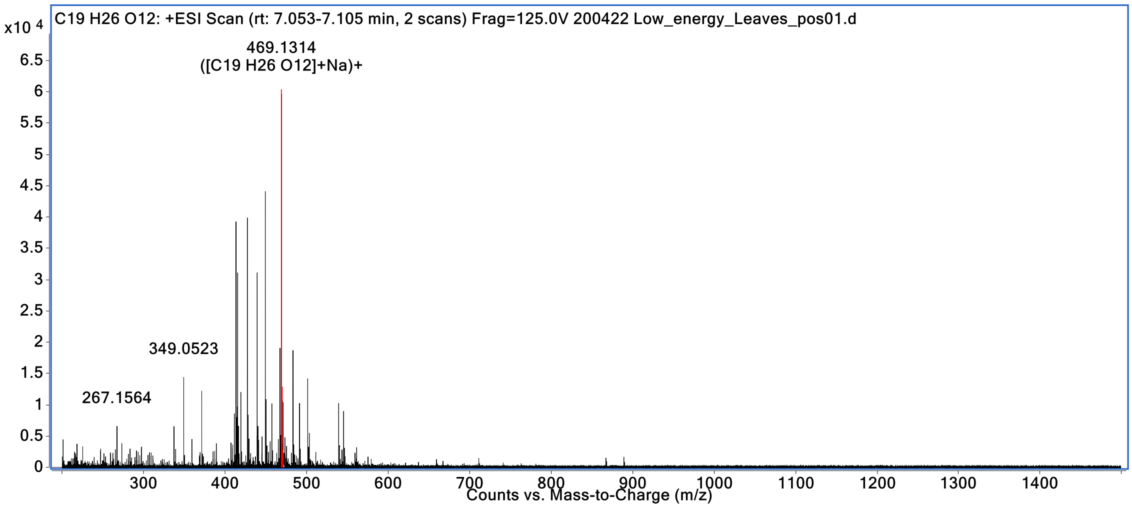

Figure S91. MS1 spectra from [M+Na]+=469.1314, gaultherin (**44**).

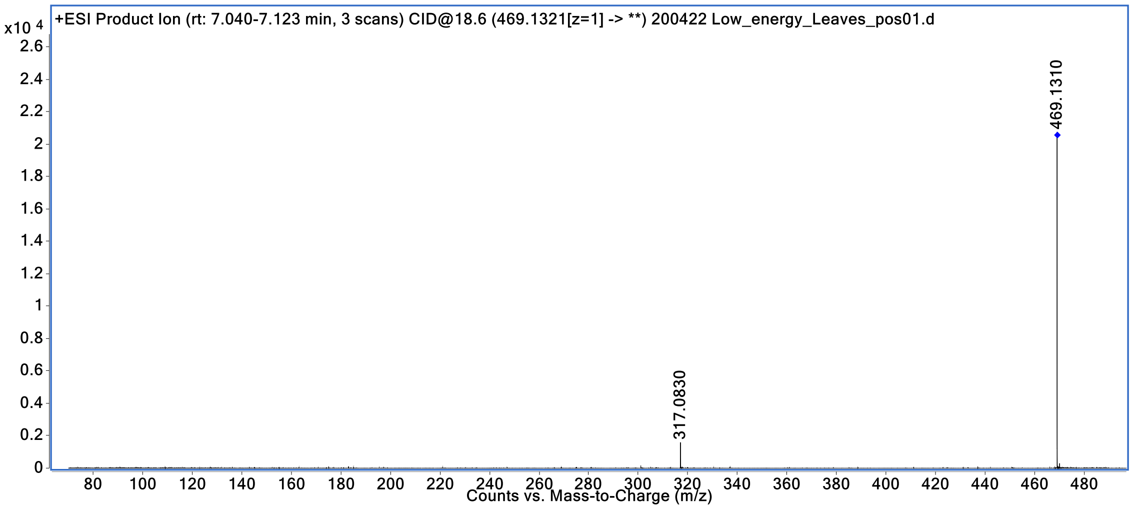

Figure S92. MS2 spectra from [M+Na]+=469.1314, gaultherin (**44**).

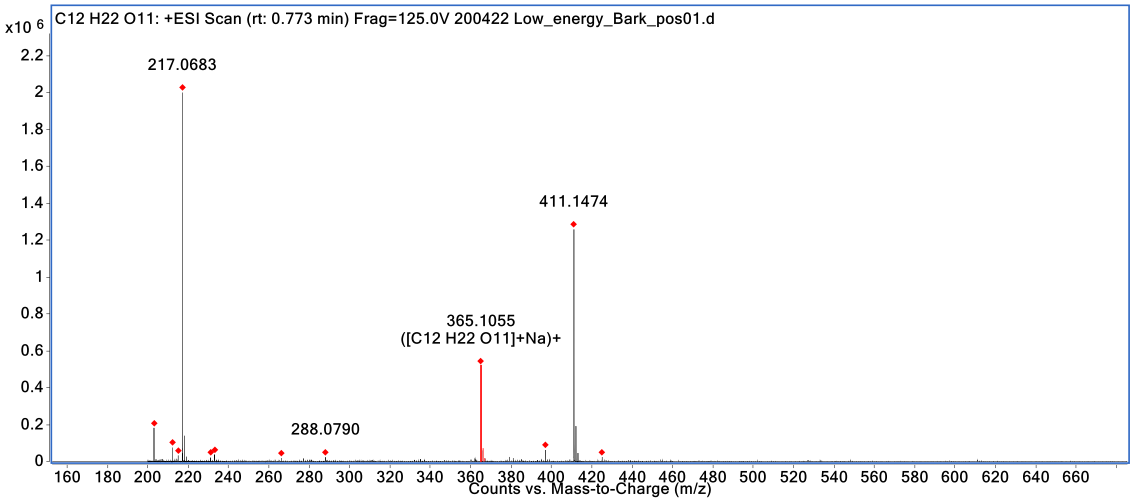

Figure S93. MS1 spectra from [M+H]+= 365.1055, sucrose (**45**).

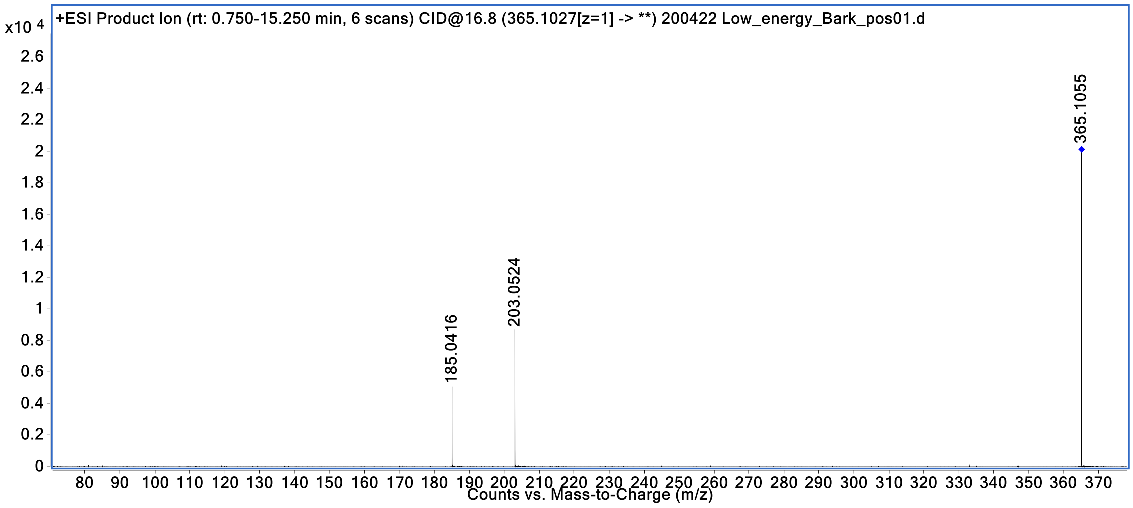

Figure S1. MS2 Spectra from [M+H]+=365.1055, sucrose (**45**).

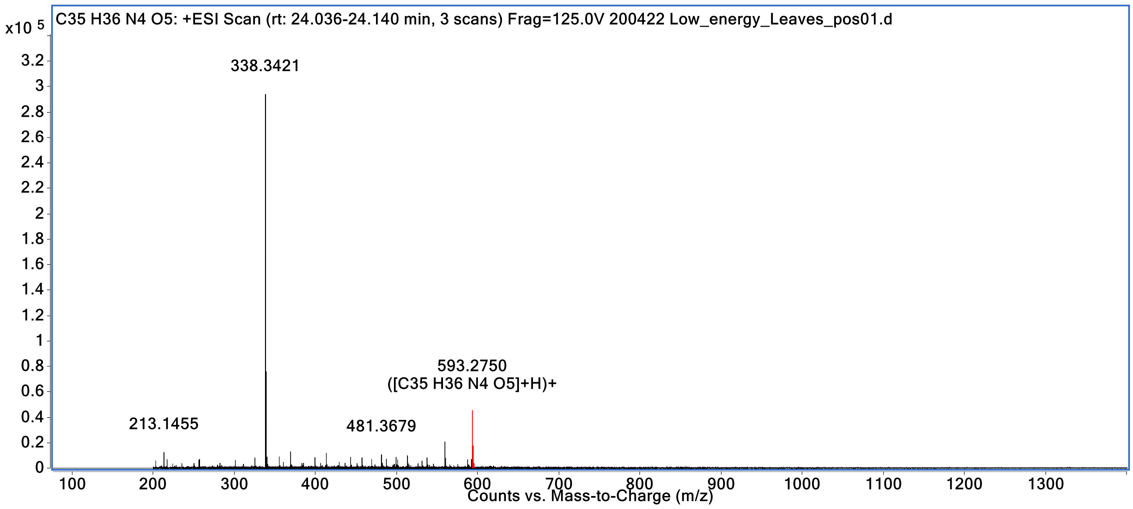

Figure S94. MS1 Spectra from [M+H]+=593.2750, pheophorbide A (**46**).

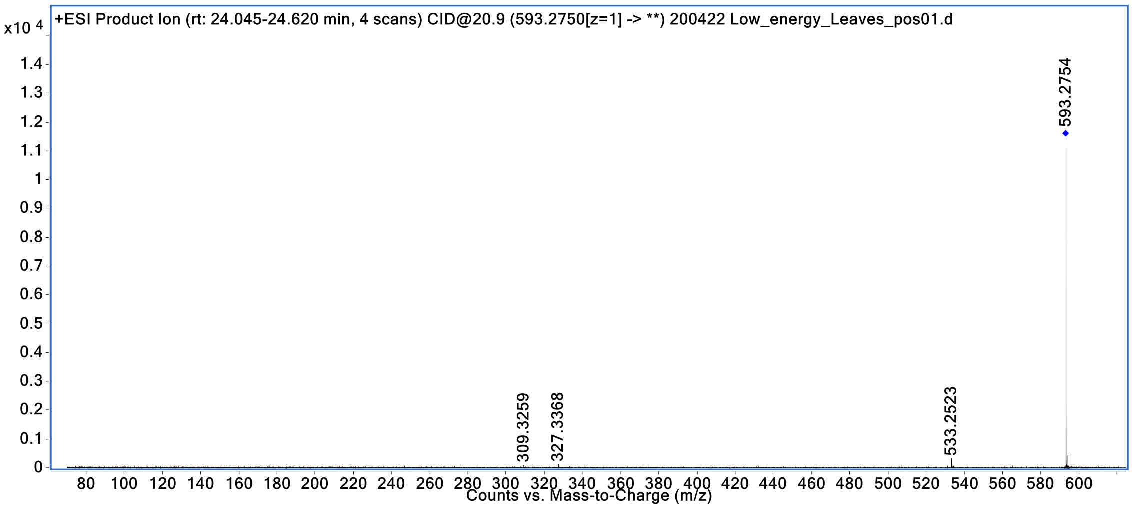

Figure S95. MS2 Spectra from [M+H]+=593.2750, pheophorbide A (**46**).

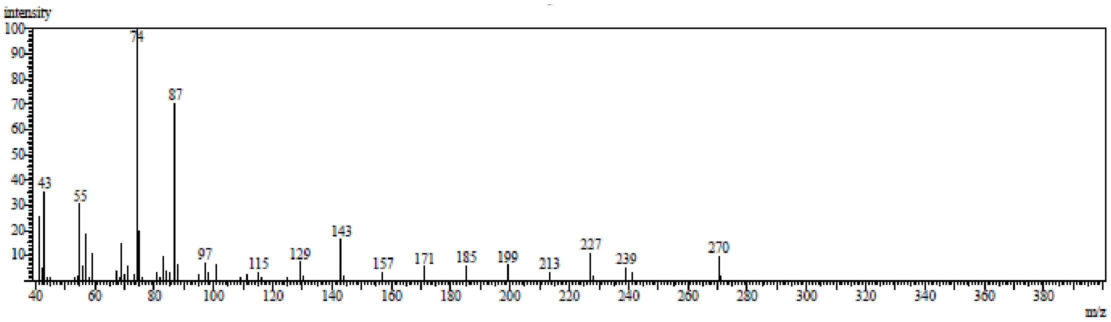

Figure S96. MS spectra (GC-MS) from palmitic acid (methyl ester) (**47**).

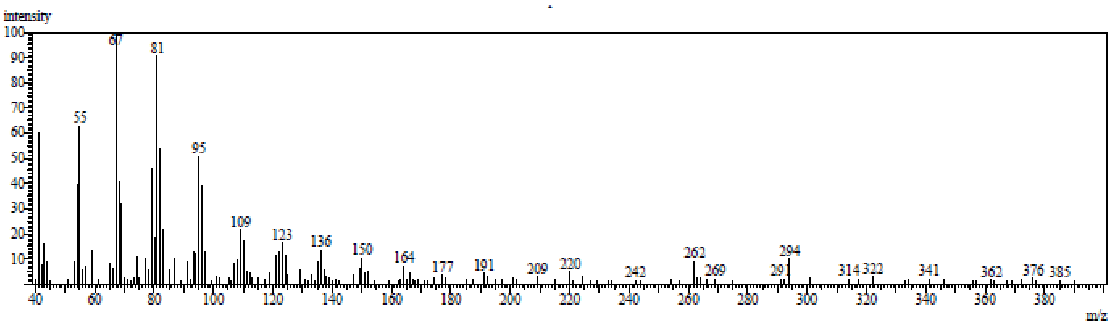

Figure S97. MS spectra (GC-MS) from linoleic acid (methyl ester) (**48**).

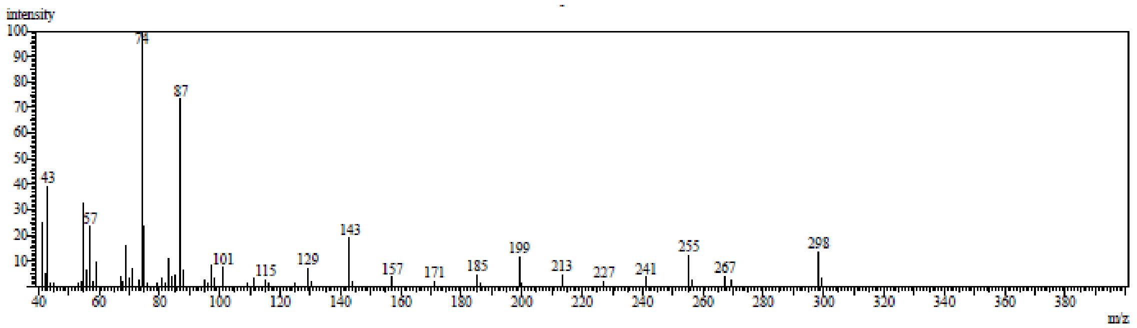

Figure S98. MS spectra (GC-MS) from stearic acid (methyl ester) (**49**).

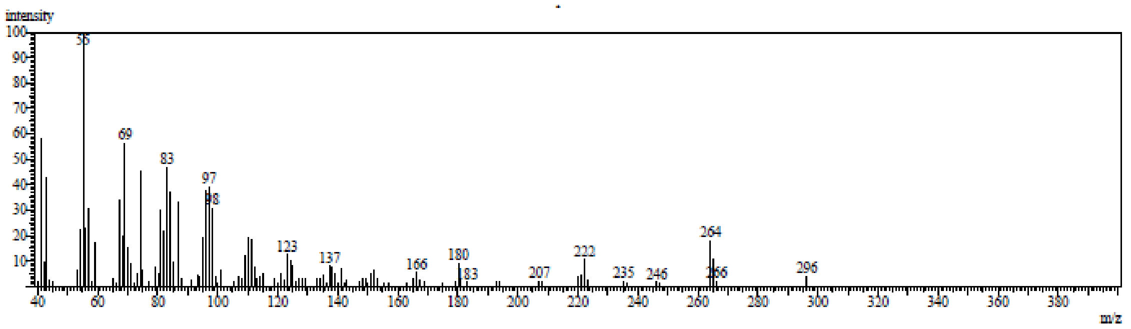

Figure S99. MS spectra (GC-MS) from vaccenic acid (methyl ester) (**50**).
